# ﻿A Japan-focused review of East Asian *Acicnemis* Fairmaire, 1849 (Coleoptera, Curculionidae) reveals cryptic diversity in a well-studied region

**DOI:** 10.3897/zookeys.1259.164450

**Published:** 2025-11-10

**Authors:** Jake H. Lewis, Hiroaki Kojima, Erin O. Campbell

**Affiliations:** 1 Department of Biological Sciences, University of Alberta, Edmonton, Alberta T6G 2E9, Canada University of Alberta Alberta Canada; 2 Okinawa Institute of Science and Technology Graduate University, 1919-1 Tancha, Onna-son, Kunigami-gun, Okinawa 904-0495, Japan Okinawa Institute of Science and Technology Graduate University Tancha Japan; 3 Laboratory of Entomology, Tokyo University of Agriculture, 1737 Funako, Atsugi, Kanagawa, 243-0034, Japan Tokyo University of Agriculture Atsugi Japan; 4 Ottawa Plant Lab, Canadian Food Inspection Agency, 960 Carling Avenue, Ottawa, Ontario, K1A 0C6, Canada Ottawa Plant Lab, Canadian Food Inspection Agency Ottawa Canada

**Keywords:** Biodiversity, DNA barcoding, island endemism, new species, species discovery, taxonomy

## Abstract

The East Asian species of *Acicnemis* Fairmaire, 1849 (Coleoptera: Curculionidae: Molytinae) are reviewed using a combination of traditional morphological approaches (microscopy, dissections) and DNA barcoding. Three new species, *A. cryptica* Lewis & Kojima, **sp. nov.**, *A. koguma* Lewis & Kojima, **sp. nov.** and *A. squamata* Lewis & Kojima, **sp. nov.** are described from mainland Japan. The former two species are cryptic and sister to more widespread, previously described taxa. Based on examination of name-bearing type material and morphological considerations *Acicnemis cruciata* Kleine, 1924 **syn. nov.** is a junior subjective synonym of *Acicnemis postica* Hubenthal, 1919, *Acicnemis yakushimana* Morimoto & Miyakawa, 1995 **syn. nov.** is a junior subjective synonym of *A. dividicincta* Morimoto & Miyakawa, 1995, and *Acicnemis dorsonigrita* Voss, 1941 **syn. nov.** is a junior subjective synonym of *Acicnemis palliata* Pascoe, 1872. Lectotypes are designated for *Acicnemis albofasciata* (Ter-Minasian, 1953), *A. laeta* Hubenthal, 1919, *A. postica* Hubenthal, 1919 and *A. sauteri* Hubenthal, 1919. Important morphological characters used in *Acicnemis* species identification are reviewed and a key to the East Asian species is provided. DNA (CO1) barcoding is used to supplement our morphology-based species hypothesis, and the first published barcodes for most species covered here are presented.

## ﻿Introduction

Weevils (Coleoptera: Curculionoidea) are one of the most diverse (ca 62,000 species) and economically important animal groups, but remain poorly studied throughout much of Asia (Oberprieler et al. 2014; Sprick and Floren 2018). The diverse genus *Acicnemis* Fairmaire, 1849 (Curculionidae: Molytinae) contains more than 220 described species distributed across the Eastern Palearctic, Oriental, and Australasian Regions (Hubenthal 1919a, b; Zimmerman 1967; Kojima and Morimoto 2004; Pullen et al. 2014). Members of *Acicnemis* are usually cryptically colored and often bear erect scales dorsally that enhance their disruptive patterning. The genus *Acicnemis* is placed in the tribe Trachodini with four other genera, *Karekizo* Morimoto, 1962 (2 spp.), *Ochodontus* Desbrochers des Loges, 1897 (1 sp.), *Semelima* Pascoe, 1872 (1 sp.), and *Trachodes* Germar, 1824 (ca 30 spp.), which are also all Old World genera (Morimoto and Miyakawa 1995; Alonso-Zarazaga and Lyal 1999; Alonso-Zarazaga et al. 2023). The core genera of Trachodini, *Acicnemis* and *Trachodes*, have a complex taxonomic history (see Hubenthal 1919a; Zimmerman 1967), and have typically been distinguished based on genital (in part) and hindwing morphology (fully developed hindwings in *Acicnemis*, reduced in *Trachodes*) (Morimoto and Miyakawa 1995). Hubenthal (1919a, b) compiled the most comprehensive key to *Acicnemis* and is responsible for describing many species; however, those works lack illustrations and are outdated as many new synonyms and species have since been discovered. Subsequent works have largely been disjunct, single species descriptions and rarely include keys, illustrations, or useful comparative notes (e.g., Kleine 1924; Voss 1941; Zimmerman 1943; Ter-Minasian 1953); however, a few more recently published reviews do include comprehensive illustrative keys for restricted regions, such as South Korea (Park et al. 2009) and Japan (Morimoto and Miyakawa 1995).

The genus *Acicnemis* is greatly in need of an exhaustive revision, and such a project will undoubtedly uncover many new species and synonyms. However, this would be an enormous undertaking given the high diversity in the genus (> 220 described species), lack of modern treatments of the group, and the costly necessity of viewing and dissecting type material that is deposited widely in natural history collections across Europe and Asia. In response to these challenges, we develop a preliminary, Japan-focused review of the East Asian species of *Acicnemis*, building on the work of Morimoto and Miyakawa (1995) that covered only the Japanese species. In addition to Japan, we now also include species from Taiwan, the Korean Peninsula, and China, as a foundation for future work on species in Southeast Asia, the epicenter of Trachodini diversity. We review important external morphological and genital characters, present a key to the East Asian species, and use DNA (CO1) barcoding to supplement our morphology-based species hypotheses.

## ﻿Materials and methods

### ﻿Specimen acquisition and general methodology

Specimens were examined from the following collections:

**NHMUK** Natural History Museum, London, England

**CMIC** Natural History Museum and Institute, Chiba, Japan

**CMNC** Canadian Museum of Nature, Gatineau, Canada

**CNCI** Canadian National Collection of Insects, Ottawa, Canada

**ELKU** Entomological Lab of Kyushu University, Fukuoka, Japan

**HUM** Hokkaido University Museum, Sapporo, Japan

**KPMNH** Kanagawa Prefectural Museum of Natural History, Odawara, Japan

**KUM** Kyushu University Museum, Fukuoka, Japan

**LBM** Lake Biwa Museum, Kusatsu, Japan

**NHMB** Natural History Museum Basel, Basel, Switzerland

**NMNS** National Museum of Nature and Science, Tsukuba, Japan

**OIST** Okinawa Institute of Science and Technology, Tancha, Japan

**RUMC** Ryukyu University Museum Collection, Nishihara, Japan

**SDEI** Senckenberg Deutsches Entomologisches Institut, Müncheberg, Germany

**SNSD** Senckenberg Naturhistorischen Sammlungen Dresden, Dresden, Germany

**TUA** Tokyo University of Agriculture, Atsugi, Japan

**USNM** National Museum of Natural History, Washington, U.S.A.

**ZIN** Zoological Institute of Russian Academy of Sciences, St. Petersburg, Russia

**ZMH** Zoologisches Museum Hamburg, Hamburg, Germany

We first created an exhaustive list of all described *Acicnemis* by reviewing the literature and examining as many major specimen collections as possible. We also examined name-bearing type material for all species presented here, as well as > 90% of the *Acicnemis* species not treated here. We base our taxonomic decisions on the phylogenetic species concept (Wheeler and Platnick 2000), and provide unique combinations of both morphology and molecular (CO1) characters that define the species covered in this paper. Examined specimens without institutional unique specimen identifier (USI) labels were assigned labels that read in the form: JHL_AREV_###, JHL_ACITAI_###, or JHL_DNA_###. In some older collections, we encountered that multiple specimens were glued to the same point with a single data label; in these cases we assigned a single USI to represent the entire lot of specimens on the same pin. Specimens were relaxed and dissected by removing the abdomen, and genitalia were cleared in a solution of KOH and water. The genitalia were placed in a small vial of glycerin and attached to the same pin as their respective dissected specimens. Images of genitalia were taken with a Nikon DS-Fi3 camera through a Nikon SMZ18 stereomicroscope using NIS-Elements D v. 5.41.00 (Nikon Corporation, Yokohama, Japan). All other images were taken under a Leica M205 C microscope with a Leica DMC 5400 camera and stacked using Leica Application Suite (Leica Microsystems, Wetzlar, Germany). We follow the morphological terminology of Oberpreiler et al. (2014). X-ray μCT is used to visualize cryptic morphology in a few species; we use the same methodology as Lewis et al. (2024) with scan settings as follows for the two specimens: *A. ryukyuana* Lewis, 2023 (OKENT0053498) [Mag: 4X, Exposure: 1.1 sec., Source dist.: 20.19 mm, Detector dist.: 8.65 mm, Voltage: 50 kV, Power: 4 W] and *A. albofasciata* (Ter-Minasian, 1953) (JHL_AREV_782) [Mag: 0.4X, Exposure: 3 sec., Source dist.: 15.04 mm, Detector dist.: 113 mm, Voltage: 50 kV, Power: 4 W].

### ﻿DNA barcoding

The widely used barcode gene cytochrome c oxidase subunit I (CO1) has been successfully used to delineate weevils, and even short fragments of this gene can be informative in species delineation (e.g., Lewis et al. 2024). We sequenced a short ~300 bp long fragment of cytochrome c oxidase subunit I (CO1) from twelve *Acicnemis* species (*A. albofasciata*, *A. azumai* Morimoto & Miyakawa, 1995, *A. dividicincta* Morimoto & Miyakawa, 1995, *A. exilis* Morimoto & Miyakawa, 1995, *A. laeta* Hubenthal, 1919, *A. luteomaculata* Morimoto & Miyakawa, 1995, *A. maculaalba* Roelofs, 1875, *A. nohirai* Morimoto & Miyakawa, 1995, *A. postica* Hubenthal, 1919, *A. ryukyuana*, *A. sauteri* Hubenthal, 1919, and *A. squamata* sp. nov.) to illustrate the utility of this shortened CO1 fragment in weevil delineation, as well as to complement our morphology-based species hypotheses. In total, we sequenced 24 *Acicnemis* specimens and six outgroup taxa. For each *Acicnemis* species, we made efforts to include at least two specimens from different localities to account for intraspecific CO1 variation.

DNA was extracted using the non-destructive methods of Oberacker et al. (2019). The forward MCO-IntF (GGWACWGGWTGAACWGTWTAYCCYCC) (Leray et al. 2013) and reverse Fol-degen-rev (TANACYTCNGGRTGNCCRAARAAYCA) (Yu et al. 2012) primers were used in a PCR mix with the following thermal profile: Initial (95 °C – 15:00 min.), Denature (94 °C – 0:30 min.), Annealing (46 °C – 1:30 min.), Extension (72 °C – 1:30), Repeat 35X, Final hold (4 °C – Infinite). The PCR products were sequenced with Illumina MiSeq using 600-cycle v3 kits. The CO1 fragments were processed and assembled in Geneious Prime (v. 11.0.14.1; Dotmatics, Boston, Massachusetts, United States of America). We used BLAST to confirm that non-target (non-weevil) DNA was not erroneously used and constructed neighbor-joining trees to ensure that members of the same species (judged by morphology) clustered together. DNA sequence data was aligned using MUSCLE (v. 3.8.425 with default settings; Edgar 2004) using default settings and quality-controlled by examining translated amino-acid alignments. MEGA 11 (v. 11.0.13; Tamura et al. 2021) was used to calculate pairwise K2P distances for all sequences and were used to calculate intra- and interspecific ranges and averages in the sequenced *Acicnemis* species.

For the gene-tree, ModelFinder (Kalyaanamoorthy et al. 2017) was used to select a substitution model (GTR+F+I+G4). A Maximum Likelihood (hereafter ML) tree of the twelve *Acicnemis* species was constructed in IQ-TREE (v. 1.6.12; Nguyen et al. 2015) with a heuristic search of 100,000 initial trees. Standard nonparametric bootstrap (hereafter BS) values were calculated from 1000 replicates. For outgroup taxa, we used the molytine weevil species *Aphanerostethus japonicus* Lewis & Kojima, 2024 (Cryptorhynchini Schoenherr, 1825), *Colobodes ornatoideus* Morimoto, 1988 (Sophrorhinini Lacordaire, 1865), *Deretiosus albicaudatus* Morimoto, 1988 (Sophrorhinini Lacordaire, 1865), *Ectatorhinus adamsii* Pascoe, 1871 (Ithyporini Lacordaire, 1865), *Protacallodes ryukyuensis* Morimoto, 2011 (Ithyporini Lacordaire, 1865), and Tylodina (tribe) sp. 1 (Cryptorhynchini Schoenherr, 1825). All CO1 barcodes were uploaded to GenBank (Accession numbers: PP110445.1, PP110471.1, PP110442.1, PP110479.1, PP110447.1, PP110480.1, PV255624–PV255644). Three specimens that we examined were sequenced as part of another project and their sequence data were retrieved from BOLD Systems (Accession numbers: MEBLA191-14, OWEVG891-15, VVGPL2845-15).

## ﻿Results

### ﻿General remarks

Based on an examination of 934 specimens from 19 major Asian, European, and North American natural history collections, we documented 25 *Acicnemis* species from East Asia including three hitherto overlooked new species from mainland Japan: *A. cryptica* Lewis & Kojima, sp. nov., *A. koguma* Lewis & Kojima, sp. nov., and *A. squamata* Lewis & Kojima, sp. nov. The former two new species (*A. cryptica* and *A. koguma*) are cryptic and sister to more widespread, previously described species (*A. albofasciata* and *A. kiotoensis*, respectively). Based on morphological considerations, we also propose the following subjective synonymies: *Acicnemis cruciata* Kleine, 1924 syn. nov. under *Acicnemis postica* Hubenthal, 1919, *Acicnemis yakushimana* Morimoto & Miyakawa, 1995 syn. nov. under *A. dividicincta* Morimoto & Miyakawa, 1995, and *Acicnemis dorsonigrita* Voss, 1941 syn. nov. under *Acicnemis palliata* Pascoe, 1872.

**Figure 1.**
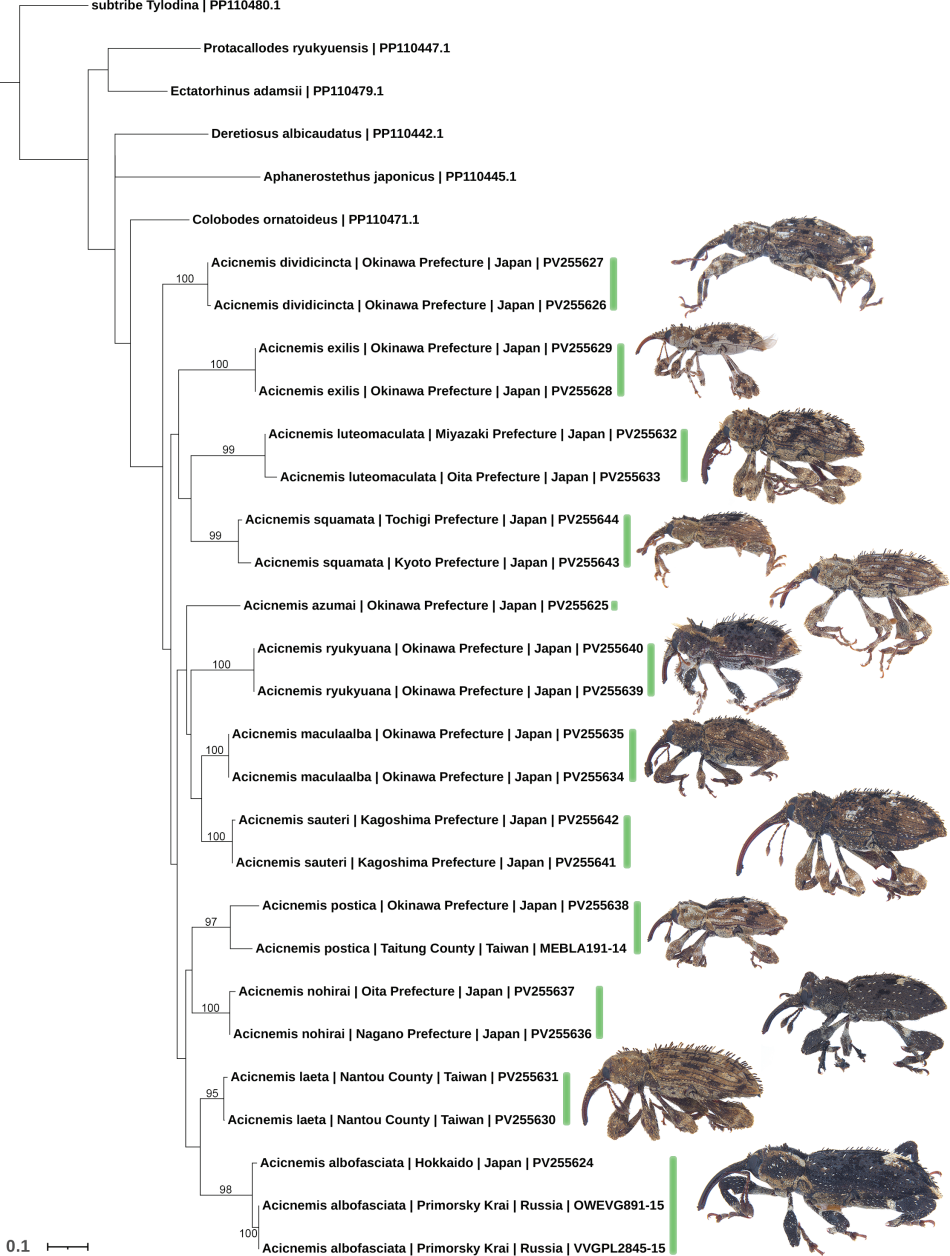
Maximum likelihood tree of twelve *Acicnemis* species based on a 300 base-pair long CO1 gene fragment constructed in IQ-TREE v. 1.6.12 using a GTR+F+I+G4 model. Branch support values represent standard nonparametric bootstraps (1000 replicates). Bootstrap values less than 75 are not displayed on the tree. The codes MEBLA191-14, VVGPL2845-15, and OWEVG891-15 are BOLD Systems accession numbers, whereas all other codes are GenBank accession numbers. Associated *Acicnemis* weevil figures on the right of the tree are approximately to scale.

Within the 300 bp CO1 alignment, IQ-TREE identified 125 parsimony informative sites. In our CO1 gene tree, specimens of all twelve *Acicnemis* species clustered together (BS ≥ 95) as expected based on morphology (Fig. 1). Nodes beyond the species level were weakly supported (BS ≤ 55); however, this is not unexpected given that we only analyzed a single 300-bp long fragment of a relatively rapidly evolving mitochondrial marker (see Townsend et al. 2012). Although a proper phylogenetic analysis (in prep) is clearly required to make robust statements about phylogeny, the weak association (BS: 38) between species such as *A. sauteri* and *A. maculaalba* is expected as these form a species complex of brown, white-spotted species (see *A. maculaalba* remarks section) and are frequently confused with each other. The K2P distances (see Suppl. material 1) show little intraspecific variation within *Acicnemis* species (range: 0–0.10193; t-distributed 95% confidence interval: 0.0199 ± 0.0198), and a trend of greater K2P distance in species where representative specimens were collected at distant localities (i.e., different prefectures or countries such as *A. postica*). Interspecific K2P distances between *Acicnemis* species ranged from 0.10771–0.27405 (t-distributed 95% confidence interval: 0.1869 ± 0.0036), and were lowest between the related *A. maculaalba* and *A. sauteri*.

### ﻿Morphology

The East Asian species of *Acicnemis* are relatively uniform morphologically across the genus, but do differ prominently in scale color and pattern, which is highly conserved within species. They also markedly differ in male genital morphology, and examination of the genitalia is critical for identification of some cryptic species covered here (e.g., *A. albofasciata* and *A. cryptica*). There are, however, several other important external morphological characters that are critical to *Acicnemis* species identification that we summarize below.

#### ﻿Third tarsomeres

**Figure 2.**
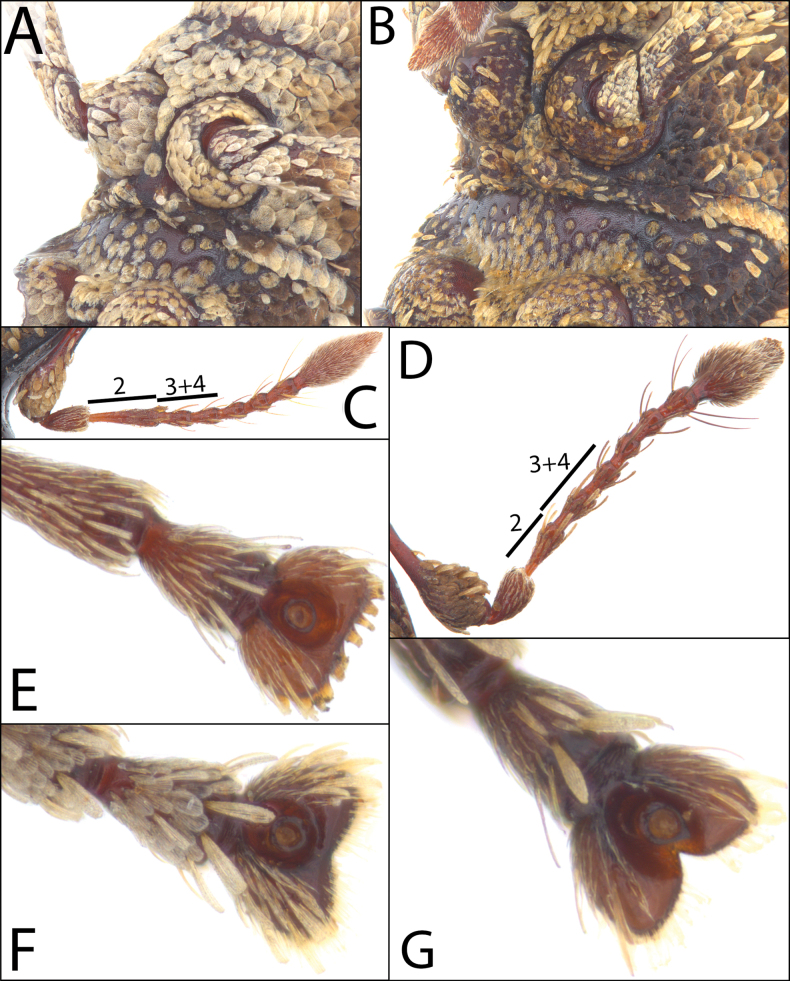
Diagnostic morphological characters in *Acicnemis*. A. *A. azumai* Morimoto & Miyakawa, 1995 (JHL_ACITAI_081) showing lack of prominent posterior prosternal projections; B. *A. laeta* Hubenthal, 1919 (JHL_AREV_533) showing prominent prosternal projections; C. *A. kiotoensis* Nakane, 1963 (JHL_AREV_982) showing antenna with the second funicular antennomere longer than antennomeres 3 + 4; D. *A. koguma* Lewis & Kojima, sp. nov. (JHL_SYN_141) showing antenna with the second funicular antennomere shorter than antennomeres 3 + 4; E. *A. luteomaculata* Morimoto & Miyakawa, 1995 (JHL_AREV_189) showing truncate third tarsomere; F. *A. ryukyuana* Lewis, 2023 (JHL_ACI_011) showing weakly emarginate third tarsomere; G. *A. maculaalba* Roelofs, 1875 (JHL_AREV_577) showing strongly emarginate third tarsomere.

The third tarsomeres are emarginate (e.g., Fig. 2G) in most *Acicnemis* species (e.g., *A. albofasciata*, *A. azumai*); however, a few species covered here have truncate (*A. luteomaculata* (Fig. 2E) and *A. koguma* sp. nov.) or weakly emarginate third tarsomeres (*A. ryukyuana*) (Fig. 2F).

#### ﻿Antennomeres

The proportions of funicular antennomere lengths vary between *Acicnemis* species. For example, in *A. kiotoensis* Nakane, 1963 the second funicular antennomere is longer than funicular antennomeres 3+4 (Fig. 2C); however, in the closely related species *A. koguma* the second funicular antennomere is shorter than funicular antennomeres 3+4 (Fig. 2D).

#### ﻿Pronotum

**Figure 3.**
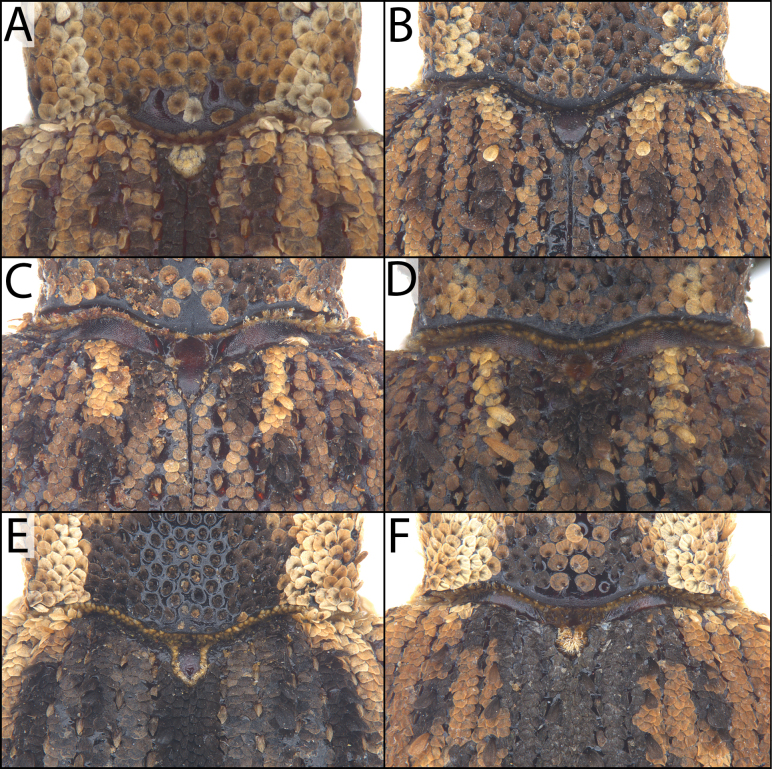
Scutella and pronota of various *Acicnemis* species. A. *A. azumai* Morimoto & Miyakawa, 1995 (JHL_ACITAI_081) showing two bare (unscaled) tubercles baso-medially on the pronotum; B. *A. kiotoensis* Nakane, 1963 (JHL_AREV_982) showing a broad, unscaled scutellum; C. *A. koguma* Lewis & Kojima, sp. nov. (JHL_SYN_141) showing a bare, but more slender scutellum than *A. kiotoensis*; D. *A. maculaalba* Roelofs, 1875 (JHL_AREV_577) showing a small scutellum covered in scales; E. *A. palliata* Pascoe, 1872 (JHL_AREV_779) showing a larger scutellum that is shiny and bare dorsally, and surrounded along the perimeter by yellow scales; F. *A. shigematsui* Morimoto & Miyakawa, 1995 (JHL_ACITAI_052) showing a smaller scutellum covered in scales.

One species covered here (*A. azumai*) has unscaled, baso-medial tubercles on the pronotum (Fig. 3A). These are diagnostic for this species and can be used to quickly distinguish *A. azumai* from the superficially similar *A. laeta* and much smaller species *A. exilis*. Furthermore, some species have an impunctate longitudinal midline on the pronotum (e.g., *A. koguma*, some specimens of *A. albofasciata* and *A. cryptica*).

#### ﻿Prosternum

The Taiwanese species *A. laeta* has diagnostic prosternal projections that lie posterior to the fore-coxae (Fig. 2B). This rare character is not found in any other species covered here but is shared with a few Southeast Asian species.

#### ﻿Scutellum

The morphology of the scutellum is extremely variable and very important in *Acicnemis* species identification. Some species have a large, shiny scutellum (e.g., *A. palliata* Pascoe, 1872) (Fig. 3E), while others have a heavily scaled one (e.g., *A. azumai*) (Fig. 3A). In some instances, scutellum morphology can be used to easily differentiate similar species (e.g., *A. kiotoensis* vs. *A. maculaalba* Roelofs, 1875) (Fig. 3B, D).

#### ﻿Sclerolepidia

**Figure 4.**
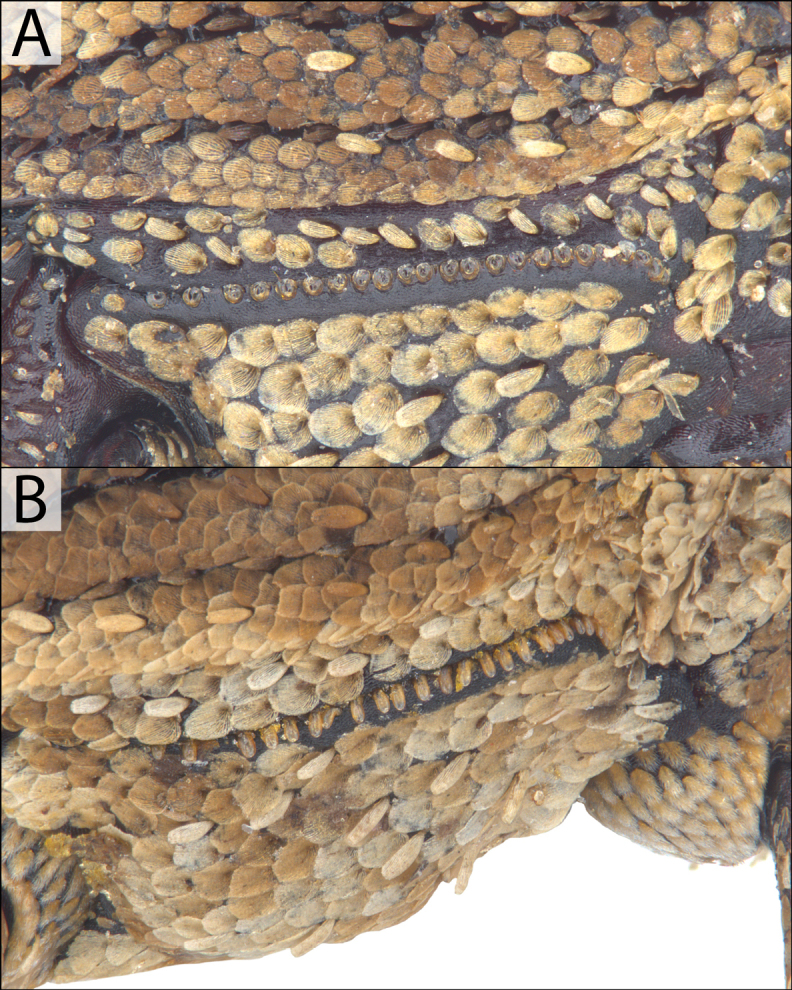
Sclerolepidia in *Acicnemis*. A. *A. kiotoensis* Nakane, 1963 (JHL_AREV_982) showing low, rounded sclerolepidia; B. *A. palliata* Pascoe, 1872 (JHL_AREV_779) showing protruding, erect sclerolepidia.

Variation in the sclerolepidia has been documented in *Acicnemis* previously (Lyal et al. 2006) (Fig. 4). Although we examined our two newly described cryptic species and their associated sister species (*A. albofasciata* – *A. cryptica* and *A. kiotoensis* – *A. koguma*) for variation in sclerolepidia morphology, we did not observe any significant differences. We assert that the sclerolepidia are useful primarily for broadly placing the species into groups, but not useful for separating closely related species.

#### ﻿Erect elytral scales

Many *Acicnemis* species have erect elytral scales that vary in length, position, and patterning. In particular, some species only have these erect scales on the odd-numbered elytral intervals (e.g., *A. shigematsui* Morimoto & Miyakawa, 1995), while others have erect scales on the odd-numbered intervals and the second interval (e.g., *A. luteomaculata*). Some East Asian species also have exceptionally long erect elytral scales (e.g., *A. ryukyuana*).

#### ﻿Pronotum punctation (cryptic morphology; requires X-ray μCT)

Pronotum punctation differs between species in some instances; however, this character is generally not visible as the pronotum is often covered by scales. We used X-ray μCT (as in Lewis et al. 2024) to strip away the pronotal scales in *A. ryukyuana* and *A. albofasciata* and found differences in puncture spacing (and pronotal sculpturing). In *A. ryukyuana* (Fig. 32A) the pronotum is smooth and the punctures are well spaced, whereas in *A. albofasciata* (Fig. 32B) the pronotum is coarsely sculptured and the punctures are closely spaced. We did not encounter any differences between closely related sister species (e.g., *A. albofasciata* – *A. cryptica* or *A. kiotoensis* and *A. koguma*), indicating that this pronotum punctation and our X-ray μCT methodology is only useful for more delineating more distantly related *Acicnemis* species pairs.

#### ﻿Metanotum + dorso-lateral flight muscles (cryptic morphology; requires X-ray μCT)

Using X-ray μCT, we visualized the metanotum and dorso-longitudinal flight muscles (DLFM), which moderately differ in proportion to the elytra in *A. ryukyuana* and *A. albofasciata*. In *A. ryukyuana* (Fig. 32C) the metanotum + DLFM are 30% the length of the elytra, whereas in *A. albofasciata* (Fig. 32D) they are 23% the length of the elytra. Although most *Acicnemis* species share a similar, elongate body profile to *A. albofasciata*, some species are more rounded and compact like *A. ryukyuana*. The evolution of a more compact body plan and abrupt apical elytral declivity in *A. ryukyuana* apparently had little effect on the relative size of the metanotum and DLFM, hence the difference in proportional length between species.

### ﻿Taxonomy

#### ﻿ Acicnemis

Taxon classificationAnimaliaColeopteraCurculionidae

Fairmaire, 1849

D31A9449-1053-581F-892C-FEBD2F63C355

Oplocnemus Dejean, 1835 (Type species: Oplocnemus mucronatus Buquet nomen nudum).
Hoplocnemus Agassiz, 1846 (unjustified emendation of Oplocnemus).
Acienemis Motschulsky, 1863 (incorrect subsequent spelling).
Berethia Pascoe, 1872 (Type species: Berethia medinotata Pascoe, 1872).
Acicnemus Haly, 1890 (incorrect subsequent spelling).

##### Type species.

*Acicnemis variegata* Fairmaire, 1849 (by subsequent designation).

##### Gender.

Feminine.

##### Description.

Body length 2.0–12.0 mm. ***Scale pattern***: Usually cryptically patterned with contrasting brown, black, gray, and dull yellow scales. Pronotum and elytra often with erect scales. ***Head***: Rostrum evenly curved in most species, and variably longer in females than in males; eyes large, ovate, and non-contiguous; antennae with seven-articled funicle. ***Prothorax***: Densely punctate; pronotum often with tufts of erect scales; prosternum lacking well defined prosternal canal. ***Elytra***: Scutellum varied (large, plate-like, naked scutellum in some species, or small and covered in scales in others); erect scales often only on odd-numbered elytral intervals; elytra unmodified in most species, costate in a few; elytra with apical projections in some species (*A. laticollis* Pascoe, 1885); low tubercles along elytral intervals in some species, absent in most; volant, hind wings functional. ***Thorax***: Sclerolepidia varied: prominent and protruding in some species, forming low, indistinct tubercles in others. ***Abdomen***: First and second sternites variably concave medially in males (convex or flat in females). ***Legs***: Femora all bearing a large ventral tooth (simple in most species, serrated in a few); forelegs elongate in some species (e.g., *A. longimana* Hubenthal, 1922); third tarsomeres emarginate (bilobed) in most species, truncate in a few; tarsal claws simple.

##### Distribution.

*Acicnemis* species are broadly distributed across the Palearctic Region (Azerbaijan, China, Georgia, Japan, Korean archipelago, Russia, Taiwan), the Oriental Region (Cambodia, India, Indonesia, Laos, Malaysia, Myanmar, Philippines, Thailand, Vietnam), the Australasian Region (Australia, Fiji, New Guinea, Samoa, Tahiti, Tonga), and apparently also the Afrotropical Region (Nigeria) (Hubenthal 1919a, b; Zimmerman 1967; Alonso-Zarazaga and Lyal 1999; Kojima and Morimoto 2004; Oziegbe and Faluyi 2010; Pullen et al. 2014; Alonso-Zarazaga et al. 2023).

##### Ecology and natural history.

The larvae of Trachodini mine through dead wood (Lyal 2014). Host plant associations are not well known in *Acicnemis*; however, a few records exist (*Acicnemis palliata* Pascoe, 1872 with *Wisteria floribunda* (Willd.) DC. and *W. sinensis* (Sims) DC. (Fabaceae) (Morimoto and Miyakawa 1995; Lee and Morimoto 1996; Zhang 2021); *Acicnemis suturalis* Roelofs, 1875 with *Wisteria brachybotrys* Siebold & Zucc. (Morimoto and Miyakawa 1995; Lee and Morimoto 1996); *Acicnemis crassiusculus* Fairmaire, 1878 with *Hibiscus tiliaceus* L. (1753) (Malvaceae) and *Ludwigia* spp. (Onagraceae) (Zimmerman 1943; Oziegbe and Faluyi 2010); the larvae of one unidentified *Acicnemis* species were taken from a dead stem of *Castanopsis sieboldii* (Makino) Hatus (Fagaceae) (Lee and Morimoto 1996). Some species are attracted to lights (e.g., *A. azumai*) (JHL pers. obs.).

### ﻿Species profiles

#### ﻿ Acicnemis albofasciata

Taxon classificationAnimaliaColeopteraCurculionidae

(Ter-Minasian, 1953)

AF5DBBB6-FD29-5147-8C1E-7F8AB32201D9

**Figure 5.**
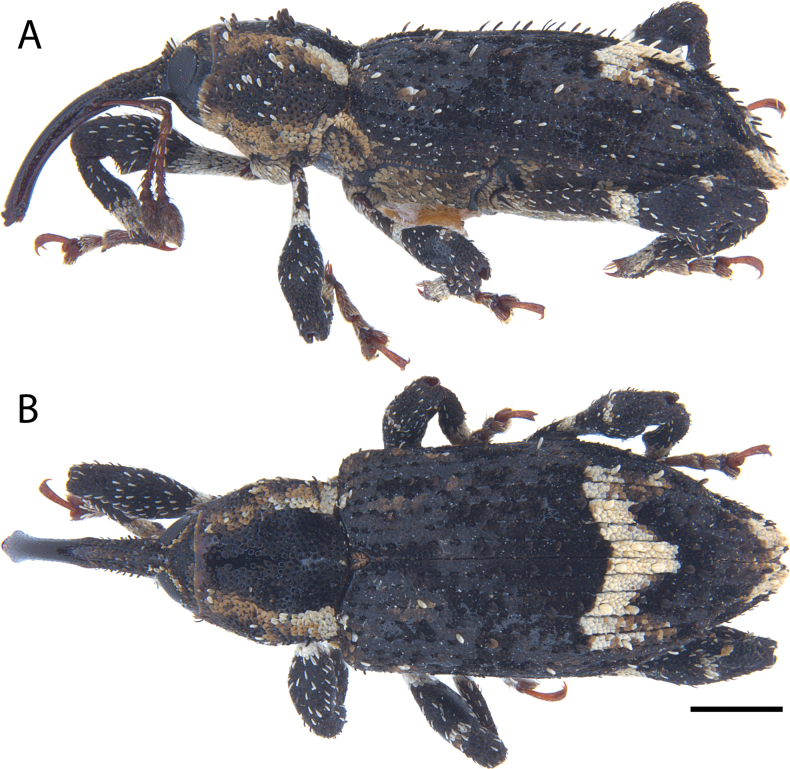
*Acicnemis albofasciata* (Ter-Minasian, 1953) (JHL_AREV_781). A. Lateral; B. Dorsal. Scale bar: 1 mm.

**Figure 6.**
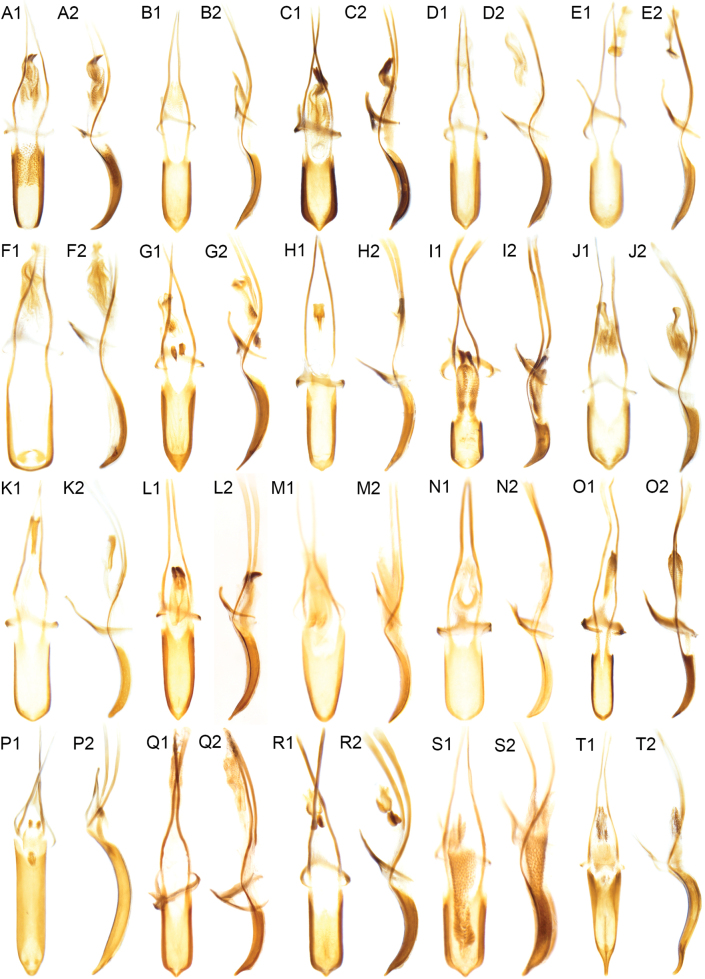
Aedeagi of *Acicnemis*. A1–2. *A. albofasciata* (Ter-Minasian 1953); B1–2. *A. azumai* Morimoto & Miyakawa, 1995; C1–2), *A. cryptica* Lewis & Kojima, sp. nov.; D1–2. *A. dividicincta* Morimoto & Miyakawa, 1995; E1–2. *A. exilis* Morimoto & Miyakawa, 1995; F1–2. *A. kiotoensis* Nakane, 1963; G1–2. *A. laeta* Hubenthal, 1919; H1–2. *A. luteomaculata* Morimoto & Miyakawa, 1995; I1–2. *A. maculaalba* Roelofs, 1875; J1–2. *A. nobilis* Hubenthal, 1919; K1–2. *A. nohirai* Morimoto & Miyakawa, 1995; L1–2. *A. palliata* Pascoe, 1872; M1–2. *A. postica* Hubenthal, 1919; N1–2. *A. ryukyuana* Lewis, 2023; O1–2. *A. sauteri* Hubenthal, 1919; P1–2. *A. shibatai* Voss, 1971; Q1–2. *A. shigematsui* Morimoto & Miyakawa, 1995; R1–2. *Acicnemis* sp. 1; S1–2. *A. squamata* Lewis & Kojima, sp. nov.; T1–2. *A. suturalis* Roelofs, 1875.

Figs 1, 5, 6A1–6, 2, 32B, D

Trachodes albofasciata Ter-Minasian, 1953: 317.
Acicnemis nigra Nakane, 1963: 37.

##### Type material examined

**(Russia: 8; Japan: 173; Total: 181). *Lectotype* (designated here). Russia** • Primorsky Krai, Lyanchihe (26 km from Vladivostok), 19.V.1949, on *Betula manshurica*, ZIN, JHL_AREV_997. ***Paralectotype*. Russia** • Primorsky Krai, Partizansk (formerly Suchan), VIII.1925 (1, ZIN), JHL_AREV_996.

##### Non-type material.

**Russia** • Primorsky Krai, Benevskoye, 43.17°N, 133.76°E, 4.VII.2014, E. Jendek (2, CNCI), CNCCOLVG00007390 (BOLD accession number: VVGPL2845-15), CNCCOLVG00008936 (dissected male, BOLD accession number: OWEVG891-15); • S. Primor’ye, Kedrovaya Pad Reserve, 20.V.–2.VI.1992, T. Nakamura (3, KUM), JHL_AREV_499 (dissected male), JHL_AREV_782, JHL_AREV_787; • Saghalien, Kawakami (present day Sinegorsk), 7.VIII.1922, T. Esaki (1, KUM), JHL_AREV_899; **Japan: Akita Prefecture**: • Tamagawa, Tazawako-machi, 10.VII.1976, S. Miyakawa (12, TUA), JHL_AREV_674 (dissected male), JHL_AREV_811 – JHL_AREV_814, JHL_AREV_935; • Tamagawa Onsen, 10.VII.1976, S. Miyakawa (4, TUA), JHL_AREV_932 (dissected male), JHL_AREV_934; • Senboku, Nyutou-onsen, 10.VI.2015, K. Takahashi (1, KUM), JHL_AREV_887; • Takada-odake, 14.VII.1962, Y. Miyatake (1 dissected male, KUM), JHL_AREV_898; • Sengan-toge, 14.IX.1974, S. Miyakawa (2, TUA), JHL_AREV_965 (dissected male); **Aomori Prefecture**: • Towada, 12.VI.1955, K. Shimoyama (1 dissected male, ZMH), ZMH 837198; • Nurukawa, 12.VIII.1950, K. Shimoyama (1, ZMH), ZMH 837199; • Kobokutai, 5.VIII.1937, K. Shimoyama (1, ZMH), ZMH 837200; • Yunomata, Ohata-machi, Shimokita Penn., 14–30.VII.1956, K. Morimoto (7, KUM), JHL_AREV_543 (dissected male), JHL_AREV_793 – JHL_AREV_797, JHL_AREV_846; **Fukushima Prefecture**: • Tateiwa, 22–23.VI.1984, A. Saito (2, CMIC), CBM–ZI 68470 (dissected male), CBM–ZI 68471; • Minami-aizu-machi, Yaso, 24.VI.2020, T. Saeki (5, KUM), JHL_AREV_882 – JHL_AREV_886; • Shindenhara, 28.V.1979, K. Emoto (1 dissected male, KUM), JHL_AREV_850; **Gifu Prefecture**: • Gifu (1, NHMB), JHL_SYN_192; • Hirayu, 29.VII.–28.VIII.1959, M. Sato (2, KUM), JHL_AREV_102, JHL_AREV_865; • Yunohana, 22.VI.1956 (1 dissected male, CMNC), JHL_AREV_112; **Gunma Prefecture**: • Sugenuma, 10–11.VII.1975, H. Irie (1, KUM), JHL_AREV_869; • Nikamatazawa, Mt. Shirane, 16–18.VII.1975, H. Irie (1 dissected male, KUM), JHL_AREV_918; • Fujimi-toge, 4.VIII, M. Samejima (8, TUA), JHL_AREV_961 (dissected male), JHL_AREV_962, JHL_AREV_964; • **Hokkaido**: “Hokkaido”, “6.19.53”, “Arakawa” (1, NHMB), JHL_SYN_190; • Sounkyo, 11.VII1986, S. Nomura (3, KUM), JHL_SYN_189; • Aizankei, 29.VII.1964 (1, KUM), JHL_AREV_103; • Aizankei, Mt. Daisetsu, 27–31.VII.1955, K. Morimoto & S. Kimoto (14, KUM), JHL_AREV_501 (dissected male), JHL_AREV_798, JHL_AREV_799, JHL_AREV_801 – JHL_AREV_810, JHL_AREV_855; • Teshio, 4.VII.1916, T. Isshiki (1 dissected male, HUM), JHL_AREV_101; • Ashoromura (Tokachi), 29.VII.–23.VIII.1949, R. Matsuda (5, KUM), JHL_AREV_781, JHL_AREV_888 – JHL_AREV_891; • Mitsumata-rindo, Kamishihoro-cho, 25.VI.1986, S. Miyakawa & K. Morimoto (1, TUA; • 2, KUM), JHL_AREV_542, JHL_AREV_815, JHL_AREV_816; • Ashorobuto, Ashoro-gun, 24–31.V.1957, M. Takahashi (3, KUM), JHL_AREV_480 (dissected male), JHL_AREV_847, JHL_AREV_848; • Mt. Daisetsuzan, 26.VII.1953, Y. Kurosawa (2, NMNS), JHL_AREV_852, JHL_AREV_853; • Daisetsuzan, 1.VIII.1962 (3, KUM), JHL_ACITAI_008 (dissected male), JHL_AREV_104, JHL_AREV_105; • Jushichinosawa, Mt. Nipesotsu, 6.VIII.1976, H. Irie (1, KUM), JHL_AREV_856; • Nakayama-toge (near Sapporo), 22.VI.1986, K. Morimoto (1, KUM), JHL_AREV_857; • Bibai, 12.V.1962, K. Kamijo, collected from *Populus* sp. (1, KUM), JHL_AREV_858; • Oyukiyama, 31.VII.1952, Yoshida (1, KUM), JHL_AREV_859; • Aizankei, Antaroma, 31.VII.1952, T. Shirozu (1, KUM), JHL_AREV_861; • Lake Akan, Kushiro, 8.VI.1957, M. Takahashi (1, KUM), JHL_AREV_862; • Wassakanai, Sohya-shichou, 26.VII.1991, S. Ohmomo (1, KUM), JHL_AREV_863; • Nishiashoro (Tokachi), 17.VIII.1949, R. Matsuda (1, KUM), JHL_AREV_864; • Yukomanbetsu, Mt. Daisetsu, 25.VII.1955, K. Morimoto (1, KUM), JHL_AREV_866; • Horoka, Kamishihoro-cho, 17.VII.1976, H. Irie (1, KUM), JHL_AREV_867; • Kamiotoineppu (Teshio), 24.VIII.1922, T. Esaki (4, KUM), JHL_AREV_868, JHL_AREV_877 – JHL_AREV_879; • Kawakami (near Honbetsu), 17–27.VII.1953, Y. Hirashima (1, KUM), JHL_AREV_870; • Aizankei, 19.VII.1962, Y. Miyatake (1, KUM), JHL_AREV_871; • Aizankei (Ishikari), 29.VII.1952, T. Shirozu (5, KUM), JHL_AREV_872 – JHL_AREV_876; • Nakarikubetsu, Rikubetsu Town, Asyoro County, 24.VII.2017, K. Narita (2, KUM), JHL_AREV_880, JHL_AREV_881; • Meakan-dake, Akan National Park, 5.VII.1958, S. Miyamoto (4, KUM), JHL_AREV_892 – JHL_AREV_895; • Uenae, Tomakomai City, 15.VIII.1989, H. Kojima (6, TUA), JHL_AREV_922 (dissected male), JHL_AREV_910 – JHL_AREV_914; • Mt. Karibayama, Shimamaki, 24–27.VII.1989, H. Kojima (2, TUA), JHL_AREV_924, JHL_AREV_926; • Mt. Daisengen, Hakodate City, 23.VII.1989, H. Kojima (1, TUA), JHL_AREV_925; • Poroshirisanso, Hidaka, 5.VIII.1988, H. Kojima (2, TUA), JHL_AREV_927, JHL_AREV_928; • Kamishihoro-chou, Mitsumata, 17.VII.2014, S. Yamamoto, JHL_DNA_154 (GenBank accession number: PV255624) (1, KUM); **Ibaraki Prefecture**: • Mt. Tsukuba, 22.VIII.1977, A. Tanaka (1, HUM), JHL_AREV_854; **Ishikawa Prefecture**: • Mt. Hakusan, 18.V.1979, I. Togashi & D.R. Smith (1 dissected male, USNM), JHL_AREV_985; **Iwate Prefecture**: • Iwate, VI.1936, H. Yamamoto (2, NHMB), JHL_SYN_188, JHL_SYN_191; **Miyagi Prefecture**: • Nuruyu, Hanayama mura, 2–6.VIII.1984, K. Morimoto (1 dissected male, KUM), JHL_AREV_849; • Mt. Funagata, Taiwa-machi, 21.VIII.2009, J. Aoki (2, KUM), JHL_AREV_839 (dissected male); **Nagano Prefecture**: • Nakabusa, Shinano, 24.VII, T. Samejima (1 dissected male, TUA), JHL_AREV_673; • Bandoko, Shinano Province, 13.VII.1935, H. Kiyosawa (4, KUM), JHL_AREV_917 (dissected male), JHL_AREV_919 – JHL_AREV_921; • Shirahone, 19.VII.1956, S. Kimoto (2, KUM), JHL_AREV_593 (dissected male), JHL_AREV_916; • Shirahone, 12.VIII.1960, Y. Kimura (1 dissected male, KUM), JHL_AREV_838; • Kamikouchi, 14.VII.1929, M. Samejima (4, TUA), JHL_AREV_783 (dissected male), JHL_AREV_967 – JHL_AREV_969; **Niigata Prefecture**: • Sasagamine, 23.VIII.1962, H. Koike (1 dissected male, KUM), JHL_AREV_896; **Tochigi Prefecture**: • Yumoto, Oku-nikko, 7.VII.1950, M. Takahashi (3, KUM), JHL_AREV_902 (dissected male), JHL_AREV_906, JHL_AREV_907; • Yumoto, 30.VII.1960, K. Suga (1 dissected male, KUM), JHL_AREV_903; • Chu-zenji, 16.VII.1916, E. Gallois (3, HUM), JHL_AREV_851; • Tokura, Nikko, 16.VI, S. Miyakawa (3, TUA), JHL_AREV_960 (dissected male); **Yamanashi Prefecture**: • Daibosatsu, 22–23.VI.1985, K. Yoshihara (6, TUA), JHL_AREV_629 (dissected male), JHL_AREV_788, JHL_AREV_789, JHL_AREV_790 – JHL_AREV_792; • Hikawa-rindo, Mts. Daibosatsu, 22.VII.1983, S. Miyakawa (21, TUA), JHL_AREV_946 (dissected male), JHL_AREV_948 – JHL_AREV_958; • Kanayama, 1.VII.1989, T. Tanaka (1 dissected male, TUA), JHL_AREV_923; • Kanayamadaira, 28–30.VI.1991, H. Urushihara (2, TUA), JHL_AREV_930 (dissected male), JHL_AREV_933; • Koshu-shi, Enzankamiigiwara, 13.VIII.2016, S. Shimamoto (2, TUA), JHL_AREV_937 (dissected male), JHL_AREV_940; • Koshu-shi, Enzankamiigiwara, 3.V.2015, S. Shimamoto (1, TUA), JHL_AREV_939; **Unknown Prefecture**: • Ichi-no-hashi, 29.VII.1975, S. Ogata (1 dissected male, KUM), JHL_AREV_502.

##### Diagnosis.

Body length 5.0–6.8 mm. Covered in black and dark gray scales, with longitudinal, black-bordered white stripes across the lateral edges of the pronotum and elytral humeri (compare with *A. cryptica* which has the entire lateral portion of the pronotum covered in white to yellow scales). Apical 1/2 of elytra with a V-shaped patch of white scales. Pronotum evenly punctured across dorsal surface, but often with shiny, impunctate longitudinal midline. Scutellum bare over anterior 1/2 of dorsal surface, otherwise covered in pale yellowish scales. Only odd elytral intervals with erect scales (even elytral intervals may possess erect scales at the extreme elytral apices). Sclerolepidia distinct and protruding. Third tarsomeres distinctly emarginate. Pedon with lateral edges approx. parallel in dorsal view, and converging evenly at apex into a dull, rounded point. Internal sac visible in anterior 1/2 of aedeagus and tuberculate (roughed appearance); • internal sac also with hook-like protruding structure anteriorly (at the apex) and surrounded by tuberculate, roughened musculature (Fig. 6A1–2).

##### Distribution.

This species occurs in far Eastern Russia (Primorsky Krai, Saghalien) and north-central mainland Japan (from Hokkaido south to Gifu Prefecture).

##### Remarks.

The two *A. albofasciata* syntypes deposited in the ZIN Collection are apparently both females, despite the original paper describing a male (B. Korotyaev to JHL, pers. comm. 2025). Dissection of males is required to conclusively differentiate between *A. albofasciata* and its sister species *A. cryptica*. However, based on our extensive collection examinations, we found that *Acicnemis albofasciata* occurs in the Far East (Primorsky Krai, Russia), Hokkaido, and northern mainland Japan, while *A. cryptica* occurs only in mainland Japan, from Aomori Prefecture south to Kyushu (Oita Prefecture). Furthermore, examined specimens of *A. albofasciata* have a black-scaled, lateral stripe on the pronotum, while *A. cryptica* have the lateral portion of the pronotum mostly covered in yellow to white scales. We are confident that the Ter-Minasian syntypes belong to *A. albofasciata* (not *A. cryptica*) as only this species apparently occurs outside of Japan (with known records from the syntype locality (Primorsky Krai, Russia), and as the Ter-Minasian syntypes have a black-scaled lateral pronotum band. To fix the identity of *A. albofasciata*, we hereby designate one of the Ter-Minasian syntypes (JHL_AREV_997) as a lectotype.

#### ﻿ Acicnemis azumai

Taxon classificationAnimaliaColeopteraCurculionidae

Morimoto & Miyakawa, 1995

8BBE2064-DEF1-5BE5-BFE5-6BF7AC8123C5

Figs 1, 2A, 3A, 6B1–6, 2, 7

##### Type material examined

**(Japan: 82; South Korea: 3; Total: 84). *Holotype*. Japan: Okinawa Prefecture**: • Kudeken, Chinen-son, 22.IV.1975, H. Irie, bears red label reading “(HOLOTYPE) Acicnemis azumai Morimoto et. Miyakawa, 1994”, JHL_AREV_113, ELKU, ELKU 2962. ***Paratypes*. Japan: Okinawa Prefecture**: • Sueyoshi, “1988-6-5”, T. Ueno (5, KUM), JHL_ACITAI_041 (dissected male), JHL_AREV_134 – JHL_AREV_137; • Yona, 9–13.VIII.1969, H. Makihara (4, KUM), JHL_AREV_108 – JHL_AREV_111; • Shuri, 14.VI.1958, O. Nakachi (1, KUM), JHL_AREV_138; • Shuri, 16–20.V.1978, H. Makihara (1, KUM), JHL_AREV_117; • Aguni Island, 24–25.VIII.1989, Teruhisa Ueno, JHL_ACITAI_081 (dissected male) (4, KUM), JHL_AREV_124 – JHL_AREV_126; • Mabuni, 11.VI.1977, H. Irie (2, KUM) JHL_AREV_127, JHL_AREV_128; • Nago, 18.VI.1984, Teiso Esaki (1, KUM), JHL_AREV_130; • Akajima Island, 29–30.VIII.1980, Teruhisa Ueno (1, KUM), JHL_AREV_129; • Mount Banna, Ishigaki Island, 30.VII.1970, I. Matoba (1, KUM), JHL_AREV_131; • Tonaki Island, 27.IX.1989, Teruhisa Ueno (2, KUM), JHL_AREV_132, JHL_AREV_133; • 18.VI.1970, H. Makihara (2, KUM), JHL_AREV_122, JHL_AREV_123; • Tamagusuku, 4.V.1962, M. Kina (1, KUM), JHL_AREV_151; • Tamagusuku, 23.VIII.1962, M. Kina (2, KUM), JHL_AREV_152, JHL_AREV_153; • Otomi, Iriomote Island, 25.IV.1969, H. Makihara (1, KUM), JHL_AREV_143; • Shimozato, Miyako Island, 1.IX.1958, T. Hidaka (1, KUM), JHL_AREV_144; • Kudeken, 31.V.1976, T. Ogasawara (1, KUM), JHL_AREV_145; • Shuri, 5–9.V.1969, H. Makihara (1, KUM), JHL_AREV_146; • Naha, 13.VIII.1952, K. Hamamatsu (1, KUM), JHL_AREV_147; • Sueyoshi, 9.VII.1987, Teruhisa Ueno (1, KUM), JHL_AREV_148.

**Figure 7.**
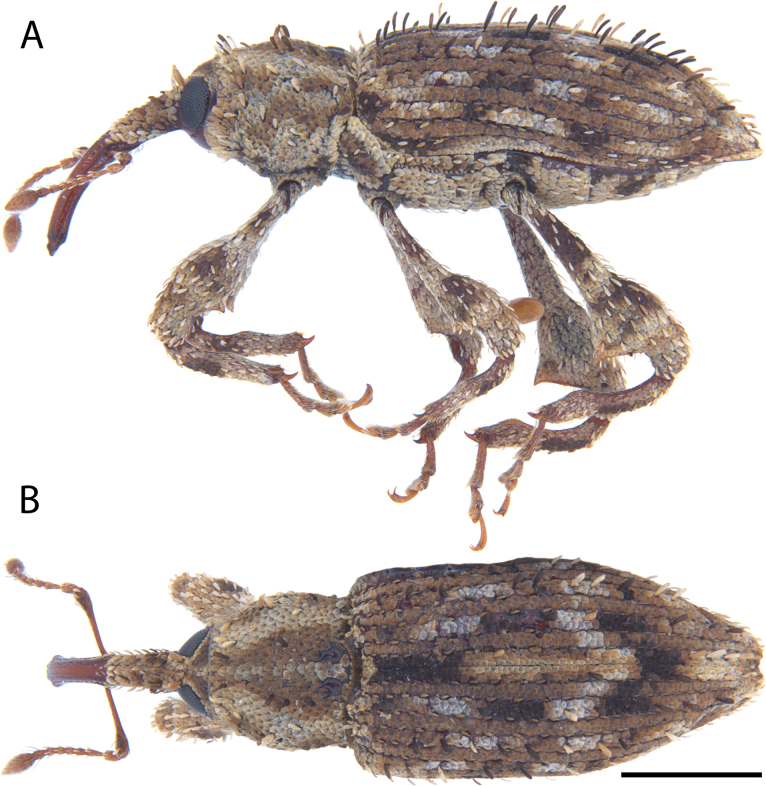
*Acicnemis azumai* Morimoto & Miyakawa, 1995 (OKENT0132427). A. Lateral; B. Dorsal. Scale bar: 1 mm.

##### Non-type material.

**Japan: Okinawa Prefecture**: • Iriomote Island, Mt. Tedo, 28.III.2022, Y. Obae (1, KUM), JHL_AREV_511; • Ishigaki Island, Inoda, 24.III.2022, S. Imada, M. Maruyama, T. Nozaki (1, KUM), JHL_AREV_512; • Kabira Park, Ishigaki Island, 6.VI.1968, M. Takagi (1, KUM), JHL_AREV_763; • Okinawa Island, Sakukawa, 8.XI.1971, T. Terya (1, RUMC), JHL_AREV_513; • Okinawa Island, Nishihara, 16.VII.1977, M. Arasaki (1, RUMC), JHL_AREV_514; • Okinawa Island, Yona, 24.IV.1965, T. Kakinohana (2, RUMC), JHL_AREV_515, JHL_AREV_516; • Okinawa Island, Kudeken, 28.VI.1977, K. Omato (1, RUMC), JHL_AREV_517; • Okinawa Island, Shuri, 7.VII.1972, M. Kinjo (1, RUMC), JHL_AREV_518; • Miyako Island, 1.IX.1958, T. Takara (3, RUMC), JHL_AREV_519 – JHL_AREV_521; • Okinawa Island, Shuri, 16.VI.1961, O. Nakachi (1, RUMC), JHL_AREV_522; • Okinawa Island, Shuri, 24.VIII.1962, O. Nakachi (1, RUMC), JHL_AREV_523; • Okinawa Island (1, OIST), JHL_AREV_984 (GenBank accession number: PV255625); • Tancha, Okinawa Island, 4.III.2025, J.H. Lewis, to residential lights (1, OIST), JHL_AREV_995; • Tancha, Okinawa Island, 14.III.2025, J.H. Lewis, to residential lights (1, OIST), JHL_AREV_993; • Tancha, Okinawa Island, 29.VIII.2025, J.H. Lewis, to residential lights (1, OIST), JHL_AREV_2081; • Sueyoshi Park, Naha, 12–26.II.2016, (1, OIST), OKENT0132427; **South Korea** • Oseag Ri, Ganweondo, 4–6.VII.1984, T. Senoh (1, KUM), JHL_AREV_155; • Sam Jeong Li, Ma Cheong Meon, Hamyang Gun, Gyeongsamnamdo, 9–15.V.1991, K. Morimoto (2, KUM), JHL_AREV_154, JHL_ACITAI_079 (dissected male).

##### Diagnosis.

Body length 3.3–4.9 mm. Covered in pale brown scales, with alternating white and black scaled bands on odd elytral intervals. Pronotum with two exposed, bare tubercles medially at the base (one on each side) (Fig. 3A). Scutellum covered in yellow scales. Only odd elytral intervals with erect scales. Sclerolepidia distinct and protruding. Posterior edge of prosternum lacking prominent projections contiguous with the fore-coxae (Fig. 2A) (present in *A. laeta*). Third tarsomeres distinctly emarginate. Pedon with lateral edges approx. parallel in dorsal view, but converging evenly into a point. Internal sac without tuberculate or roughened musculature inside the aedeagus, but with a non-hooked protruding structure (unmodified in posterior 1/2, with roughened in anterior 1/2) that is equal in length to the pedon (Fig. 6B1–2).

##### Distribution.

This species occurs in southern Japan (Okinawa Prefecture, Ryukyu Islands) and South Korea.

##### Remarks.

On Okinawa Island (Japan) this species is commonly encountered in the spring and is often attracted to residential building lights.

#### ﻿ Acicnemis biarcuata

Taxon classificationAnimaliaColeopteraCurculionidae

Hubenthal, 1919

BAB38C43-B84D-5AD8-A739-F31F12F84438

Fig. 8

##### Type material examined

**(Taiwan: 5). *Holotype*. Taiwan** • “Kosempo”, “Formosa”, “IX.09”, “Sauter”, bears red label reading “Holotypus”, SDEI, SDEIColeoptera #303414, SDEI Muncheburg COL – 18569.

**Figure 8.**
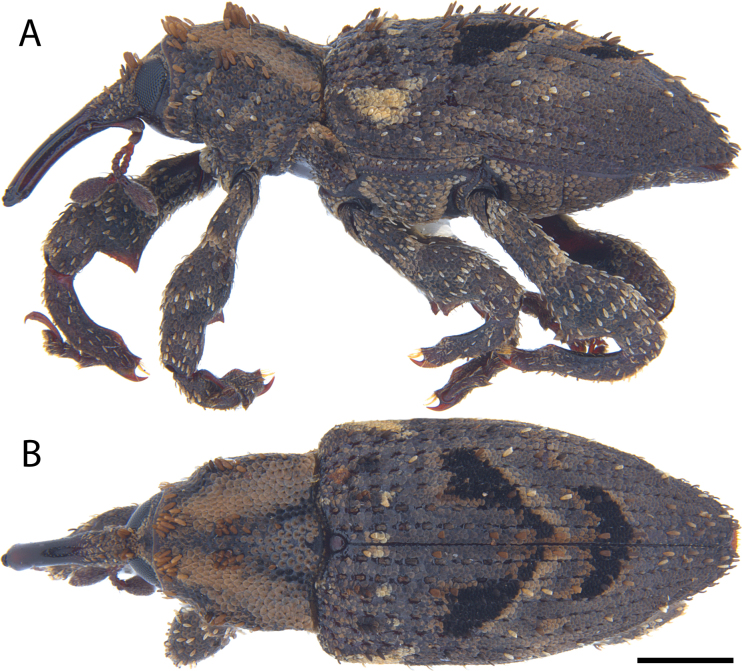
*Acicnemis biarcuata* Hubenthal, 1919 (JHL_AREV_628). A. Lateral; B. Dorsal. Scale bar: 1 mm.

##### Non-type material.

**Taiwan** • Lienhoachii, “1975.5.12”, S. Imasaka (1, TUA), JHL_SYN_184; • Mudan Township, Pingtung County, 22°08'21.71"N, 120°51'31.23"E, 9.III.2013, Y. Fujisawa (1, TUA), JHL_AREV_626; • Mudan Township, Pingtung County, 22°06'12.57"N, 120°47'33.71"E, 6.III.2013, Y. Fujisawa (1, TUA), JHL_AREV_627; • Manzhou Township, Pingtung County, 22°08'30"N, 120°51'13"E, 2.V.2019, S. Shimamoto (1, TUA), JHL_AREV_628.

##### Diagnosis.

Body length 4.0–5.2 mm. Covered in dark-gray scales, with two V-shaped, transverse black bands across middle of elytra. Pronotum with pale brown and yellow scales dorsally, but dark at base medially. Pronotum approx. sculptured and carinate. Scutellum shiny and bare in examined specimens. Only odd elytral intervals with erect scales across elytral disk (some erect scales on 2^nd^ interval in apical fourth of elytra). Third and fifth elytral intervals with cluster of 3–5 erect scales at elytral base. Sclerolepidia distinct and protruding. Mesosternum with distinctly raised ridge between meso-coxae, highest laterally but dipping shallowly medially (no such ridge or only weakly developed in most other species). Third tarsomeres weakly emarginate.

##### Distribution.

This species is currently only known from Taiwan.

#### ﻿ Acicnemis bickhardti

Taxon classificationAnimaliaColeopteraCurculionidae

Hubenthal, 1919

A2A84396-C691-58FD-A08B-252BE9BD89DC

Fig. 9

##### Type material examined

**(Taiwan: 3). *Holotype*. Taiwan** • Kosempo, H. Sauter, 1909, bears a red label reading “Type”, SNSD, JHL_ACITAI_001.

**Figure 9.**
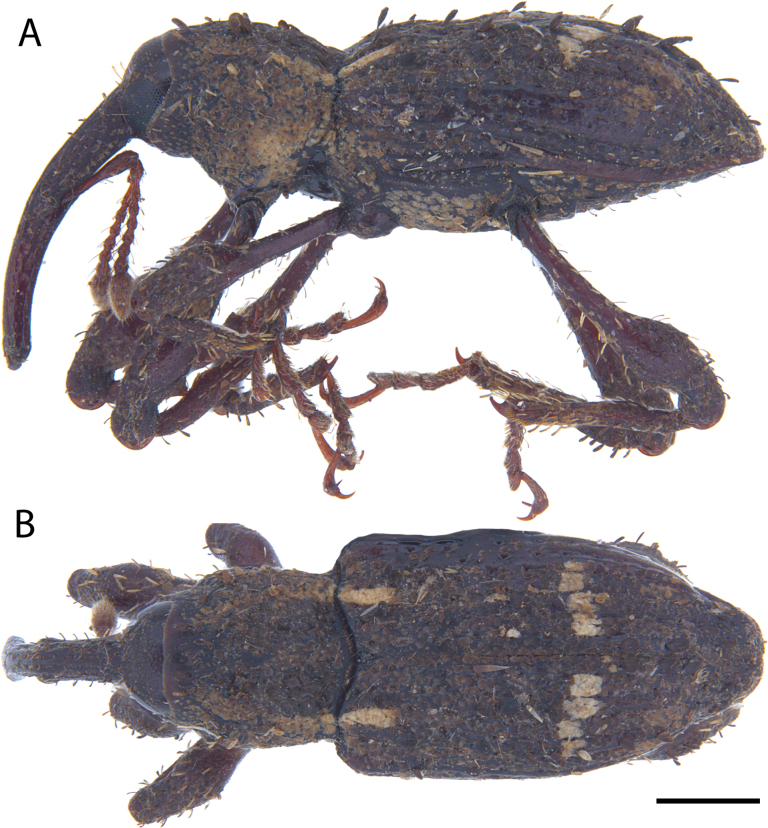
*Acicnemis bickhardti* Hubenthal, 1919 (JHL_AREV_591). A. Lateral; B. Dorsal. Scale bar: 1 mm.

##### Non-type material.

**Taiwan** • “16.VII.20”, T. Kano (1, NMNS), JHL_AREV_591; • 20.VI.1938, unknown collector (1, TUA), JHL_AREV_592.

##### Diagnosis.

Body length 5.0–7.0 mm. Easily distinguished from other East Asian *Acicnemis* by the (1) large size (2) black to dark red cuticle largely naked, but with bright yellow scaled bands running longitudinally across the lateral edges of the pronotum and elytral humeri, as well as transversally across the apical 1/2 of the elytra.

##### Distribution.

This species is known only from Taiwan.

##### Remarks.

Many similarly patterned, large species that presumably form a distinct clade within *Acicnemis* are present throughout Southeast Asia (e.g., *A. ibis* Faust, 1896 and *A. peduncularis* Pascoe, 1872), and a number of these remain undescribed.

#### ﻿ Acicnemis cordata

Taxon classificationAnimaliaColeopteraCurculionidae

Hubenthal, 1919

2C364F23-F2EB-5514-8197-EDD143888B42

Fig. 10

Acicnemis fukienensis Voss, 1958.

##### Type material examined

**(Vietnam: 1). *Holotype*. Vietnam** • “Mau-son, Tongking” (modern day northern Vietnam), “Gehr. W. Muller, Vermacht, 1909”, bears a red label reading “Typus”, SNSD, JHL_AREV_987.

**Figure 10.**
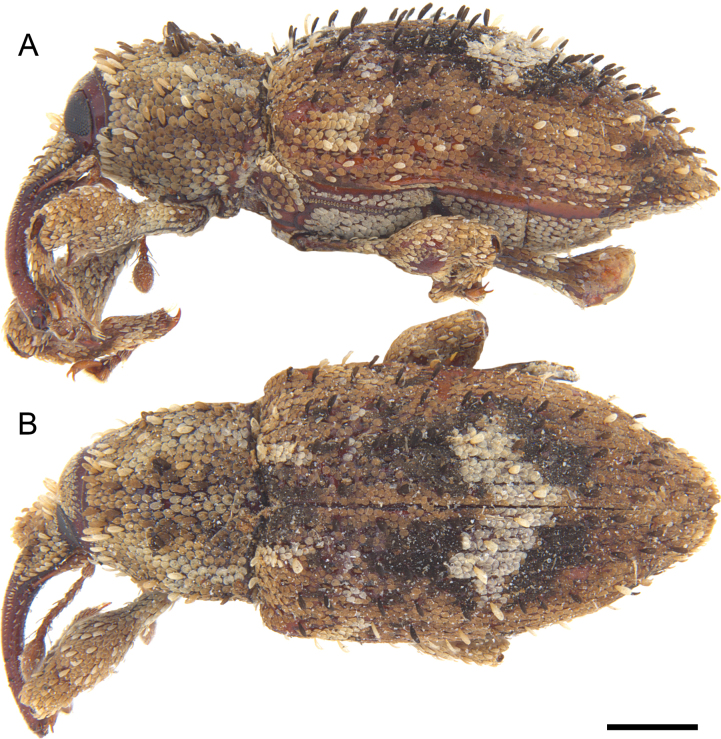
*Acicnemis cordata* Hubenthal, 1919 holotype (JHL_AREV_987). A. Lateral; B. Dorsal. Scale bar: 1 mm.

##### Diagnosis.

Body length 5.0 mm. Virtually indistinguishable externally from *A. maculaalba* (see that species). Although most *A. maculaalba* have the white elytral spots interrupted by brown scales along the first elytral interval, some have no interruption (as in the *A. cordata* holotype); therefore, this character cannot be used alone to reliably separate *A. maculaalba* and *A. cordata*.

##### Distribution.

This species is known from the type locality (Mau-son, Tongking, which corresponds to modern day northern Vietnam) and apparently also Kuatun, China (Voss’s specimen).

##### Remarks.

We examined the holotype specimen of *A. cordata* Hubenthal, 1919 (collected from Fujian, China; SNSD) and found it is virtually indistinguishable externally from Taiwanese and Japanese specimens of *A. maculaalba*. However, as the *A. cordata* holotype is a female, it was not possible to examine the male genitalia, which are extremely important for distinguishing members of the *A. maculaalba* species complex. We tentatively recognize *A. cordata* here, but also note that further examination of specimens from mainland Asia is necessary to work out the species in this complex.

#### ﻿ Acicnemis costulifera

Taxon classificationAnimaliaColeopteraCurculionidae

Hubenthal, 1919

F2EFD8DB-464C-5BB4-9665-B1AE70E0D11E

Fig. 11

##### Type material examined

**(Unknown country: 1). *Holotype*. Unknown locality**: “Coll. J. Faust Ankauf 1900”, bears a red label reading “Typus”, SNSD, JHL_ACITAI_003.

**Figure 11.**
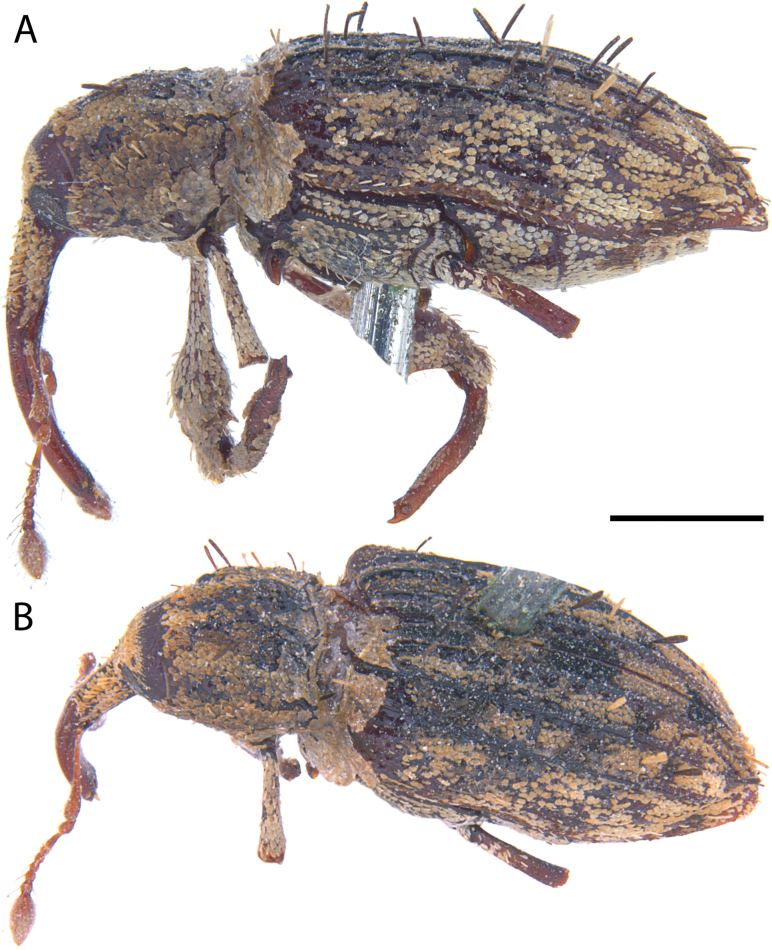
*Acicnemis costulifera* Hubenthal, 1919 (JHL_ACITAI_003). A. Lateral; B. Oblique. Scale bar: 1 mm (applies only to A). Note that the silver region is an entomological pin.

##### Diagnosis.

Body length 4.8 mm. This is the only species covered here with costate elytra (on the 2^nd^, 4^th^, and 6^th^ intervals). Also, is has erect, elongate scales along the odd-numbered elytral intervals (as in *A. ryukyuana*).

##### Distribution.

*Acicnemis costulifera* is recorded from Taiwan and Japan in Alonso-Zarazaga et al. (2023); however, Hubenthal (1919b) states that the holotype collection locality is unknown. We examined the holotype specimen and indeed, it does lack labels with any locality data. Furthermore, no specimens of this species collected from Japan were encountered in any of the examined collections. As such, we are confident that the record of this species from Japan is erroneous, and that the record from Taiwan should be treated with suspicion until additional specimens are found.

#### ﻿ Acicnemis cryptica

Taxon classificationAnimaliaColeopteraCurculionidae

Lewis & Kojima sp. nov.

183AA775-DE6B-51CB-8245-8DC267CF10C2

https://zoobank.org/D117E957-E5E4-47B0-BF8A-4444EC767456

Figs 6C1–6, 2, 12

##### Type material

**(Japan: 72). *Holotype*. Japan: Nagano Prefecture**: • Otari Onsen, Otari-mura, 23.VII.1990, M. Hasegawa, bears a red holotype label reading “HOLOTYPE / *Acicnemis cryptica* / Lewis & Kojima, 2025”, JHL_AREV_672, dissected male, deposited in KUM. ***Paratypes*. Japan: Aomori Prefecture**: • Towada, 14.VI.1957, K. Shimoyama (1 dissected male, KUM), JHL_AREV_498; **Fukushima Prefecture**: • Yunohana, Tateiwa V., Minami-Aizu, 14.VI.1947, “Y.K.” (1 dissected male, NMNS), JHL_AREV_139; **Fukuoka Prefecture**: • Hikosan (Buzen), 19.V.1939, H. Hori (1 dissected male, KUM), JHL_AREV_981; **Gunma Prefecture**: • Mt. Buson, 13–15.VII.1975, H. Irie (2, KUM), JHL_AREV_904 (dissected male), JHL_AREV_905; **Kochi Prefecture**: • Mt. Tebako, 7–10.VIII.1957, K. Morimoto (13, KUM), JHL_AREV_479 (dissected male), JHL_AREV_823 – JHL_AREV_827, JHL_AREV_829 – JHL_AREV_831, JHL_AREV_833 – JHL_AREV_835, JHL_AREV_837; • Mt. Tebako, 18–21.VII.1955, K. Kojima (2, KUM), JHL_AREV_828, JHL_AREV_832; • Monobe-mura, 26–28.VIII.1959, K. Morimoto (2, KUM), JHL_AREV_836, JHL_AREV_845; **Ibaraki Prefecture**: • Mt. Yamizo-san, Daigo-machi, 1.XII.1995, S. Nomura (1 dissected male, KUM), JHL_AREV_901; **Iwate Prefecture**: • Mt. Hayachine, 2–5.VIII.1982, H. Makihara (8, KUM), JHL_AREV_594 (dissected male), JHL_AREV_817, JHL_AREV_818, JHL_AREV_822, JHL_AREV_841 – JHL_AREV_844; **Nagano Prefecture**: • Otari Onsen, Otari-mura, 23.VII.1990, M. Hasegawa (1, TUA), JHL_AREV_819; **Oita Prefecture**: • Mt. Sobo, 29.VII.1953, T. Matsuda (1 dissected male, KUM), JHL_AREV_544; **Shizuoka Prefecture**: • Amagi-toge, Hacchoike Izu-hanto, 14.V.1980, J. Okuma (4, TUA), JHL_AREV_947 (dissected male), JHL_AREV_959; • **Tokyo Metropolis**: Nippara, 19.IX,1971, H. Takizawa, JHL_AREV_118 (1, HUM); • Ogawadani, Okutama, “1966.8.6”, T. Goh (1 dissected male, HUM), JHL_AREV_140; • Mt. Takao, 27.V.1917, E. Gallois (3, HUM) JHL_AREV_114 (dissected male), JHL_AREV_141, JHL_AREV_142; • Mt. Takao, 10.V.1914, E. Gallois (1, HUM), JHL_AREV_115; • Mt. Takao, Hachioji, 22.VIII.2024, 35.62972°N, 139.2525°E, R. Anderson, beating in mixed maple / beech / cypress forest, “2024-102” (2, CMNC), JHL_AREV_2035, JHL_AREV_2026; • Takao Musashi, V.1912, H. Takabayashi (1, HUM), JHL_AREV_116; **Yamanashi Prefecture**: • Daibosatsu, 19.V.1974, M. Tao (1, TUA), JHL_AREV_778; • Koganezawa-rindo, Ohtsuki-shi, 30–31.V.1980, J. Okuma (5, TUA), JHL_AREV_630 (dissected male), JHL_AREV_820, JHL_AREV_821; • Koganezawa-rindo, 23.VI.1974, S. Miyakawa (8, TUA), JHL_AREV_941 – JHL_AREV_945.

**Figure 12.**
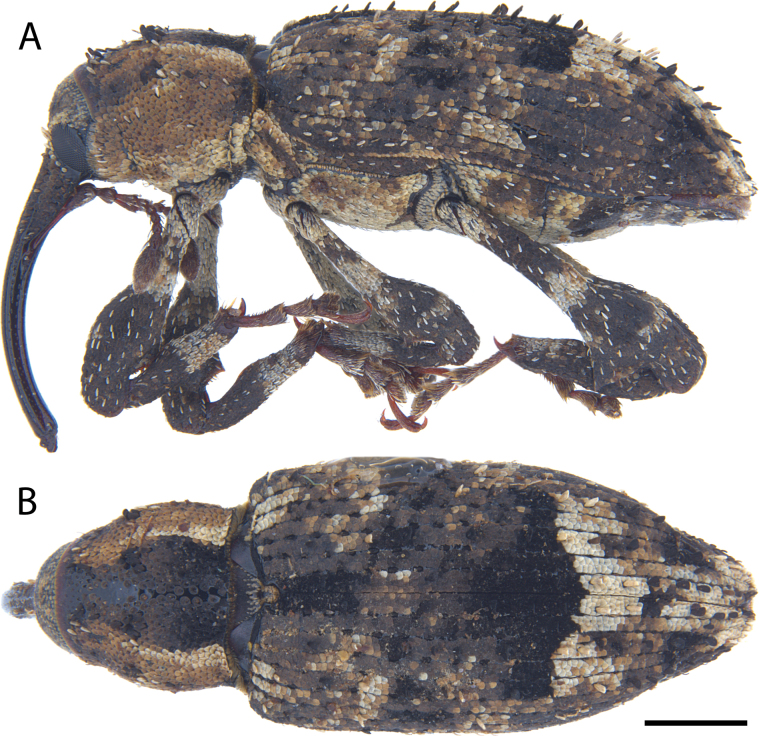
*Acicnemis cryptica* Lewis & Kojima, sp. nov. (JHL_AREV_778). A. Lateral; B. Dorsal. Scale bar: 1 mm.

##### Diagnosis.

Body length 4.9–7.0 mm. Covered in dark to pale gray and black scales. Pronotum covered in black scales medially, but with white to yellow scales laterally (compare with *A. albofasciata* which also has white lateral pronotal bands, but bordered by black scales at the lateral-most extreme of the pronotum). Apical 1/2 of elytra with a V-shaped patch of white scales. Pronotum evenly punctured across dorsal surface, but often with shiny, impunctate longitudinal midline. Scutellum bare over anterior 1/2 of dorsal surface, otherwise covered in pale yellowish scales. Only odd elytral intervals with erect scales (even elytral intervals may possess erect scales at the extreme elytral apex). Sclerolepidia distinct and protruding. Third tarsomeres distinctly emarginate. Pedon with lateral edges approx. parallel in dorsal view, but sinuating into an acute point (Fig. 6C1–2). Internal sac without tuberculate or roughened musculature inside the aedeagus (compare with *A. albofasciata*, which has a tuberculate internal sac visible inside the aedeagus), but with hook-shaped protruding structure surrounded by tuberculate, roughened musculature.

##### Distribution.

This species is endemic to mainland Japan (excluding Hokkaido), north from Aomori Prefecture and south to Kyushu (Oita Prefecture, Fukuoka Prefecture).

##### Etymology.

The specific name *cryptica* is a reference to the remarkable similarity of this species in external morphology to *A. albofasciata* and also explains why it was overlooked for decades. We also suggest the Japanese common name ハイイロカレキゾウムシ [Haiiro-kareki-zoumushi], which translates in English to “Gray-dead-wood-weevil”.

##### Remarks.

This cryptic species was previously assumed to be a pale-color morph of *A. albofasciata* (see Morimoto and Miyakawa 1995: 50, fig. 11), and indeed only differs externally from *A. albofasciata* in overall color (pale gray in *A. cryptica*; black to dark gray in *A. albofasciata*) and weakly in scale pattern (entire lateral portion of pronotum white to yellow scaled in *A. cryptica*; isolated white to yellow band, bordered by black lateral pronotal band in *A. albofasciata*). The male genitalia of *A. cryptica* (which were apparently not examined previously), however, are completely distinct from *A. albofasciata* and differ in the shape of the aedeagus as well as in internal sac morphology (Fig. 6C1–2).

#### ﻿ Acicnemis dividicincta dividicincta

Taxon classificationAnimaliaColeopteraCurculionidae

Morimoto & Miyakawa, 1995

0B173776-B892-5A96-AF78-7090D19F92E6

Figs 6D1–6, 2, 13

Acicnemis yakushimana Morimoto & Miyakawa, 1995, syn. nov.

##### Type material examined

(Japan: 34). ***Holotype*. Japan: Okayama Prefecture**: • Mt. Gagyuzan, Takahashi City, 19–20.V.1975, H. Irie, bears red label reading “(HOLOTYPE) Acicnemisdividicincta Morimoto et. Miyakawa, 1994”, JHL_SYN_120, ELKU, ELKU 2957. ***Paratypes*. Fukuoka Prefecture**: • Mt. Hiko, 5.IX.1988, K. Morimoto (1, KUM), JHL_SYN_118; **Kochi Prefecture**: • Mt. Tebako, 7–10.VIII.1957, K. Morimoto (2, KUM), JHL_SYN_114, JHL_SYN_115; **Nagasaki Prefecture**: • Unzen, 18.VII.1946, M. Moritsu & Y. Kurosawa (3, KUM), JHL_SYN_102 (dissected male), JHL_SYN_113, JHL_SYN_119; **Okayama Prefecture**: • Mt. Gagyuzan, Takahashi City, 19–20.V.1975, H. Irie (3, KUM), JHL_SYN_111, JHL_SYN_112, JHL_DNA_142 (dissected male); **Wakayama Prefecture**: • Mt. Ootoh, 7.VIII.1980, I. Matoba (1, KUM), JHL_SYN_116.

**Figure 13.**
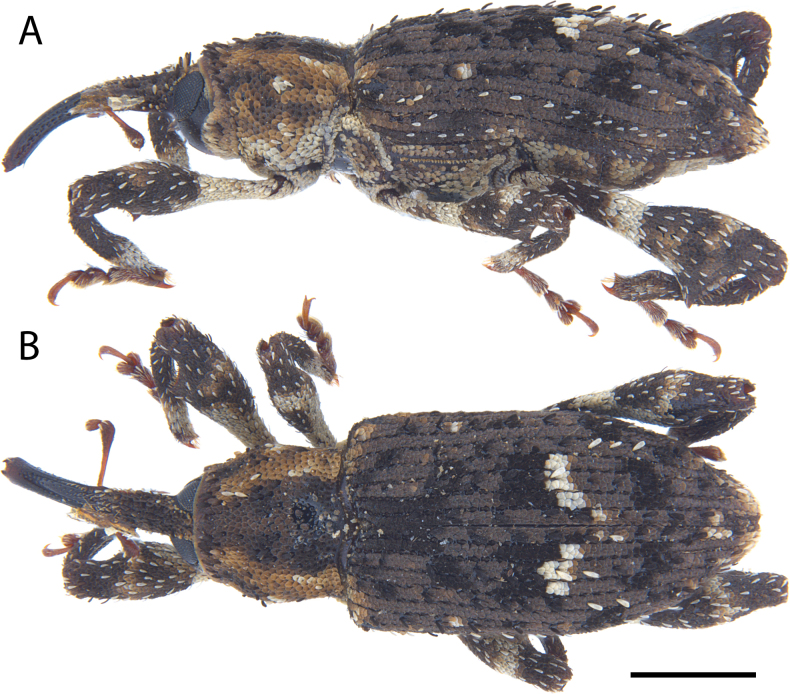
*Acicnemis dividicincta dividicincta* Morimoto & Miyakawa, 1995 (JHL_AREV_772). A. Lateral; B. Dorsal. Scale bar: 1 mm.

##### Other type material.

**Kagoshima Prefecture**: • Miyanoura, Yakushima Island, 26.VII.1974, T. Mikage, bears red label reading “(HOLOTYPE) Acicnemis yakushimana Morimoto et. Miyakawa, 1994”, JHL_SYN_180 (1, ELKU), *A. yakushimana* holotype specimen, ELKU 2959; • Miyanoura, Yakushima Island, 26–29.VII.1974, T. Mikage (13, KUM), JHL_SYN_122 – JHL_SYN_124, JHL_SYN_127 – JHL_SYN_130, JHL_SYN_132, JHL_SYN_133, JHL_SYN_138, JHL_SYN_139, JHL_DNA_141 (dissected male); • Shiratani, Yakushima Island, 24–26.VII.1974, T. Mikage (2, KUM), JHL_SYN_121, JHL_SYN_131; • Mt. Tachudake, Yakushima Island, 20.VII.1971, H. Irie (4, KUM), JHL_SYN_125, JHL_SYN_126, JHL_SYN_134, JHL_SYN_135; • Ambo – Kosugidani, Yakushima Island, 29.VII.1929, Hiroshi Hori (2, KUM), JHL_SYN_136, JHL_SYN_137; • Shiratani-rindo, Yakushima Island, 20.VII.1976, M. Kaneda (1, KUM), JHL_AREV_663.

##### Non-type material.

**Ehime Prefecture**: • Omogo, Iyu, 19.VIII.1969, T. Nakane, (1, HUM), JHL_AREV_772; • **Tokyo Metropolis**: Hachijo-jima, Mitsune, VI.2001, T. Kinoshita (1 dissected male, KPMNH), JHL_AREV_936; • Hachijo-jima, Noboryou-toge, 9.VI.1996, I. Hirai (1, KUM), JHL_AREV_938.

##### Diagnosis.

Body length 4.2–7.0 mm. Covered in black and dark gray scales, with two V-shaped, transverse white and black bands across middle of elytra. Pronotum with weakly patterned gray, black and faint yellow scales. Scutellum covered loosely in yellow scales (denuded in many specimens). Only odd elytral intervals with erect scales across elytral disk (some erect scales on 2^nd^ interval in apical fourth of elytra). Sclerolepidia distinct and protruding. Third tarsomeres distinctly emarginate. Pedon with lateral edges approx. parallel in dorsal view, but converging evenly into a moderately acute point. Internal sac not visible inside aedeagus, but with a non-hooked protruding structure (unmodified in posterior 1/2, with roughened in anterior 1/2) that is clearly longer than pedon (Fig. 6D1–2).

##### Distribution.

This subspecies occurs in mainland Japan (central Japan south to Yakushima Island) and adjunctly on Hachijo Island (Tokyo Metropolis).

##### Remarks.

*Acicnemis yakushimana* was distinguished from *A. dividicincta* by Morimoto and Miyakawa (1995) on the basis of purported differences in the relative proportions of the funicular antennomeres and minor differences in color. Furthermore, *A. yakushimana* also reportedly possessed a sclerite in the internal sac at the gonopore. After dissecting several specimens of each species across their geographic range, we did not observe any differences in genital morphology between *A. yakushimana* and *A. dividicincta*. Furthermore, no observable differences in antennal funicle proportions were observed. As such, we hereby treat *A. yakushimana* syn. nov. as a junior subjective synonym of *A. dividicincta*.

#### ﻿ Acicnemis dividicincta okinawana

Taxon classificationAnimaliaColeopteraCurculionidae

Morimoto & Miyakawa, 1995

2923444F-AAB0-5781-AB11-91A0E92145B5

Figs 1, 14

##### Type material examined

**(Japan: 13). *Holotype*. Japan: Okinawa Prefecture**: • Hiji, Kunigami-son, Okinawa Island, 8.VII.1974, T. Mikage, bears red label reading “(HOLOTYPE) Acicnemis dividicincta okinawana Morimoto et. Miyakawa, 1995”, JHL_SYN_110, ELKU, ELKU 2958. ***Paratypes*. Japan: Kagoshima Prefecture**: • Torigamine, Amami Island, 30.VI.1976, N. Morishimo (1, KUM), JHL_SYN_104; **Okinawa Prefecture**: • Hiji, Kunigami-son, Okinawa Island, 3–9.VII.1974, T. Mikage (5, KUM), JHL_SYN_101 (dissected male), JHL_SYN_103, JHL_SYN_105 – JHL_SYN_107.

**Figure 14.**
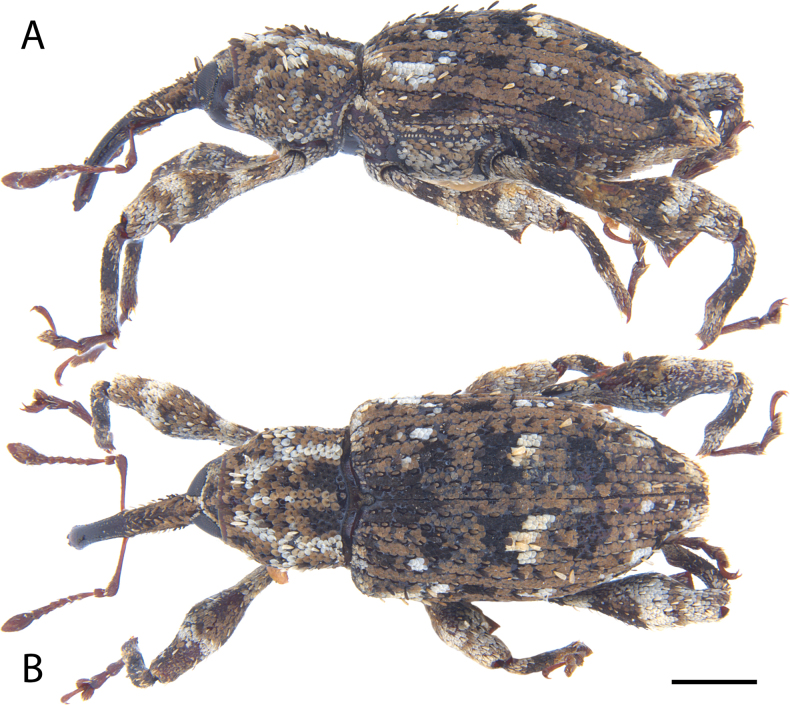
*Acicnemis dividicincta okinawana* Morimoto & Miyakawa, 1995 (JHL_SYN_106). A. Lateral; B. Dorsal. Scale bar: 1 mm.

##### Non-type material.

**Japan: Kagoshima Prefecture**: • Mt. Yuwandake, Amami-Oshima, 5–9.VII.1968, K. Sakai (1, CMIC), CBM-ZI 68475; **Okinawa Prefecture**: • Tancha, 1–7.VI.2022, J.H. Lewis (1, OIST), JHL_DNA_126 (GenBank accession number: PV255626); • Tancha, Onna-son, Okinawa Island, 2.VI.2022, sweeping coastal vegetation, J.H. Lewis (1, OIST), JHL_SYN_109; • Yona, Okinawa Island, 19.X.1973, S. Azuma (1, RUMC), JHL_SYN_108; • Tancha, Onna-son, Okinawa Island, 29.VIII.2025, J.H. Lewis, to residential lights (1, OIST), JHL_AREV_2082; • Oku, Okinawa Island, 26.83604°N, 128.27191°E, 5–19.VIII.2016 (1, OIST), OKENT0089525 (GenBank accession number: PV255627).

##### Diagnosis.

Body length 4.8–6.5 mm. Identical to *A. dividicincta dividicincta* above, differing from that species only in possessing somewhat paler gray scales.

##### Distribution.

This subspecies occurs on Amami-Oshima (Kagoshima Prefecture) and Okinawa Island (Okinawa Prefecture).

##### Remarks.

We continue to recognize this subspecies as it is significantly isolated from the mainland populations and therefore presumably does represent a distinct intraspecific lineage.

#### ﻿ Acicnemis exilis

Taxon classificationAnimaliaColeopteraCurculionidae

Morimoto & Miyakawa, 1995

A9AD03D2-B69C-5CFD-A239-305F33D36AA1

Figs 1, 6E1–6, 2, 15

##### Type material examined

**(Japan: 21; Taiwan: 3; Total: 24). *Holotype*. Japan: Kagoshima Prefecture**: • Amami-Oshima, Naze, 27.V.1978, T. Tsutsumi, bears red label reading “(HOLOTYPE) Acicnemis exilis Morimoto et. Miyakawa, 1994”, ELKU, ELKU 2963.

**Figure 15.**
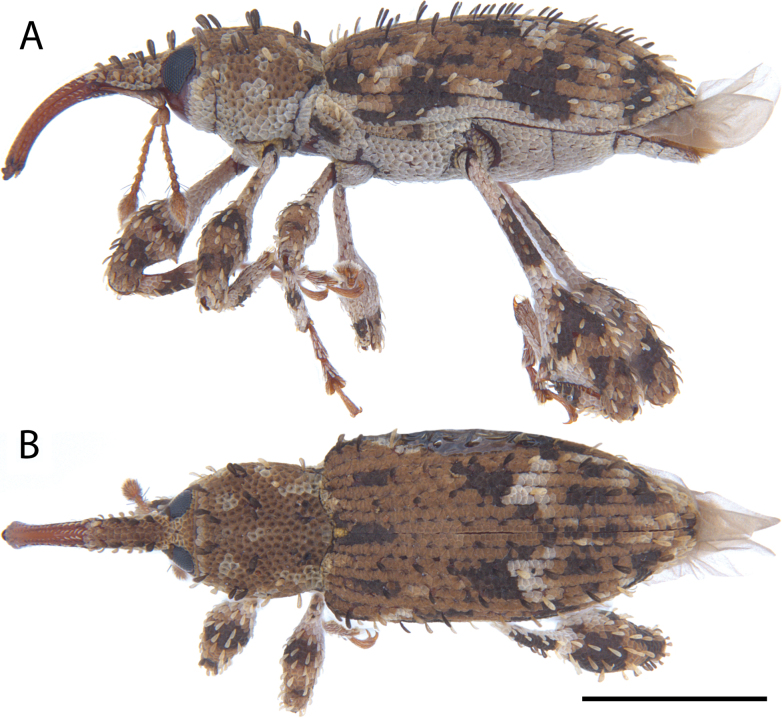
*Acicnemis exilis* Morimoto & Miyakawa, 1995 (OKENT0065625). A. Lateral; B. Dorsal. Scale bar: 1 mm.

##### Non-type material.

**Japan: Kagoshima Prefecture**: • Jinushi-daimyojin, Satomura, Nakano-jima Island, 7–9.VII.2019, S. Imada (2, KUM), JHL_AREV_595, JHL_AREV_596; **Okinawa Prefecture**: • Hikawa, Yonaguni Island, 3.V.2016, N. Tsuji (1, KUM), JHL_AREV_597; • Banna-koen, Ishigaki Island, 1.IX.2015, J. Aoki (9, KUM), JHL_SYN_145 (dissected male), JHL_AREV_598, JHL_AREV_599, JHL_AREV_601 – JHL_AREV_605; • JHL_AREV_724; • Mt. Omoto-dake, 15–17.IV.2014, S. Yamamoto (2, KUM), JHL_AREV_606, JHL_AREV_607; • Southeast Botanical Gardens, Okinawa Island, 10–24.II.2016 (1 dissected male, OIST), OKENT0109018; • Chatan, Ireibaru, 26.32468°N, 127.75918°E, 11–25.XI.2015 (1, OIST), OKENT0105900 (GenBank accession number: PV255629); • Nanjo, Tamagusuku, 26.32468°N, 127.75918°E, 9–23.IX.2015, OKENT0056923 (GenBank accession number: PV255628) (1, OIST); • Sefa-utaki, Nanjo, 15–29.VII.2016, (1, OIST), OKENT0065625; near Hiji, Okinawa Island, 16.III.2024, J.H. Lewis (1, OIST), JHL_AREV_998; • Banna-dake, Ishigaki Island, 20.VII.1973, H. Takizawa (1, HUM), JHL_ACITAI_086; **Taiwan** • Wulai (near Taipei), 27.V.1965, K. Morimoto (1 dissected male, KUM), JHL_AREV_624; • Kotosho Island, 13–15.VIII.1968, H. Makihara (2, KUM), JHL_AREV_757, JHL_AREV_758.

##### Diagnosis.

Body length 2.1–3.0 mm. Covered in pale brown, white, and gray scales, with two white-scaled spots in the apical 1/2 of the elytra. Scutellum covered in yellow scales. Only odd elytral intervals with erect scales. Sclerolepidia small but protruding. Third tarsomeres distinctly emarginate. Pedon with lateral edges approx. parallel in dorsal view, but converging evenly into a rounded point. Internal sac without tuberculate or roughened musculature inside the aedeagus, but with a non-hooked protruding structure (unmodified in posterior 2/3, sclerotized in anterior 1/3) that reaches the tip of the temo (Fig. 6E1–2).

##### Distribution.

This small species occurs throughout the Ryukyu Islands (southern Kagoshima Prefecture and Okinawa Prefecture, Japan) as well as Taiwan.

##### Remarks.

This species appears to be closely allied with the similarly minute, long hind-legged Australian species *Acicnemis meriones* Pascoe, 1872, *A. arachnopus* Hubenthal, 1919, and the Sumatran species *A. ambigua* Hubenthal, 1919; however, phylogenetic analysis is required to confirm this. Preliminary observations suggest that some of the names in this group are synonyms.

#### ﻿ Acicnemis kiotoensis

Taxon classificationAnimaliaColeopteraCurculionidae

Nakane, 1963

CE8417E5-0CC2-5A60-A947-04F37FED721C

Figs 2C, 3B, 4A, 6F1–6, 2, 16

##### Type material examined

**(Japan: 73; Taiwan: 4; Total: 77). *Holotype*. Japan: Kyoto Prefecture**: • Kurama, 17.VI.1954, T. Nakane, bears red label reading “HOLOTYPE”, bears pink label reading “185-5”, Syst. Ent. Hokkaido Univ Japan SEHU 0000005125, HUM. ***Paratype*. Japan: Kagoshima Prefecture**: • Yakushima Island, Miyanoura, 28.IV.1954, Y. Kurosawa, bears red label reading “PARATYPE” (1, HUM).

**Figure 16.**
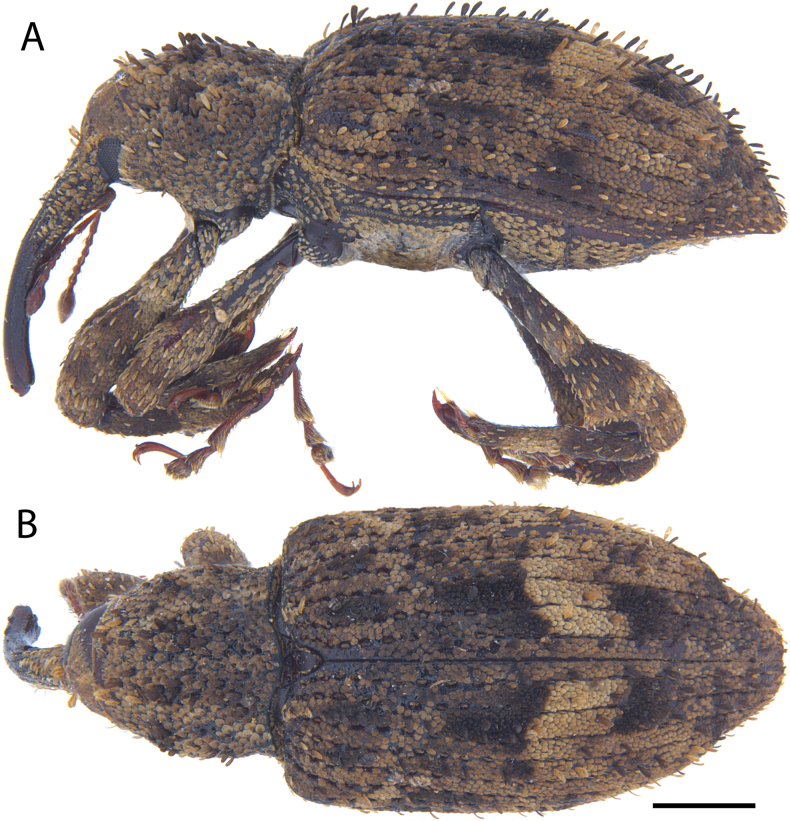
*Acicnemis kiotoensis* Nakane, 1963 (JHL_AREV_775). A. Lateral; B. Dorsal. Scale bar: 1 mm.

##### Non-type material.

**Japan: Kagoshima Prefecture**: • Kirishima, 18.IX.2007, J. Aoki (2, KUM), JHL_AREV_161, JHL_AREV_162; • Mt. Kirishima, 22.VII.1956, J. Nagao, (1, KUM), JHL_AREV_775; • Amami Oshima, Hatsuno, 25.V.1960, T. Shibata (1, KUM), JHL_AREV_177; • Chuorindo, Amami Oshima, 27.VI.1980, A. Izumi (1, LBM), LBM 5117; • Chuorindo, Amami Oshima, 30.VI.1980, Yoshikawa (1, LBM), LBM 5119; • Hatsuno, Amami Oshima, 1–8.VII.1973, M. Ito (1, LBM), LBM 5130; • Yuwan, Amami Oshima, 7.VIII.1964, M. Nagai (1, HUM), JHL_AREV_645; • Amami Oshima, 21.VII.1955, T. Shirozu (1, KUM), JHL_AREV_660; • Ishihara-rindo, Amami Oshima, 9–11.VI.1978, R. Noda (1, KUM), JHL_AREV_661; • Nonigawa, Amami Oshima, 17.VI.1963, J. Nagao (1, KUM), JHL_AREV_662; • Hatsuno, Amami Oshima, 27.V.1960, T. Shibata (1, ZMH), ZMH 837205; • Yudomari-rindo, Yakushima Island, 26.VII.1982, A. Seki (2, LBM), LBM 5120, 5121; • Miyanoura, Yakushima Island, 17.VII.1968, H. Nomura (1, LBM), LBM 5124; • Miyanoura, Yakushima Island, 21–22.VII.1974, K. Emoto (2, LBM), LBM 5126, 5127; • Miyanoura, Yakushima Island, 15–26.VII.1972, H. Huzita (2, LBM), LBM 5129, 5131; • Miyanoura, Yakushima Island, 26–29.VII.1974, T. Mikage (5, KUM), JHL_AREV_648 – JHL_AREV_650; • Miyanoura, Yakushima Island, 29.VIII.1952, Takeya & Hirashima (1, KUM), JHL_AREV_653; • Miyanoura, Yakushima Island, 18.VI.1975, Y. Kurosawa (1, NMNS), JHL_AREV_679; • Okawa-rindo, Yakushima Island, 1–2.VIII.1974, T. Mikage (2, KUM), JHL_AREV_646, JHL_AREV_647; • Okawa, Yakushima Island, 22.VII.1975, H. Akiyama (1 dissected male, NMNS), JHL_AREV_157; • Shiratani, Yakushima Island, 26–30.VII.1979, M. Miura (8, HUM), JHL_AREV_634 – JHL_AREV_641; • Shiratani, Yakushima Island, 24–25.VII.1974, T. Mikage (5, KUM), JHL_AREV_657 – JHL_AREV_659; • Kosugidani, Yakushima Island, 19.VII.1974, T. Mikage (1, KUM), JHL_AREV_656; • Kosugidani, Yakushima Island, 23.VII.1950, T. Shirozu (1, KUM), JHL_AREV_655; • Kosugidani, Yakushima Island, 9.–10.VII.1952, Y. Kurosawa (4, NMNS), NSMT-I-C 10267–10269, NSMT-I-C 10301; • Hanayama Natural Forest, Yakushima Island, 21.VIII.1983, Sk. Yamane (3, HUM), JHL_AREV_642 – JHL_AREV_644; • Mt. Hoyoshi, Minamiosumi-cho, 24–25.III.2018, R. Ito (1, KUM), JHL_AREV_840; • Mt. Yuwan, Amami-Oshima, 30.VII.1963, L. Gressitt (1 dissected male, KUM), JHL_SYN_152; **Kyoto Prefecture**: • Mt. Kurama, 30.V.1982, K. Masaki (1, LBM), LBM 5118; • same locality, 8.X.1978, K. Masaki (1, LBM), LBM 5122; **Miyazaki Prefecture**: • Miyakonojo-shi, Natsu-cho, Miike, 22.VI.2019, R. Ito (1, KUM), JHL_AREV_175; • Mt. Aoidake, 7.VIII.1974, K. Morimoto (1 dissected male, KUM), JHL_DNA_147; **Nagasaki Prefecture**: • Mt. Taterasan, Izuhara, Tsushima Island, 24–27.VII.1985, A. Saito (15, CMIC), CBM – ZI 68476–68490; • Mt. Tatera, Tsushima Island, 18–20.V.1961, H. Kamiya (2, KUM), JHL_AREV_651, JHL_AREV_652; **Nara Prefecture**: • Kasugayama, 25.VII.1981, K. Masaki (1, LBM), LBM 5116; • Kasugayama, 10.V.1964, K. Kinugasa (1, LBM), LBM 5133; • Kasugayama, 20.V.1951, K. Sawada (1, KUM), JHL_AREV_654; • Mt. Kasuga, 9.V.1952, K. Sawada (1 dissected male, KUM), JHL_SYN_153; **Okinawa Prefecture**: • Okinawa Island, Yonahadake, 8–29.VI.1977, H. Irie (2, KUM), JHL_AREV_172, JHL_AREV_173; • Iriomote Island, Shirahama, 23–24.VI.1970, H. Makihara (2, KUM), JHL_AREV_158, JHL_AREV_159; • same locality, 31.VIII–5.IX.1969, H. Makihara (6, KUM), JHL_AREV_163 – JHL_AREV_168; • same locality, 26.VII.1963, Y. Miyatake (1, KUM), JHL_AREV_169; • Okinawa Island, Yona, 9–13.VIII.1969, H. Makihara (1, KUM), JHL_AREV_176; • Iriomote Island, Nakara River, 25–28.VI.1970, H. Makihara (2, KUM), JHL_AREV_170, JHL_AREV_171; • Iriomote Island, Taketomi-cho, Mt. Tedo, 2.X.2017, K. Narita (1, KUM), JHL_AREV_174; • Iriomote Island, 28.VI.1978, M. Kinjo (1, RUMC), JHL_AREV_524; • Iriomote Island, Funaura, 8.X.1977, M. Arasaki (1, RUMC), JHL_AREV_525. **Taiwan** • Mt. Hsin Kao, 1.VII.1961, S. Ueno, JHL_AREV_160 (1, KUM); • Meifeng, 15.V.1983, H. Townes (1, CMNC), JHL_AREV_667; • Anmashan, Taichung Hsien, 11–15.V.1992, A. Smetana (2, CMNC), JHL_AREV_707, JHL_AREV_708.

##### Diagnosis.

Body length 4.5–7.0 mm. Covered in pale and dark brown scales, with two obliquely shaped pale brownish, black-bordered spots across middle of elytra. Second funicular antennomere longer than funicular antennomeres 3 + 4 (Fig. 2C) (compare with *A. koguma*, which has the second funicular antennomere shorter than funicular antennomeres 3 + 4). Pronotum lacking impunctate longitudinal midline (present in *A. koguma*). Scutellum diagnostic: large, shiny, and lacking scales (Fig. 3B). Odd and 2^nd^ elytral intervals with erect scales (scales beginning at middle of elytra on 2^nd^ interval; from elytral base on odd intervals). Sclerolepidia indistinct, forming low, rounded, non-protruding tubercles (Fig. 4A). Third tarsomeres emarginate (not truncate as in *A. koguma*). Pedon with lateral edges approx. parallel in dorsal view but converging evenly into a wide, blunt point. Internal sac not visible inside the aedeagus, but with a non-hooked protruding structure (unmodified in posterior 1/2, with roughened in anterior 1/2) that is clearly longer than the pedon (Fig. 6F1–2).

##### Distribution.

This distinctive species occurs in Japan (mainland, throughout the Ryukyu Islands) and Taiwan.

#### ﻿ Acicnemis koguma

Taxon classificationAnimaliaColeopteraCurculionidae

Lewis & Kojima sp. nov.

6FA0DC04-D964-5807-AD2E-8B6E193B52DE

https://zoobank.org/78DAC1B8-51AE-4ABC-9E44-27A21760C117

Figs 2D, 3C, 17

##### Type material examined

(Japan: 3). ***Holotype*. Japan: Wakayama Prefecture**: • Mt. Otoh, 20.VII.1980, I. Matoba, bears a red holotype label reading “HOLOTYPE / *Acicnemis koguma* / Lewis & Kojima, 2025”, JHL_DNA_143, female, deposited in KUM. ***Paratypes*. Japan** • **Tokyo Metropolis**: Mt. Takao, 12.VIII.1969, H. Fujita, LBM accession # 5128 (1, LBM), JHL_SYN_141; • Mt. Takao, 3–4.VII.1965, K. Sakai (1, CMIC), CBM-ZI 68474.

**Figure 17.**
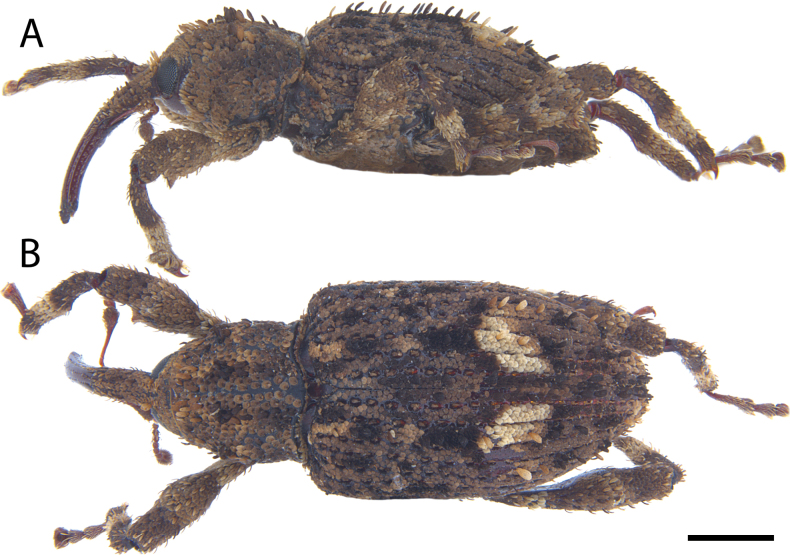
*Acicnemis koguma* Lewis & Kojima, sp. nov. (JHL_SYN_141). A. Lateral; B. Dorsal. Scale bar: 1 mm.

##### Diagnosis.

Body length 4.5–7.0 mm. Covered in pale and dark brown scales, with two obliquely shaped pale brownish, black-bordered spots across middle of elytra. Second funicular antennomere shorter than funicular antennomeres 3 + 4 (Fig. 2D) (longer in *A. kiotoensis*). Pronotum with impunctate longitudinal midline (absent in *A. kiotoensis*). Scutellum large (somewhat narrower than *A. kiotoensis*) and bare dorsally but bordered laterally by pale yellow scales (Fig. 3C). Only odd elytral intervals with erect scales (some erect scales on 2^nd^ interval in apical fifth of elytra) (*A. kiotoensis* with erect scales on the 2^nd^ elytral interval). Sclerolepidia indistinct, forming low, rounded, non-protruding tubercles. Third tarsomeres truncate (emarginate in *A. kiotoensis*).

##### Distribution.

This rare species is only known from two localities in mainland Japan, Mt. Takao (Tokyo Metropolis) and Mt. Otoh (Wakayama Prefecture).

##### Etymology.

The specific name *koguma* means *little bear* in Japanese, and is a reference to the apparent mountain-loving preference and small, brown nature of this rarely observed weevil species. We also suggest the Japanese common name コグマカレキゾウムシ [Koguma-kareki-zoumushi], which in English means “Little-bear-dead-tree-weevil”.

##### Remarks.

This cryptic species was clearly overlooked due to its remarkable similarity with *A. kiotoensis* in overall color and appearance, but differs consistently in the five characters listed in the diagnosis. The male remains unknown.

#### ﻿ Acicnemis laeta

Taxon classificationAnimaliaColeopteraCurculionidae

Hubenthal, 1919

BBBD59AA-596D-5FC7-A6EC-A6D76421B4F8

Figs 1, 2B, 6G1–6, 2, 18

##### Type material examined

**(Taiwan: 71). *Lectotype* (designated here). Taiwan** • Fuhosho, “IX.07–09”, Sauter, SDEI, SDEI Muncheberg Col 20204, bears the original red label reading “Syntypus” as well as a new, red lectotype label reading “LECTOTYPE / *Acicnemis laeta* / Hubenthal, 1919 / Det. Lewis & Kojima, 2025”. ***Paralectotypes*. Taiwan** • Fuhosho, “IX.07–09”, Sauter (22, SDEI), SDEI Muncheberg Col 18617–18623 & 20199–20203 & 20205–20214, all bear a red label reading “Syntypus” and new, yellow paralectotype labels; • Hoozan, “08–10”, Sauter (18, SDEI), SDEI Muncheberg Col 18624–18632 & 20215–20223, all bear a red label reading “Syntypus” and new, yellow paralectotype labels; • Kosempo, Sauter (2, SDEI), SDEI Muncheberg Col 18633–18634, bear a red label reading “Syntypus” and new, yellow paralectotype labels.

**Figure 18.**
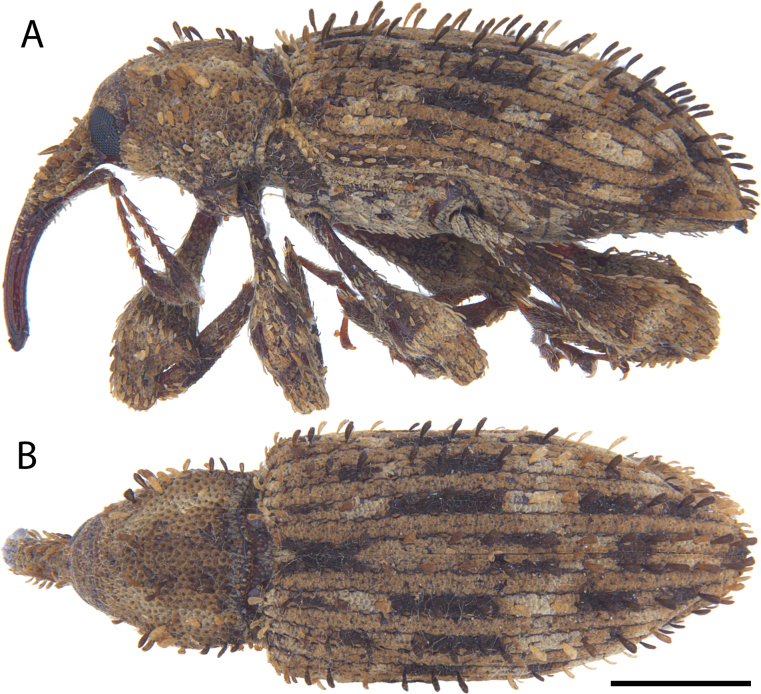
*Acicnemis laeta* Hubenthal, 1919 (JHL_AREV_773). A. Lateral; B. Dorsal. Scale bar: 1 mm.

##### Non-type material.

**Taiwan** • Lingui, 18.VI.1979, S. Nakamura, W. Chen (1, KUM), JHL_AREV_526; • Pi Shan Spa, Tai Tung Hsein, 2.VI.1987, K. Baba (2, KUM), JHL_AREV_527, JHL_AREV_528; • Guandaoxi, I.VI.1973, K. Kojima (2, KUM), JHL_AREV_529, JHL_AREV_530; • Jihyueh-tan, Nantou Hsein, 12.V.1977, H. Fujita (1, KUM), JHL_AREV_531; • Kenting Park, Pingtung Hsein, 11.V.1970, M. Takagi (1, KUM), JHL_AREV_532; • Nanshanchi, Nantou Hsein, 7.V.1977, T. Niisato (1, KUM), JHL_AREV_533; • Liu Kui, 28.III.–10.IV.1986, K. Baba (2, KUM), JHL_AREV_534, JHL_AREV_535; • Kenting, Pingtung Hsein, 2.IV.1965, S. Ueno (2, KUM), JHL_AREV_536, JHL_AREV_537; • Nanshanchi, 30.IV.1965, T. Nakane, T. Shirozu (2, KUM), JHL_AREV_538, JHL_AREV_539; • Nanshanchi, Nantou Hsein, 12.VII.1966 (1, KUM), JHL_AREV_540; • Shyk Shan, near Liu Kui, 25.IV.1986, K. Baba (1 dissected male, KUM), JHL_AREV_541; • Nanshanchi, 3.V.1979, K. Emoto (1, KUM), JHL_AREV_664; • Lienhoachi, 12.V.1975, S. Imasaka (1, TUA), JHL_AREV_665; • “community park”, 28.X.1976 (1, TUA), JHL_AREV_666; • Nantou, 24.V.1983, K. Ra, (1, HUM), JHL_AREV_773; • Wushe, 29.IV.–3.V.1983, H. Townes (3, CMNC), JHL_AREV_669 – JHL_AREV_671; • Liukei, Kaohshiiung, 29.IV.–8.V.1982, H. Takizawa (3, HUM), JHL_AREV_675 – JHL_AREV_677; • near Liukuei, Kaohsiung Hsien, 5–9.IV.1995, H. Kojima (1, TUA), JHL_AREV_690; • “Formosa”, T. Kano (1, NMNS), JHL_AREV_680; • Kao; • Nanshan Riv. Trail, Ren’ai Township, Nantou County, 18–19.X.2017, K. Narita (2, KUM), JHL_DNA_124 (GenBank accession number: PV255630), JHL_AREV_125 (GenBank accession number: PV255631).

##### Diagnosis.

Body length 4.1–5.8 mm. Covered in pale brown scales, with alternating white and black scaled bands on odd elytral intervals. Scutellum covered in yellow to brown scales. Only odd elytral intervals with erect scales. Sclerolepidia distinct and protruding. Posterior edge of prosternum with prominent projections contiguous with the fore-coxae (Fig. 2B). Third tarsomeres distinctly emarginate. Pedon with lateral edges approx. parallel in dorsal view, but converging evenly into a moderately acute point in apical 1/2. Internal sac protruding and with two bead-shaped sclerotized structures in basal 1/2, and a vase-shaped structure in apical 1/2 with roughened musculature (Fig. 6G1–2).

##### Distribution.

We only encountered this species from Taiwan. The records from Japan and the Kuril Islands (Russia / Japan) noted in Alonso-Zarazaga et al. (2023) are almost certainly erroneous and this species may have been confused with *A. azumai*. This species may plausibly occur in mainland Asia (China) (listed as occurring there by Alonso-Zarazaga et al. (2023)), but we have yet to examine any specimens of *A. laeta* from this region.

##### Remarks.

This species is superficially quite similar to *A. azumai*, but differs in pronotal, prosternal, and genital morphology.

#### ﻿ Acicnemis luteomaculata

Taxon classificationAnimaliaColeopteraCurculionidae

Morimoto & Miyakawa, 1995

DDDC7371-7A1C-5A5B-942A-07135233633F

Figs 1, 2E, 6H1–6, 2, 19

##### Type material examined

**(Japan: 45). *Holotype*. Japan: Fukuoka Prefecture**: • Hiko-san, 13–14.VI.1957, K. Morimoto, bears red label reading “(HOLOTYPE) Acicnemis luteomaculata Morimoto et. Miyakawa, 1994”, ELKU, ELKU 2961. ***Paratypes*. Japan: Ehime Prefecture**: • Hojo-shi, Mt. Takanawa, 18.V.2003, T. Kurihara (1, KUM), JHL_AREV_420; **Fukuoka Prefecture**: • Hiko-san, 8.VI.–13.VII.1957, K. Morimoto (11, KUM), JHL_AREV_181 – JHL_AREV_190, JHL_ACITAI_090 (dissected male); • same locality, 4.VI.1958, K. Morimoto (2, KUM), JHL_AREV_192, JHL_AREV_193; • same locality, 10.VII.1938, Hori, Kawahara & Yasumatsu (1, KUM), JHL_AREV_194; • Mt. Wakasugi, 18.XII.1955, K. Oshima (1, KUM), JHL_AREV_120; • Wakasugi, 28.VII.1940, T. Shirozu (2, KUM), JHL_AREV_405, JHL_AREV_406; **Fukushima Prefecture**: • Mt. Shizukura-yama, Mishima Town, 37.3756°N, 139.6266°E, 11–20.VII.2018, K. Narita (1, KUM), JHL_AREV_412; **Kochi Prefecture**: • Mt. Tebako, 7–10.VIII.1957, K. Morimoto (2, KUM), JHL_AREV_196, JHL_AREV_197; **Nara Prefecture**: • Mt. Odaigahara, 2.VII.1978, H. Hiramatsu (1, KUM), JHL_AREV_198; • Kasugayama, 25.VII.1959, T. Hozumi (1, KUM), JHL_AREV_121; • Kasuga, 5–31.V.1959, K. Ueda (3, KUM), JHL_AREV_401 – JHL_AREV_403; • Kasuga, 30.IV.1961, K. Ueda (1, KUM), JHL_AREV_404; **Okayama Prefecture**: • Takahashi City, Mt. Gagyuzan, 19–20.V.1975, H. Irie (3, KUM), JHL_AREV_178 – JHL_AREV_180; **Wakayama Prefecture**: • Mt. Gomadan, 28.VII.1979, I. Matoba (1, KUM), JHL_AREV_191.

**Figure 19.**
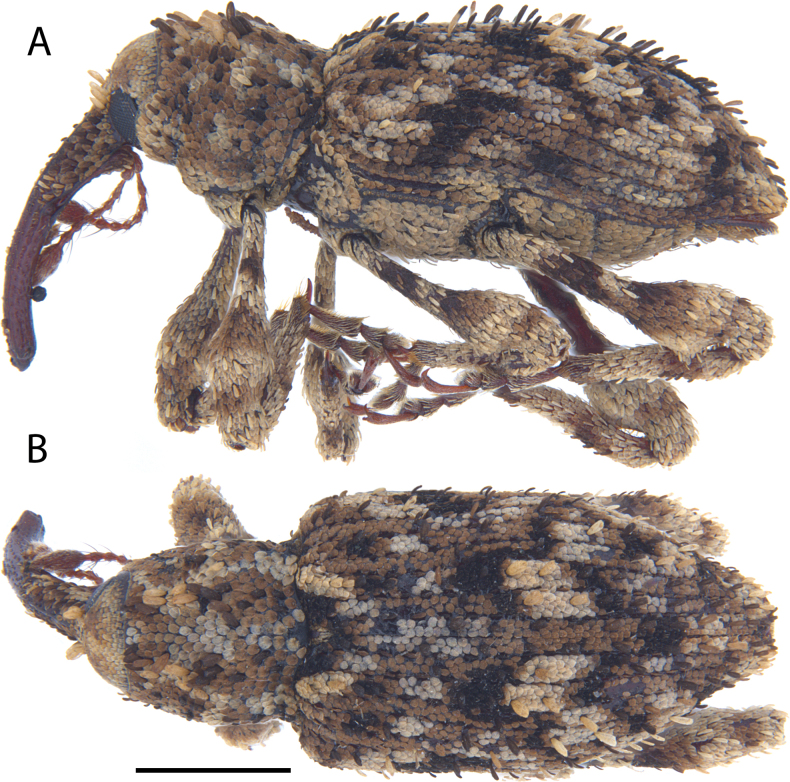
*Acicnemis luteomaculata* Morimoto & Miyakawa, 1995 (JHL_AREV_409). A. Lateral; B. Dorsal. Scale bar: 1 mm.

##### Non-type material.

**Japan: Ehime Prefecture**: • Hojo-shi, Mt. Takanawa, 18.V.2003, T. Kurihara (1, KUM), JHL_AREV_420; • Ishizuchi Mt., Omogo Valley, 18–25.VIII.1980, S. & J. Peck (1, CMNC), JHL_AREV_668; **Fukuoka Prefecture**: • Hiko-san, 23–26.VII.1968, M. Chujo (1, KUM), JHL_AREV_195; **Hiroshima Prefecture**: • Sandankyo, 12–13.V.1969, M. Chujo (1, KUM), JHL_AREV_418; **Miyazaki Prefecture**: • Miyakonojo-shi, Natsuo-cho, Miike, 22.VI.2019, R. Ito (6, KUM), JHL_AREV_407 – JHL_AREV_411, JHL_DNA_117 (GenBank accession number: PV255632); **Nara Prefecture**: • Kasuga, 10.V.1959, T. Shibata (1, KUM), JHL_ACITAI_091; **Oita Prefecture**: • Mt. Kuro-dake, 28.V.1990, S. Ogata (4, KUM), JHL_AREV_413 – JHL_AREV_416; • Tsukahara, Yufuin-cho, Yufu-shi, 21.XI.2016, R. Ito (1, KUM), JHL_AREV_721; • Tsukahara, Yufuin-cho, Yufu-shi, 27.V.2018, R. Ito (1, KUM), JHL_DNA_123 (GenBank accession number: PV255633); **Wakayama Prefecture**: • Komoridani, 21.VIII.1941, K. Sakaguchi (1, HUM), JHL_AREV_678; **Yamagata Prefecture**: • Tozawa-mura, Mt. Haguro, 23.VI.2003, J. Aoki (1, KUM), JHL_AREV_417.

##### Diagnosis.

Body length 4.0–5.4 mm. Covered in pale brown scales, with two obliquely shaped tan-colored, black-bordered spots across middle of elytra. Pronotum lacking impunctate longitudinal midline (present in *A. koguma*). Scutellum sparsely covered in pale yellow scales. All elytral intervals with erect scales. Sclerolepidia indistinct, forming low, rounded, non-protruding tubercles. Third tarsomeres distinctly truncate (Fig. 2E) (compare with the superficially similar *A. kiotoensis*, *A. maculaalba*, and *A. sauteri*, which all have emarginate third tarsomeres). Pedon with lateral edges approx. parallel in dorsal view, but converging evenly into a blunt, rounded point. Internal sac not visible inside the aedeagus, but with a basal projection that bears a distinctive rectangular sclerite anteriorly at its apex (Fig. 6H1–2).

##### Distribution.

This species is found in mainland Japan (except Hokkaido) and has also been reported from South Korea (Park et al. 2009).

#### ﻿ Acicnemis maculaalba

Taxon classificationAnimaliaColeopteraCurculionidae

Roelofs, 1875

87491871-1E50-5A9B-AD08-F51AD234E18C

Figs 1, 2G, 3D, 6I1–6, 2, 20

##### Type material examined

**(Japan: 50). *Holotype*. Japan** • unknown locality, “1910–320”, G. Lewis, bears a circular white, red-bordered label reading “Type H.T.”, NHMUK015024192.

**Figure 20.**
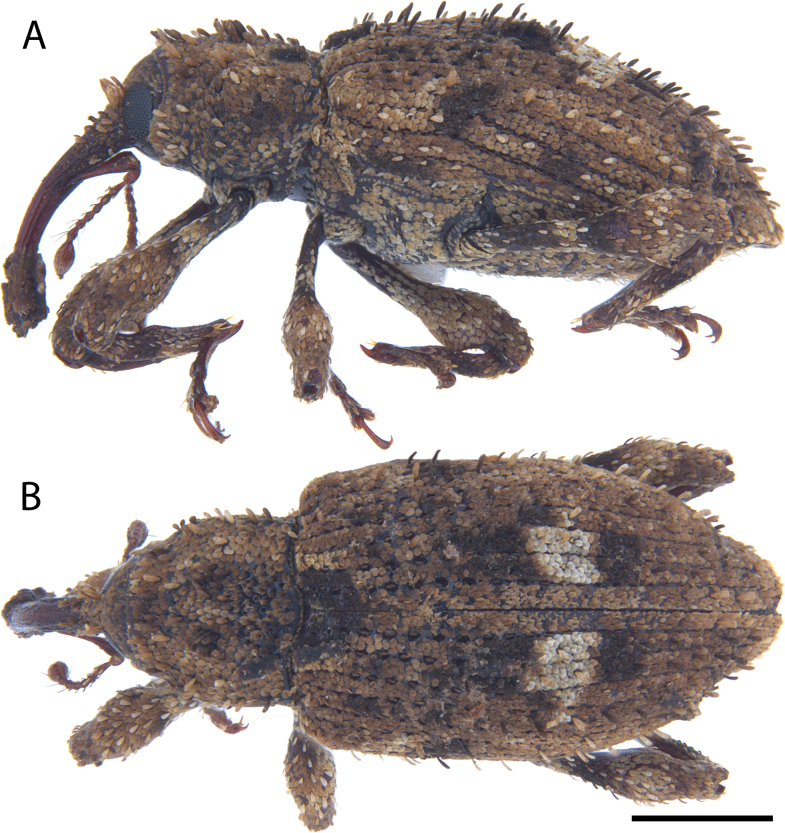
*Acicnemis maculaalba* Roelofs, 1875 (JHL_AREV_777). A. Lateral; B. Dorsal. Scale bar: 1 mm.

##### Non-type material.

**Japan: Fukuoka Prefecture**: • Mt. Homan, 15.V.1975, H. Irie (1, KUM), JHL_AREV_583, misidentified *A. luteomaculata* paratype; **Kagoshima Prefecture**: • Sata (Osumi), Magome-Odomari, 28.V.1952, Esaki & Hirashima (1, KUM), JHL_AREV_556; • Kuchinoshima, 26.VI.–3.VII.1969, H. Makihara (3, KUM), JHL_AREV_557 – JHL_AREV_559; • Akuseki Island, 30.VII.1969, M. Sakai (4, KUM), JHL_AREV_560 – JHL_AREV_563; • Nakanoshima, 25.IV.–2.V.1975, H. Irie (4, KUM), JHL_AREV_564 – JHL_AREV_567; • Amami-Oshima, Hatsuno, 12.VI.1963, J. Nagao (1, KUM), JHL_AREV_570; • Yakushima Island, Miyanoura, 26.VII.1974, T. Mikage (1, KUM), JHL_AREV_571; • Funayuki, Yakushima Island, 26.VII.1989, M. Satoshi (1, CMIC), CBM ZI 159703; **Kochi Prefecture**: • Okinoshima (Tosa Province), 28.VI.–2.VIII.1953, K. Morimoto & K. Sugimoto (5, KUM), JHL_AREV_551 – JHL_AREV_555; **Mie Prefecture**: • Mie University Forest, Ichishi-gun, 11.VI.1956, M. Ichihashi (2, KUM), JHL_AREV_546, JHL_AREV_547; **Miyazaki Prefecture**: • Mt. Aoidake, 6.V.1976, K. Morimoto, Z. Kuranaga, A. Iwasaki, N. Yoshida (1, KUM), JHL_AREV_590; **Okinawa Prefecture**: • Komi, Iriomote Island, 14.III.1997, M. Chujo (1, KUM), JHL_SYN_143; • Iriomote Island, 28.V.1962, K. Kojima (1, KUM), JHL_AREV_545; • Yonahadake, 8–29.VI.1977, H. Irie (2, KUM), JHL_AREV_568, JHL_AREV_569; • Hiji, Okinawa Island, 3.VII.1974, T. Mikage (2, KUM), JHL_AREV_572, JHL_AREV_573; • Yona, Okinawa Island, 15.VI.1970, H. Makihara (1, KUM), JHL_SYN_142; • Yona, Okinawa Island, 29.VI.1976, H. Makihara (1, KUM), JHL_AREV_574; • Mt. ufudaki, Kohama Is., 29.V.2022, S. Imada, (1, KUM), JHL_AREV_777; • Mantabaru, Yonaguni Island, 25–27.V.2022, S. Imada (3, KUM), JHL_AREV_575 – JHL_AREV_577; • Omoto-dake, Ishigaki Island, 30.VI.1974, T. Mikage (1, KUM), JHL_AREV_578; • Mt. Banna, Ishigaki Island, 23–26.V.1990, K. Morimoto (1, KUM), JHL_AREV_579; • Shirahama, Iriomote Island, 31.VIII.–5.IX.1969, H. Makihara (3, KUM), JHL_AREV_580 – JHL_AREV_582; • Funaura, Iriomote Island, 1.X.1978, S. Azuma (1, RUMC), JHL_AREV_713; • Nakagusuku Park, Okinawa Island, 26.28557°N, 127.79491°E, 3–17.II.2016, OKENT0068361 (GenBank accession number: PV255635) (1, OIST); • Yona Field, Okinawa Island, 26.73974°N, 128.23598°E, 8–22.VII.2016, OKENT0050489 (GenBank accession number: PV255634) (1, OIST); • **Toyko District**: Oji-ike, Miyake Island, 5–7.IV.2010, J. Aoki (6, KUM), JHL_AREV_584 – JHL_AREV_589; • Sato, Mt. Kurosa – Kitakao-san, Mikura-jima Island, 2.VI.1979, J. Okuma (1 dissected male, KUM), JHL_AREV_426; **Wakayama Prefecture**: • near Mt. Ohtoh, 13.VI.–7.VIII.1980, I. Matoba (2, KUM), JHL_AREV_548, JHL_AREV_549; • Mt. Ohtoh, 16–19.VII.1982, H. Makihara (1, KUM), JHL_AREV_500.

##### Diagnosis.

Body length 2.6–5.0 mm. Covered in pale and dark brown scales, with two obliquely shaped pale brownish, black-bordered spots across middle of elytra. Pronotum lacking impunctate longitudinal midline (present in *A. koguma*). Scutellum small, bordered by pale, yellow scales, and weakly bare dorsally or covered in scales (Fig. 3D) (compare with *A. kiotoensis* which possesses a large, broad, bare scutellum). Odd, 2^nd^, and 4^th^ elytral intervals with erect scales. Sclerolepidia indistinct, forming low, rounded, non-protruding tubercles. Third tarsomeres emarginate (Fig. 2G) (as opposed to truncate, as in *A. koguma*). Pedon with lateral edges approx. parallel in dorsal view but converging in distal 1/2 evenly into a blunt point. Pedon stout (pedon length / total aedeagus length including temo = 0.2). Internal sac protruding and with roughened musculature across length; with two bent, tube-like sclerotized structures anteriorly at its apex (Fig. 6I1–2).

##### Distribution.

This species occurs broadly throughout Japan (mainland Japan and Ryukyu Islands) and Taiwan and is also recorded from China by Alonso-Zarazaga et al. (2023).

##### Remarks.

*Acicnemis maculaalba* forms a species group with *A. sauteri*. The association of *A. maculaalba* and *A. sauteri* is supported by morphology and weakly (BS: 38) by our CO1 gene tree. See also *A. cordata* Remarks section.

#### ﻿ Acicnemis nobilis

Taxon classificationAnimaliaColeopteraCurculionidae

Hubenthal, 1919

3A98A048-6003-545B-A301-8AA6A9CF6822

Figs 6J1–6, 2, 21

##### Type material examined

**(Taiwan: 10). *Holotype*. Taiwan** • Hoozan, “H. Sauter 1910”, bears small yellow label reading “1911 14”, bears red label reading “Typus”, SNSD.

**Figure 21.**
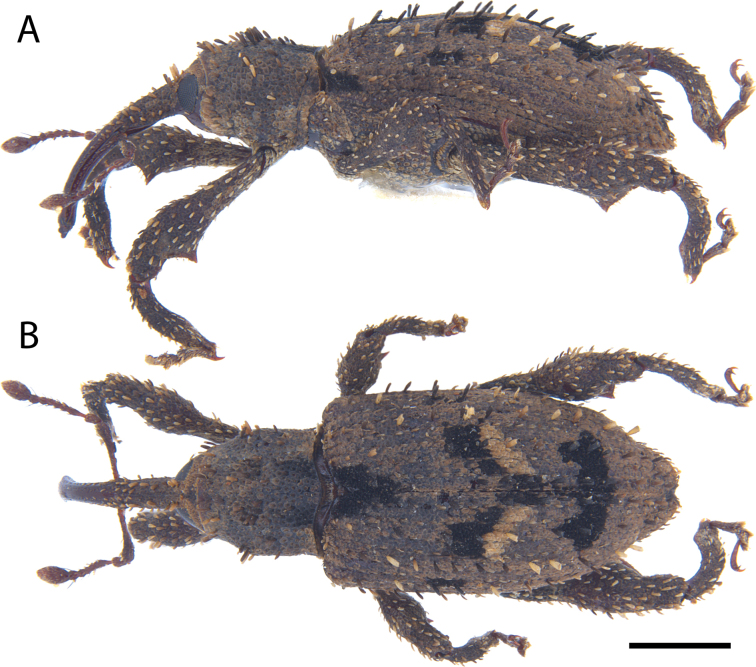
*Acicnemis nobilis* Hubenthal, 1919 (JHL_AREV_496). A. Lateral; B. Dorsal. Scale bar: 1 mm.

##### Non-type material.

**Taiwan** • Penpuchi, Nantou Hs., 13.VII.1966, H. Sasaji (2, KUM), JHL_SYN_151 (dissected male), JHL_AREV_492; • Sangkang, Nantou Hs., 14.VII.1966, H. Sasaji (3, KUM), JHL_SYN_150 (dissected male), JHL_AREV_491, JHL_AREV_493; • Lushan, Nantou Hsein, 8.VI.1976, H. Makihara (1, KUM), JHL_AREV_494; • Nanshanchi, Wenchuan Hs., 27.IV.–4.V.1977, T. Mikage (1, KUM), JHL_AREV_495; • Hsi-Leng, 3.V.1981, S. Tsuyuki (1, TUA), JHL_AREV_496; • Kaohsiung, 5.VIII.1978, T. Niisato (1, TUA), JHL_AREV_497.

##### Diagnosis.

Body length 4.1–5.2 mm. Covered in dark gray and black scales, with a pale-yellow scale, V-shaped, transverse band across middle of elytra. Basal patch of black scales surrounding scutellum approx. V-shaped and extending prominently onto the second elytral intervals (as opposed to the similar *A. nohirai* which has an elongate I-shaped patch of black scales on the first elytral interval that barely extends on the second elytral interval basally). Pronotum lacking impunctate longitudinal midline (present in *A. koguma*). Scutellum covered in pale scales. Elytra with erect scales only on odd intervals. Sclerolepidia distinct and protruding. Third tarsomeres distinctly emarginate. Pedon with lateral edges approx. parallel in dorsal view, but sinuating into a blunt, rounded point. Internal sac protruding from aedeagus, with roughened musculature posteriorly and a club-shaped sclerotized structure anteriorly at its apex (Fig. 6J1–2).

##### Distribution.

This species is currently only known from Taiwan.

#### ﻿ Acicnemis nohirai

Taxon classificationAnimaliaColeopteraCurculionidae

Morimoto & Miyakawa, 1995

99B40C3B-45FA-56D2-8318-1925B0964925

Figs 1, 6K1–6, 2, 22

##### Type material examined

**(Japan: 23). *Holotype*. Japan: Gifu Prefecture**: • Hirayu, 19.VI.1963, T. Nohirai, bears a red label reading “(Holotype) Acicnemis nohirai Morimoto et Miyakawa, 1994”, ELKU, ELKU 2956. ***Paratypes*. Japan: Aomori Prefecture**: • Kawauchi, Shimokita-gun, 25.VII.1984, S. Yamauchi (1, KUM), JHL_AREV_435; **Ehime Prefecture**: • Omogokei, 15.VI.1981, S. Naomi (1, KUM), JHL_AREV_437; **Gifu Prefecture**: • Hirayu, 19.VI.1963, T. Nohirai (5, KUM), JHL_AREV_429 – JHL_AREV_433; • **Hokkaido**: Horoka, Kamishihoro-cho, 17.VII.1976, H. Irie (1, KUM), JHL_AREV_434; • Uchida, 18.V.1930 (1, HUM), JHL_AREV_454; • Sapporo, “3.VIII.80” (1, KUM), JHL_AREV_460; **Nagano Prefecture**: • Tobira Spa, Matsumoto, 7.VIII.1973, T. Mikage (1 dissected male, KUM), JHL_SYN_147; **Niigata Prefecture**: • Kurokawa, 30.VII.1962, K. Baba (1 dissected male, KUM), JHL_SYN_146; **Oita Prefecture**: • Kurasame, Kuju, 15.VI.1975, H. Irie (2, KUM), JHL_AREV_438, JHL_AREV_439; **Tochigi Prefecture**: • Chuzenji-Yumoto, Shimotsuke, 22.VII.1923, T. Esaki (1, KUM), JHL_AREV_436; **Yamanashi Prefecture**: • Fuji-rindo, Mt. Fuji, 10–12.VIII.1976, H. Irie (1, KUM), JHL_AREV_440; • Mt. Kaikoma, 13.VIII.1962, K. Oshima (1, KUM), JHL_AREV_441; • Masutomi, Nirasaki, 16.VII.2006, H. Takizawa (1, HUM), JHL_AREV_461.

**Figure 22.**
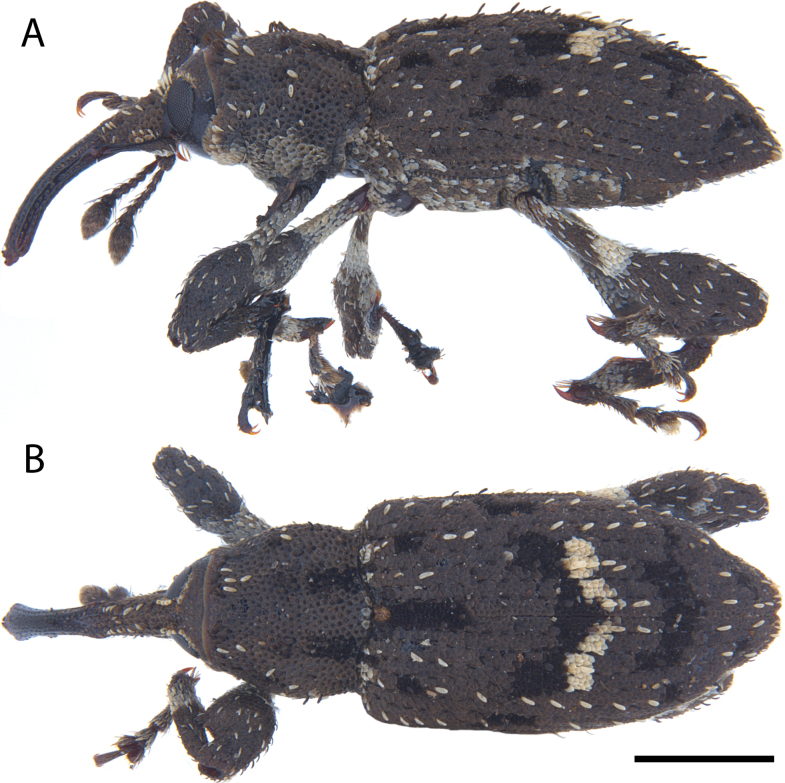
*Acicnemis nohirai* Morimoto & Miyakawa, 1995 (JHL_AREV_774). A. Lateral; B. Dorsal. Scale bar: 1 mm.

##### Non-type material.

**Japan: Gifu Prefecture**: • Hirayu, 19.VI.1963, T. Nohirai (1, KUM), JHL_AREV_442; **Nagano Prefecture**: • Otaki-mura, 31.V.2010, K. Takahashi (1, KUM), JHL_DNA_119 (PV255636); **Oita Prefecture**: • Yuhu-shi, Yuhuin-chou, Tsukahara, 18.IX.2016, R. Ito (1, KUM), JHL_DNA_122 (PV255637); **Yamanashi Prefecture**: • Fuji-rindo, Mt. Fuji, 3.X.1992, T. Mikage (1, KUM), JHL_AREV_443; • Mt. Daibosatsu, Hagiwara, 12.VIII.2005, T. Kurihara (1, KUM), JHL_AREV_720; • Masutomi, 24–25.V.1968, H. Takizawa, (1, HUM), JHL_AREV_774.

##### Diagnosis.

Body length 4.9–6.0 mm. Covered in dark gray and black scales, with a white-scaled, V-shaped, transverse band across middle of elytra. Elytra with elongate I-shaped patch of black scales on the first elytral interval (surrounding the scutellum) that barely extends onto the second elytral interval. Pronotum lacking impunctate longitudinal midline (present in *A. koguma*). Scutellum covered in dark brown scales. Elytra with erect scales only on odd intervals. Sclerolepidia distinct and protruding. Third tarsomeres distinctly emarginate. Pedon with lateral edges approx. parallel in dorsal view, but converging evenly into a blunt, rounded point. Internal sac not visible inside aedeagus, but with protruding basal structure that bears a prong-shaped sclerite anteriorly at its apex (Fig. 6K1–2).

##### Distribution.

This species is apparently endemic to mainland Japan and occurs from Hokkaido south to Kyushu (Oita Prefecture).

#### ﻿ Acicnemis palliata

Taxon classificationAnimaliaColeopteraCurculionidae

Pascoe, 1872

2D859A73-57DE-559B-94B2-4FCD616645CF

Figs 3E, 4B, 6L1–6, 2, 23

Acicnemis dorsonigrita Voss, 1941, syn. nov.

##### Type material examined

**(China: 2; Japan: 20: Total: 22). *Holotype*. Japan: Nagasaki Prefecture**: • Nagasaki, Pascoe Coll. 93–60, bears a circular white, red-bordered label reading “Type H.T.”, NHMUK015026632. ***Paratypes*. China** • Tienmuschan, bears a red label reading “Type” (1 dissected male, NHMB), *Acicnemis dorsonigrita* type specimen, JHL_ACITAI_010; • Tienmuschan, bears a red label reading “Cotypus *Acicnemis dorsonigrita*” (1, ZMH), ZMH 841827.

**Figure 23.**
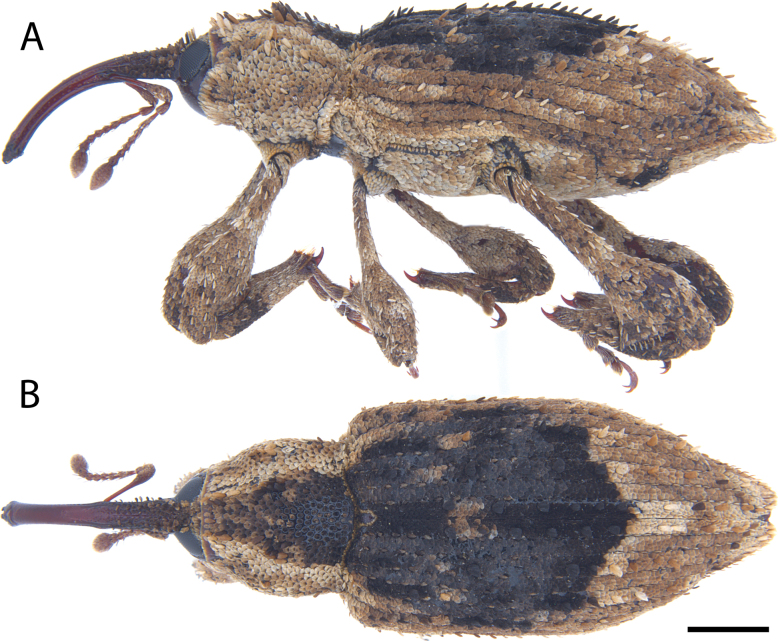
*Acicnemis palliata* Pascoe, 1872 (JHL_AREV_779). A. Lateral; B. Dorsal. Scale bar: 1 mm.

##### Non-type material.

**Japan: Chiba Prefecture**: • Ichihara-shi, Kongouuchi, 28.V.1989, M. Satoshi (2, CMIC), CBM-ZI 159709, CBM-ZI 159710; **Ehime Prefecture**: • Higashino, Matsuyama-shi, 1–22.VII.2016, Y. Hisasue (2, KUM), JHL_AREV_484, JHL_AREV_485; **Fukuoka Prefecture**: • Chikuzen, Kashii, Kusaba Kosen, 5.VI.1935, Esaki, Hori, Ho, Fujino, Yasumatsu, Nomura & Kawahara (1, KUM), JHL_ACITAI_046; • Mt. Homan, 2.VII.1978, R. Noda (1 dissected male, KUM), JHL_ACITAI_007; • Nokonosima, 8–29.V.2005, H. Hirano (2, KUM), JHL_AREV_486, JHL_AREV_487; **Fukushima Prefecture**: • 1.VII.2000, K. Takahashi (1, KUM), JHL_AREV_483; • Kaneyama-machi, Okuriyama, 2.VI.2012, J. Aoki (3, KUM), JHL_AREV_488; • Aizu, Kaneyama, 20–21.VII.2012, J. Aoki (4, KUM), JHL_AREV_489; **Kagawa Prefecture**: • Takamatsu-shi, 15.V.1961, M. Chujo (1, KUM), JHL_ACITAI_051; • Miki-cho, 16.VI.1961, M.Chujo (1, KUM), JHL_ACITAI_050; • Kinuki-shi, 25.V.2015, J. Aoki (1, KUM), JHL_AREV_490; • Kita-gun, Hirai-cho, 13.V.1949, K. Iwata (1, ZMH), ZMH 837201; **Nagasaki Prefecture**: • Tsushima, Izuhara, 15.V.1971, H. Irie (1, KUM), JHL_ACITAI_006; **Nara Prefecture**: • Kasuga, 4.VI.1961, K. Ueda (1, KUM), JHL_ACITAI_049; **Oita Prefecture**: • Tsukahara, Yufuin-machi, Yufu-shi, 18.VI.2016, R. Ito, (1, KUM), JHL_AREV_779; **Osaka Prefecture**: • Mt. Iwawaki, 18.VI.1966, H. Kimura (1, KUM), JHL_ACITAI_048.

##### Diagnosis.

Body length 6.1–7.0 mm. Covered in pale brown, dark gray, and black scales, with a tan-colored, V-shaped, transverse band across middle of elytra. Pronotum lacking impunctate longitudinal midline (present in *A. koguma*). Scutellum bordered by pale brown scales, but conspicuously bare and shiny dorsally (Fig. 3E). Elytra with erect scales only on odd intervals. Sclerolepidia distinct and protruding (Fig. 4B). Third tarsomeres distinctly emarginate. Pedon with lateral edges approx. parallel in dorsal view, but converging evenly into a blunt, rounded point. Internal sac not visible inside aedeagus, but with a protruding basal structure that bears two hook-like structures anteriorly at the apex that are curved 90 degrees inward (Fig. 6L1–2).

##### Distribution.

This species occurs in Japan (mainland) and China, and has also been reported from South Korea (Park et al. 2009) and the Kuril Islands (Alonso-Zarazaga et al. 2023).

##### Remarks.

An examination of the holotype of *A. palliata* (NHMUK), the holotype of *A. dorsonigrita* (NHMB), a paratype of *A. dorsonigrita* (ZMH), as well as comparisons of the original descriptions of these two species revealed that *A. dorsonigrita* syn. nov. is a junior synonym of *A. palliata*. In particular, the holotypes of *A. palliata* and *A. dorsonigrita* (1) both have scutella which are bare dorsally, bordered by scales around the perimeter (2) are more than 5 mm in length (measured from the apex of the elytra to the base of the rostrum), and (3) bear the same characteristic pattern of scales dorsally. Furthermore, the original descriptions of these species certainly appear to refer to the same species, and notably the body length measurements given for *A. palliata* (“3 lin.” = 6.25 mm) and *A. dorsonigrita* (5–5.8 mm) are approximately equal. The much smaller species (at most 4.4 mm) previously referred to as *A. dorsonigrita* by Morimoto and Miyakawa (1995) is new and is described below as *A. squamata*.

#### ﻿ Acicnemis postica

Taxon classificationAnimaliaColeopteraCurculionidae

Hubenthal, 1919

C5483005-1F16-5B59-A4CF-C62B2EA1A456

Figs 1, 6M1–6, 2, 24

Acicnemis cruciata Kleine, 1924, syn. nov.

##### Type material examined

**(Japan: 31; Taiwan: 5; Total: 36). *Lectotype* (designated here): Taiwan** • “Formosa Is.”, “Coll. Pape”, SDEI Müncheberg Col – 18570 (1, SDEI), bears red label reading “Syntypus” and a new, red lectotype label reading “LECTOTYPE / *Acicnemis postica* / Hubenthal, 1919 / Det. Lewis & Kojima, 2025”. ***Paralectotypes*. Taiwan** • Fuhosho, “IX-7–09”, H. Sauter, SDEI Müncheberg Col – 18571 (1, SDEI), bears red label reading “Syntypus” and a new, yellow paralectotype label; • Kankau (Koshun), VI. 1912, H. Sauter, SDEI Müncheberg Col – 18572 (1, SDEI), bears red label reading “Syntypus” and a new, yellow paralectotype label.

**Figure 24.**
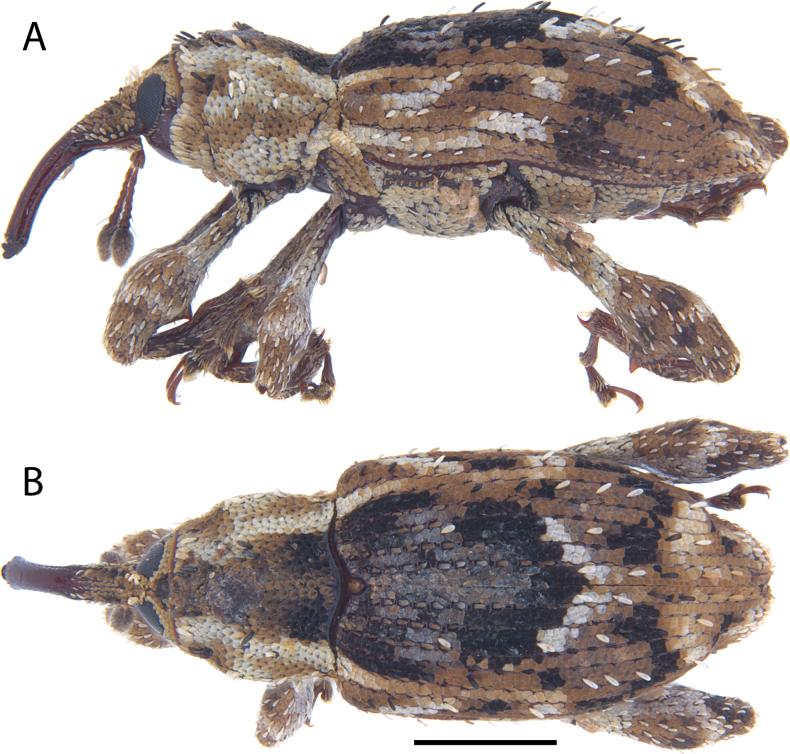
*Acicnemis postica* Hubenthal, 1919 (JHL_AREV_780). A. Lateral; B. Dorsal. Scale bar: 1 mm.

##### Other type material.

• Kosempo, “VII–19”, H. Sauter, *Acicnemis cruciata* holotype (1, SDEI), SDEIColeoptera #303422, SDEI Müncheberg Col – 18568.

##### Non-type material.

**Taiwan** • Taoyuan Hsein, Paling, 24.V.1977, H. Fujita (1, KUM), JHL_ACITAI_057; • Wenchuan Hsein, Lushan, 4.VII.1978, T. Mikage (1, KUM), JHL_ACITAI_058; • Ping Tung Hsein, Kenting, 2.IV.1965, S. Ueno (1, KUM), JHL_ACITAI_060; • Kenting, 31.III.1970, Y. Miyake (2 dissected males, KUM), JHL_ACITAI_082, JHL_SYN_179; • Chihpen Wenchuan, Taitung Hsien, 7. VI.1980, H. Makihara (1, KUM), JHL_AREV_735; • Jiuyuehtan, 31.V.1978, K. Kusama (1, KUM), JHL_AREV_736; • Taidong, Lijia road, 22.8062°N, 121.0310°E, 19.VIII.2013, V. Grebennikov (1, CNCI), CNCCOLVG00006901 (BOLD accession number: MEBLA191-14); **Japan: Kagoshima Prefecture**: • Shinokawa, Setouchi-cho, Amami Oshima, 10.VII.2008, K. Ozaki (1, CMIC), CBM ZI 136615; • Nakanoshima Island, 1–2.V.1975, H. Irie (1, KUM), JHL_AREV_750; • Nagata, Yakushima Island, 17.V.1960, J. Nagao (1, KUM), JHL_AREV_754; • Mt. Kindake, Nakano Is., 7–9.VI.2019, S. Imada, (1, KUM), JHL_AREV_780; **Okinawa Prefecture**: • Ishigaki Island, Mt. Omoto, 18–22.VI.1991, K. Morimoto (1, KUM), JHL_ACITAI_055; • Mt. Omoto, 21.V.1977, H. Irie (1, KUM), JHL_AREV_755; • Mt. Omoto, 18–21.IV.1975, H. Irie (1 dissected male, KUM), JHL_ACITAI_062; • Ushikumori, Iriomote Island, 7.X.1963, K. Morimoto (1, KUM), JHL_AREV_756; • Yonaguni Island, Urabu-dake, 25–26.VI.1974, T. Mikage (6, KUM), JHL_ACITAI_056, JHL_AREV_751 – JHL_AREV_753; • Okinawa Island, Hiji, 9.VII.1974, T. Mikage (2, KUM), JHL_ACITAI_059; • Fusato, Okinawa Island, 5.VI.1970 (1, KUM), JHL_AREV_762; • Omotodake, Ishigaki Island, 8.VII.1968, M. Takagi (1, KUM), JHL_AREV_761; • Funaura, Iriomote Island, 2.X.1978 (2, RUMC), JHL_AREV_709, JHL_AREV_710; • Banna, Ishigaki Island, 14.III.1964, S. Azuma (1, RUMC), JHL_AREV_712; • Haneji, Okinawa Island, 10.VIII.1979, T. Kohama (1, RUMC), JHL_AREV_711; • Mt. Inbi-take, Yonaguni Island, 2.VI.2021, S. Imada (1, KUM), JHL_AREV_723; • Kuburadake, Yonaguni Island, 15.VI.2001, L. Hirano (1, KUM), JHL_AREV_725; • Shirahama, Iriomote Island, 31.VIII–5.IX.1969, H. Makihara (9, KUM), JHL_AREV_726 – JHL_AREV_734; • Shirahama, Iriomote Island, 4.X.1963, S. Ueno (1, KUM), JHL_AREV_737; • upper Nakara, Iriomote Island, 12.III.1964, Y. Miyatake (1, KUM), JHL_AREV_738; • Komi, Iriomote Island, 12.VII.1963, Y. Miyatake (1, KUM), JHL_AREV_739; • Sonai, Yonaguni Island, 25–29.VIII.1969, H. Makihara (10, KUM), JHL_AREV_740 – JHL_AREV_749; • Okinawa Island (1, OIST), JHL_AREV_983 (GenBank accession number: PV255638); • Tancha, Okinawa Island, 18.V.2024, J.H. Lewis, sweeping vegetation (1, OIST), JHL_AREV_994.

##### Diagnosis.

Body length 3.2–5.2 mm. Covered in orange, white, dark and pale gray scales. Elytra with inner five or six intervals black and gray; outer intervals with pale brown to orange scales; with V-shaped band of white scales along middle three or four intervals in center of elytra. Pronotum lacking impunctate longitudinal midline (present in *A. koguma*). Scutellum small and covered in erect, hair-like yellow scales. Elytra with erect scales only on odd intervals. Sclerolepidia distinct and protruding. Third tarsomeres emarginate. Metatibia evenly curved, not sinuate as in most other species. Pedon with lateral edges approx. parallel in dorsal view, but converging evenly into a blunt, rounded point. Internal sac not visible inside aedeagus, but protruding and with two sclerotized cylinder-shaped structures posteriorly near the base (Fig. 6M1–2).

##### Distribution.

This species occurs commonly throughout southern Japan (Ryukyu Islands) and Taiwan.

##### Remarks.

Examination of the *A. cruciata* Kleine, 1924 syn. nov. holotype (SDEIColeoptera #303422) revealed that this is a junior synonym of *A. postica* as it possesses all of the key characteristics that define this distinctive species (same scale pattern, evenly curved metatibiae, small, scaled scutellum). We designate one of the Hubenthal *A. postica* syntypes (SDEI Müncheberg Col – 18570) as a lectotype to fix the identity of this species.

#### ﻿ Acicnemis ryukyuana

Taxon classificationAnimaliaColeopteraCurculionidae

Lewis, 2023

9B388526-9B1E-5202-BB6E-58F07231B225

Figs 1, 2F, 6N1–6, 2, 25, 32A, C

##### Type material examined

**(Japan: 28). *Holotype*. Japan: Okinawa Prefecture**: • Ishigaki, Omoto-dake, 2.VII.1991, K. Hidetada, bears black-bordered red label reading “HOLOTYPE / *Acicnemis ryukyuana* / Lewis, 2023”, JHL_ACI_004, dissected male, deposited in KUM. ***Paratypes*. Japan: Okinawa Prefecture**: • Ishigaki Island, Yarabu-dake, 28.V.1998, K. Takahashi (1, KUM), JHL_ACI_001; • Ishigaki Island, Omoto-dake, 2.VII.1991, K. Hidetada (9, KUM), JHL_ACI_002, JHL_ACI_003, JHL_ACI_005–JHL_ACI_011; • Ishigaki Island, Mt. Yarabudake, 17–20.IV.1998, S. Ohmomo (1, KUM), JHL_ACI_012; • Ishigaki Island, Omotodake, 30.VI.1974 (1, KUM), JHL_ACI_013; • Okinawa Island, Kunigami, Yambaru National Park, Yona Field (26.73972°N, 128.23630°E), 4–18. IX.2015, A. Miyagi, Y. Tamaki, I. Maehira, L. Iha, S. Iriyama, T. Yoshida (2, OIST), OKENT0063788, OKENT0063789 (dissected male); • Okinawa Island, Kunigami, Yambaru National Park, Yona Field (26.73972°N, 128.23630°E), 7–21.VIII.2015, M. Yoshimura, M. Ogasawara (1, OIST), OKENT0053190 (GenBank accession number: PV255639); • Okinawa Island, Kunigami, Yambaru National Park, Yona Field (26.73894°N, 128.23720°E), 21.VIII–4. IX.2015, Y. Tamaki, T. Yoshida (2, OIST), OKENT0055037, OKENT0053264; • Okinawa Island, Kunigami, Yambaru National Park, Yona Field (26.73972°N, 128.23630°E), 18.IX–16.X.2015, A. Miyagi, I. Maehira, T. Yoshida (1, OIST), OKENT 0053498 (GenBank accession number: PV255640); • Okinawa Island, Hiji, 8.VII.1974, T. Mikage (1, KUM), JHL_ACI_014.

**Figure 25.**
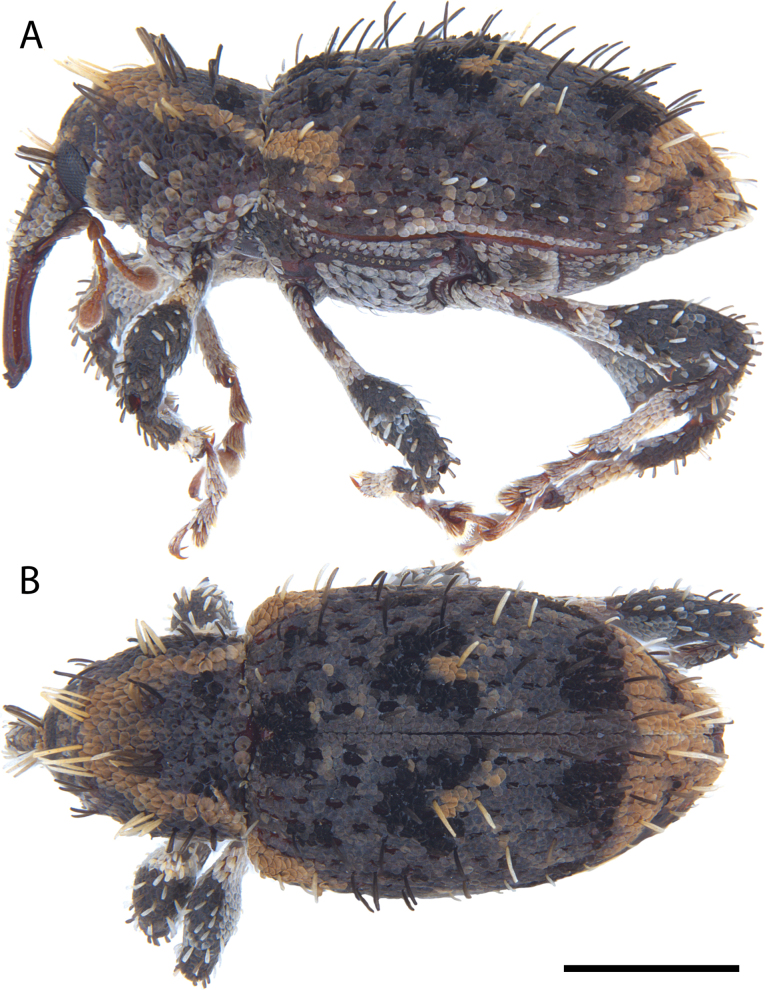
*Acicnemis ryukyuana* Lewis, 2023 (OKENT0063788). A. Lateral; B. Dorsal. Scale bar: 1 mm.

##### Non-type material.

**Japan: Okinawa Prefecture**: • Takeda-rindo, Ishigaki Island, 19–22.II.2007, H. Makihara & E. Kagaya (1, KUM), JHL_AREV_625.

##### Diagnosis.

Body length 3.2–5.0 mm. Covered in dark gray, black, and bright yellow scales, with a black-bordered, yellow spot at the center of each elytron. Apex of elytra covered in yellow scales, and contrasting with overall dark dorsal surface of elytra. Pronotum lacking impunctate longitudinal midline (present in *A. koguma*), but with an M-shaped band of yellow scales. Scutellum small, red, shiny, and largely bare over dorsal surface. Elytra with long, erect scales only on odd intervals. Sclerolepidia distinct and protruding. Third tarsomeres weakly emarginate to truncate (Fig. 2F). Pedon with lateral edges approx. parallel in dorsal view, but converging evenly into a blunt, rounded point. Internal sac not visible inside aedeagus, but with protruding basal structure that bears a distinctive U-shaped formation anteriorly at its apex (Fig. 6N1–2).

##### Distribution.

This species is known only from Okinawa Prefecture, Japan (Yambaru National Park (Okinawa Island) and forested regions of Ishigaki Island.

##### Remarks.

This species is very distinct from other Japanese *Acicnemis* and its range-restricted, endemic presence in pristine, subtropical rain forest dominated parts of Okinawa Prefecture was hitherto a mystery. However, preliminary morphological and molecular evidence suggest a sister relationship between *A. ryukyuana* and an undescribed Southeast Asian (Thai) species that possesses emarginate third tarsomeres. *Acicnemis ryukyuana* likely represents a lineage that was isolated from the undescribed Thai species lineage when the Ryukyu Islands separated from mainland Asia (see Osozawa et al. 2011) and evolved truncate third tarsomeres as derived character.

#### ﻿ Acicnemis sauteri

Taxon classificationAnimaliaColeopteraCurculionidae

Hubenthal, 1919

5D5F21B1-AD66-5E6F-8067-88A6CA815813

Figs 1, 6O1–6, 2, 26

##### Type material examined

**(Japan: 21; Taiwan: 18; Total: 39). *Lectotype* (designated here). Taiwan** • Hoozan, 1910, H. Sauter, bears a red label reading “Typus” and a new, red lectotype label reading “LECTOTYPE / *Acicnemis sauteri* / Hubenthal, 1919 / Det. Lewis & Kojima, 2025”, SNSD, JHL_ACITAI_004. ***Paralectotypes*. Taiwan** • Hoozan, “08–10”, Sauter (3, SDEI), bear a red label reading “Syntypus”, SDEI Muncheberg Col 18573, 18574, 18583; • Kosempo, Sauter (4, SDEI), bear a red label reading “Syntypus”, SDEI Muncheberg Col 18575, 18576, 18577, 18578; • Fuhosho, “VIII-09”, Sauter (3, SDEI), bear a red label reading “Syntypus”, SDEI Muncheberg Col 18580, 18581, 18582; • Taihorinsho, “X-09”, Sauter (1, SDEI), bears a red label reading “Syntypus”, SDEI Muncheberg Col 18579.

**Figure 26.**
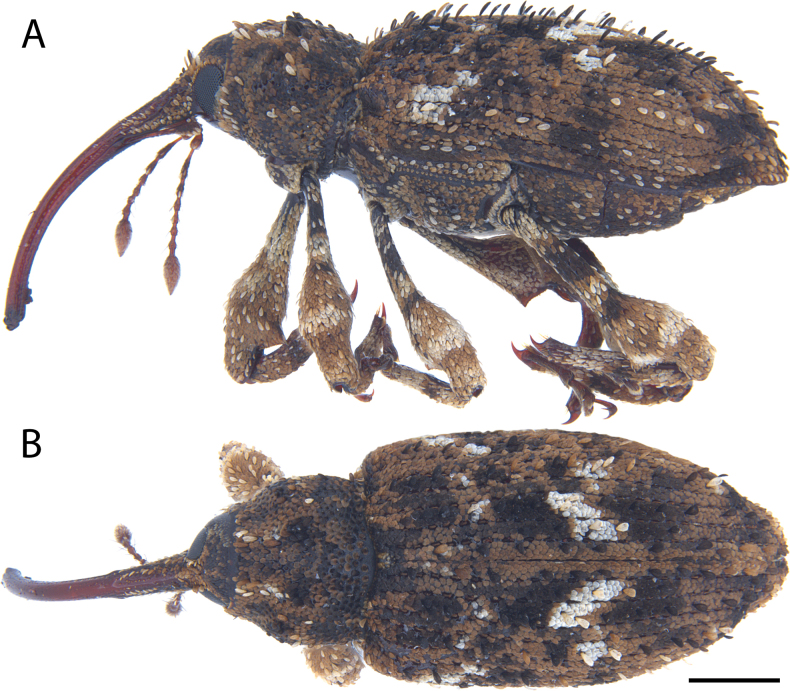
*Acicnemis sauteri* Hubenthal, 1919 (JHL_AREV_623). A. Lateral; B. Dorsal. Scale bar: 1 mm.

##### Non-type material.

**Taiwan** • Lushan Wenchuan, Nantou Hsien, 6–7.VI.1976, H. Makihara (2, KUM), JHL_AREV_609, JHL_AREV_611; • Sun Moon Lake, Nantou Hsien, 6.XII.2013, J. Aoki (10, KUM), JHL_SYN_148 (dissected male), JHL_SYN_149 (dissected male), JHL_AREV_613 – JHL_AREV_620; • Wukongshan, Liu-kuei, Kaohsiung Hsein, 2.V.1996, H. Takizawa (1, HUM), JHL_ACITAI_085; **Japan: Kagoshima Prefecture**: • Nakanoshima Island, 25–30.IV.1975, H. Irie (1, KUM), JHL_AREV_608; • Jinushi-daimyojin, Satomura, Nakano-jima Island, 7–9.VII.2019, S. Imada (1, KUM), JHL_AREV_610; • Sato, Nakano-jima Island, 7–9.VII.2019, N. Tsuji (3, KUM), JHL_AREV_691 – JHL_AREV_693; • Aden, Kikai-jima Island, 6.V.2022, R. Ito (3, KUM), JHL_AREV_623, JHL_AREV_908, JHL_AREV_909; • Kikai-jima Island, 4.III.2012, J. Aoki (1, KUM), JHL_AREV_695; • Torigamine, Amami-Oshima, 3.VIII.1976, N. Morishima (1, LBM), LBM 5123; • Hatsuno, Amami-Oshima, 24–30.VI.1973, M. Ito (1 dissected male, LBM), LBM 5132; • Sumiyo, Amami Oshima, 27.V.2004, K. Takahashi (1, KUM), JHL_AREV_694; • Akuseki-jima Island, Ue-shuraku, Sakamori-jinja, 2–6.VII.2019, S. Imada (1, KUM), JHL_DNA_121 (GenBank accession number: PV255642); • Nakano-jima Island, Satomura, Jinushi-daimyojin, 7–9.VII.2019, S. Imada (1, KUM), JHL_DNA_120 (GenBank accession number: PV255641).

##### Diagnosis.

Body length 4.9–6.2 mm. Covered in pale and dark brown scales, with two obliquely shaped white, black-bordered spots across middle of elytra. Pronotum lacking impunctate longitudinal midline (present in *A. koguma*). Scutellum small, bordered by pale yellow scales, and weakly bare antero-dorsally (compare with *A. kiotoensis* which possesses a large, broad, bare scutellum). Odd and 2^nd^ elytral intervals with erect scales (4^th^ elytral interval also occasionally with 1–2 scales near white spot). Sclerolepidia indistinct, forming low, rounded, non-protruding tubercles. Third tarsomeres emarginate (as opposed to truncate, as in *A. koguma*). Pedon with lateral edges approx. parallel in dorsal view, but converging in distal 1/2 evenly into a blunt point. Pedon elongate (pedon length / total aedeagus length including temo = 0.26). Internal sac protruding and unmodified in the posterior 1/2, but with roughened musculature in anterior 1/2 (Fig. 6O1–2).

##### Distribution.

This species occurs in southern Japan (Kagoshima Prefecture) and Taiwan, and has also been recorded from South Korea (Alonso-Zarazaga et al. 2023). The apparent lack of this species in the southern Ryukyu Islands (Okinawa Prefecture) is odd; however, similar patterns are known in other weevils (e.g., *Aphanerostethus magnus* Lewis & Kojima, 2024)

##### Remarks.

This species is closely allied with *A. maculaalba* (see Remarks for that species). We designate one of Hubenthal’s syntypes (SNSD, JHL_ACITAI_004) as a lectotype to fix the identity of this species.

#### ﻿ Acicnemis shibatai

Taxon classificationAnimaliaColeopteraCurculionidae

Voss, 1971

F6287459-457D-5125-A8A1-2D3A16A2CFE4

Figs 6P1–6, 2, 27

##### Type material examined

**(Japan: 17; South Korea: 1; Taiwan: 2; Total: 20). *Holotype*. Japan: Kagoshima Prefecture**: • Ikari, Amami-Oshima, 28.V.1960, T. Shibata, KUM, bears red label that reads “Holotype”. ***Paratypes*. Japan: Kagoshima Prefecture**: • Ikari, Amami-Oshima, 6.V.–4.VI.1960, T. Shibata, JHL_AREV_444 – JHL_AREV_451, JHL_ACITAI_064 (9, KUM), bear red label that reads “Paratype”.

**Figure 27.**
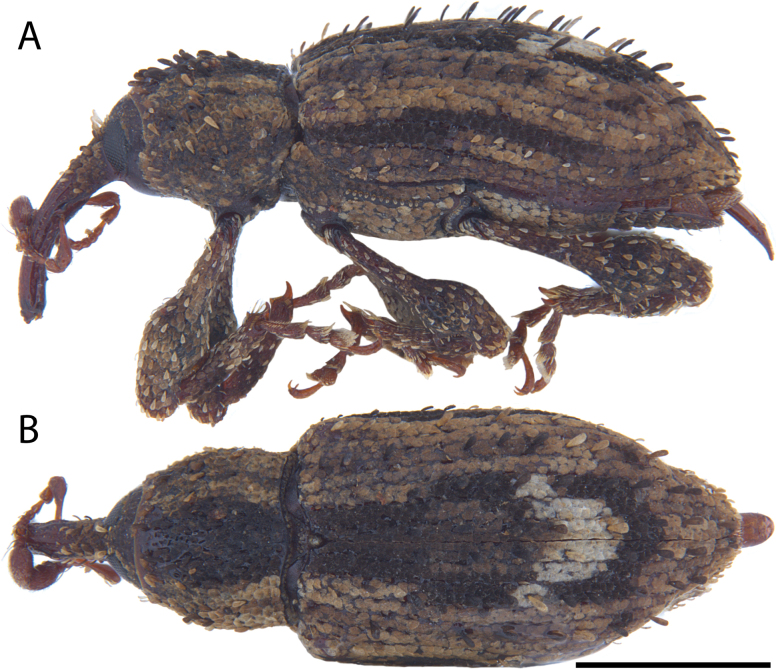
*Acicnemis shibatai* Voss, 1971 (JHL_AREV_776). A. Lateral; B. Dorsal. Scale bar: 1 mm.

##### Non-type material.

**Japan: Chiba Prefecture**: • Mt. Kiyosumi, 9.VI.1963, K. Morimoto (1, KUM), JHL_AREV_459; • Mt. Kiyosumi, 14.VI.1992, T. Ito (1, CMIC), CBM ZI 63020; **Kagoshima Prefecture**: • Yuwan, Amami-Oshima, 31.VII.1963, Y. Hirashima (1, KUM), JHL_AREV_457; • Mt. Yuwan, Amami-Oshima, 30.VII.1963, L. Gressitt (1 dissected male, KUM), JHL_ACITAI_070; • Ishihara-rindo, 9–11.VI.1978, R. Noda (1, KUM), JHL_AREV_458; • Mikyo, Tokunoshima, 27.VII.1963, Y. Hirashima (1, KUM), JHL_AREV_453; • Nishinakama, Amami-Oshima, 2.VI.1970, H. Makihara, (1, KUM), JHL_AREV_776; **Okinawa Prefecture**: • Shirahama, Iriomote Island, 31.VIII–5.IX.1969, H. Makihara (1, KUM), JHL_AREV_452; **South Korea** • Kwangnung, Pochon-gun, Kyonggi-do, 16–19.VII.1992, K. Morimoto (1, KUM), JHL_AREV_419; **Taiwan** • Wulai, 27.V.1965, K. Morimoto (2, KUM), JHL_AREV_455, JHL_AREV_456.

##### Diagnosis.

Body length 3.0–3.8 mm. Covered in dark and pale brown, white, and black scales, with a white-scaled, V-shaped band in the center of the elytron. Pronotum lacking distinct longitudinal, impunctate midline (present in *A. koguma*). Scutellum covered in pale scales. Elytra with erect scales only on odd intervals. Sclerolepidia distinct and protruding. Third tarsomeres emarginate. Pedon tube-shaped with lateral edges approx. parallel in dorsal view, but sinuating evenly into a blunt, rounded point. Internal sac with a small sclerotized structure posteriorly, and two prong-like structures anteriorly at its apex (Fig. 6P1–2).

##### Distribution.

This species occurs in Japan (mainland, Ryukyu Islands south to Amami-Oshima), South Korea, and Taiwan.

#### ﻿ Acicnemis shigematsui

Taxon classificationAnimaliaColeopteraCurculionidae

Morimoto & Miyakawa, 1995

B87BD522-0EA3-561B-A8C4-3A55BE18EDB3

Figs 3F, 6Q1–6, 2, 28

##### Type material examined

**(Japan: 9). *Holotype*. Japan: Okinawa Prefecture**: • Ishigaki Island, Omoto-dake, 1.VIII.1989, K. Shigematsu, bears red label reading “(HOLOTYPE) Acicnemis shigematsui Morimoto et. Miyakawa, 1994”, JHL_ACITAI_052, ELKU, ELKU 2960. ***Paratypes*. Okinawa Prefecture**: • Iriomote Island, Shirahama, 31.VIII–5.IX.1969, H. Makihara (2, KUM), JHL_ACITAI_005 (dissected male), JHL_ACITAI_047; • Iriomote Island, Mt. Ushiku, 4.XI.1963, G. A. Samuelson (1, KUM), JHL_ACITAI_053; • Iriomote Island, Upper Nakara River, 12.III.1964, Y. Miyatake (1, KUM), JHL_ACITAI_045.

**Figure 28.**
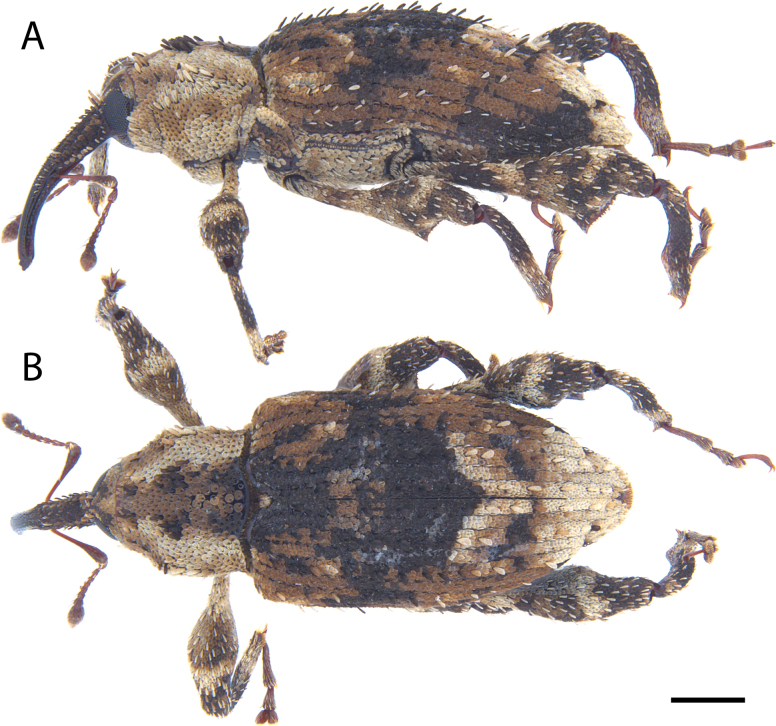
*Acicnemis shigematsui* Morimoto & Miyakawa, 1995 (JHL_ACITAI_052). A. Lateral; B. Dorsal. Scale bar: 1 mm.

##### Non-type material.

**Japan: Okinawa Prefecture**: • Ishigaki Island, Yarabu-dake, 3.V.1998, K. Takahashi (1, KUM), JHL_ACITAI_054; • Omoto-dake, 1–4.VII.2009, R. Ito (1, KUM), JHL_AREV_481; • Omoto-dake, 29.III.2022, T. Nozaki (1, KUM), JHL_AREV_482; • Okinawa Island, Naha, Shuri, “Sumiyoshi Park” (probably referring to Sueyoshi Park), 23.XI.1990, K. Morimoto (1, KUM), JHL_ACITAI_044.

##### Diagnosis.

Body length 5.1–7.0 mm. Covered in pale brown, dark gray, and black scales, with a tan-colored, V-shaped, transverse band across middle of elytra. Pronotum lacking distinct longitudinal, impunctate midline (present in *A. koguma*). Scutellum covered in pale scales (Fig. 3F). Elytra with erect scales only on odd intervals. Sclerolepidia distinct and protruding. Third tarsomeres distinctly emarginate. Pedon with lateral edges approx. parallel in dorsal view, but weakly sinuating into a blunt, subtriangular point. Internal sac not visible inside aedeagus, but with a protruding basal structure extending to the end of the temo and tuberculate (roughened) musculature anteriorly at the apex (Fig. 6Q1–2).

##### Distribution.

This species is endemic to Okinawa Prefecture, Japan, and is currently only known from Ishigaki, Iriomote, and apparently also Okinawa Island.

##### Remarks.

This species is quite similar in size and color to *A. palliata*; however, it was differentiated by Morimoto and Miyakawa (1995) on the basis of elytral color differences alone. To verify the validity of this species the type series of *A. shigematsui* and *A. palliata* were compared, and several significant differences in external and genital morphology were discovered which are all newly reported in the dichotomous key below. Furthermore, although *A. shigematsui* was previously only known from Ishigaki and Iriomote Islands, a specimen deposited in the KUM was collected on Okinawa Island (400 km away from the type localities). No specimens of *A. shigematsui* have since been collected on the island. Further field studies are required to ascertain whether this rarely collected species is truly established on the island.

#### ﻿ Acicnemis

Taxon classificationAnimaliaColeopteraCurculionidae

sp. 1

F14219CE-481D-553D-900D-14BA7FAE6024

Figs 6R1–6, 2, 29

##### Material examined

**(Myanmar: 2; Taiwan: 7; Thailand: 3; Vietnam: 2; Total: 14). Myanmar** • Tamu Village, Kachin State, 7.VII.2003, A. Abe (2, KUM), JHL_SYN_169 (dissected male), JHL_AREV_717; **Taiwan** • “Formosa”, T. Kano (1, NMNS), JHL_AREV_681; • His-Leng, 5.V.1981, S. Tsuyuki (5, TUA), JHL_SYN_175 (dissected male), JHL_SYN_176 (dissected male), JHL_AREV_714 – JHL_AREV_716; • Lushan Wenchuan, Nantou Hsien, 6.VI.1976, H. Makihara (1, KUM), JHL_AREV_719; **Thailand** • Pupin-Meo, 11.X.1993, T. Senoh (1 dissected male, KUM), JHL_SYN_170; • Chiang Rai, Wiang Pa Pao, 12.XI.2012, K. Takahashi (2, KUM), JHL_AREV_718 (EGP0160-H9), JHL_AREV_722; • Doi Suthep, 23.VI.1993, T. Senoh, (1, KUM), JHL_AREV_766; **Vietnam** • Phia Oac National Park near Phia Den, Cao Bang Province, 22°32'20"N, 105°51'57"E, 19–22.III.2012, S.D. Gaimari, M. Hauser, H.T. Pham (2, CMNC), JHL_AREV_703, JHL_AREV_704; • “IIea” Binh, Tonkin, bears red label reading “*simpliciata* Typus H.”, bears brown label reading “*A. simpliciata* m ♂ Heller det. 1930” (1, SNSD), JHL_ACITAI_093.

**Figure 29.**
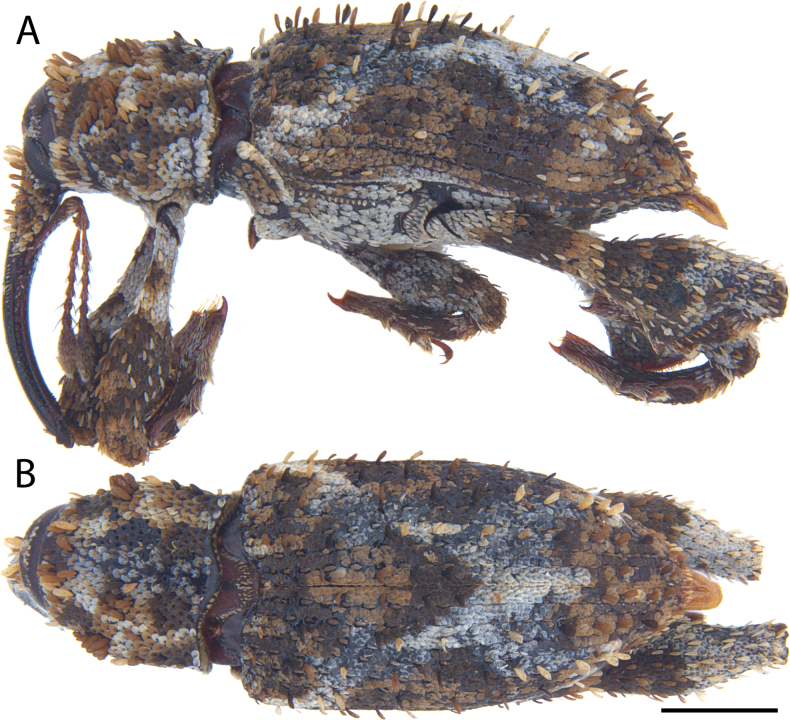
*Acicnemis* sp. 1 (JHL_AREV_766). A. Lateral; B. Dorsal. Scale bar: 1 mm.

##### Diagnosis.

Body length 4.1–5.0 mm. Covered in pale and dark brown scales, with bright X-shaped band of white scales across apical 1/2 of elytra, which extends up to the elytra humeri. Distinctive bright white scale bands also present on hind femora. Pronotum lacking distinct longitudinal, impunctate midline (present in *A. koguma*). Scutellum small, bordered by pale, yellow scales, and weakly bare dorsally (or covered in scales); compare with *A. kiotoensis* which possesses a large, broad, bare scutellum. Only odd elytral intervals with broad, erect scales. Sclerolepidia numerous, dense, and nub-like, protruding laterally outward (unlike *A. maculaalba*, *A. kiotoensis*, and *A. koguma* which have indistinct, low, rounded sclerolepidia). Third tarsomeres emarginate (as opposed to truncate, as in *A. koguma*). Pedon with lateral edges approx. parallel in dorsal view, but sinuating in distal 1/2 into a distinct point. Internal sac protruding and clear across posterior 2/3; with two bean-shaped and U-shaped sclerotized structure anteriorly at apex (Fig. 6R1–2).

##### Distribution.

This widely distributed species occurs in Vietnam, Thailand, Myanmar, and Taiwan.

##### Remarks.

One of K.M. Heller’s specimen of this species in the SNSD collection has a holotype label with the name “*A. simpliciata*” written (JHL_ACITAI_093); however, we were unable to find a formal description of this species in any of Heller’s works. Heller often attached type labels to specimens without formally publishing the descriptions (Olaf Jäger – SNSD Collection Manager to JHL, pers. comm. 2025; Katsura Morimoto – KUM Curator to HK pers. comm., 2025). Thus, this species is likely undescribed; however, there are a number of other similar Southeast Asian species for which type material should be compared with *Acicnemis* sp. 1 before formal description. This falls outside of the scope of the current work (study area); however, we still acknowledge the presence of this species in Taiwan and treat it here as an unnamed species.

#### ﻿ Acicnemis squamata

Taxon classificationAnimaliaColeopteraCurculionidae

Lewis & Kojima sp. nov.

DE46B977-D138-5A19-A1AF-1AE7EC20886C

https://zoobank.org/E5C566CF-28A3-4D1B-918D-4FE0D42B0B50

Figs 1, 6S1–6, 2, 30

##### Type material

**(Japan: 46). *Holotype*. Japan: Tokushima Prefecture**: • Mt. Tsurugi, Awa, 2.VI.1957, S. Ueno, JHL_ACITAI_068, dissected male, deposited in KUM. ***Paratypes*. Japan: Aomori Prefecture**: • Oyama-nagane, Nakadomari-machi, Kitatsugaru-gun, 7.VI.2010, K. Morimoto (1, KUM), JHL_AREV_702; **Fukui Prefecture**: • Kinome-toge, 25.VI.1982, K. Morimoto (1, KUM), JHL_ACITAI_065; **Fukushima Prefecture**: • Yunohana, south Aizu, 8.VIII.1949, K. Nagayama (1, HUM), JHL_AREV_474; • Yunohana, south Aizu, 7.VI.1981, Y. Kurosawa (1, NMNS), JHL_AREV_682; • Yunohana, south Aizu, 20.VII.1974, Y. Kurosawa (2, NMNS), JHL_AREV_683; • Masuzawa Valley, Tateiwa-mura, 6.VI.2005, K. Morimoto (1, KUM), JHL_AREV_698; • Odayama Mt., Monden V., Kita-Aizu, 22.VI.1948, Y. Kurosawa (1 dissected male, NMNS), JHL_AREV_107; **Gifu Prefecture**: • unknown locality, 24.V.1949 (1, NHMB), JHL_ACITAI_077; • Hinaga, 22.VII.1946 (1, NHMB), JHL_ACITAI_078; • Hida, 1.VI.1957, T. Nakane (1, HUM), JHL_AREV_472; • Kawai-Idani, Hida, 1–2.VI.1957, T. Nakane (3, HUM), JHL_AREV_476 – JHL_AREV_478; **Gunma Prefecture**: • Osawa, Katashina, 5.VI.1988, T. Shinbori (1, KPMNH), JHL_AREV_684; • Numata, 30.V.1953, T. Takei (1, ZMH), ZMH 837203; • **Hokkaido**: Sapporo, “Takano” & H. Kono (2, HUM), JHL_AREV_462, JHL_AREV_465; • Rausu Shiretoko, 8.VII.1958, T. Nakane (2, HUM), JHL_AREV_475; • Chitose, 1.VIII.2011, J. Aoki (1, KUM), JHL_AREV_699; **Ibaraki Prefecture**: • Mt. Tsukuba, 11.VIII.1989, M.J. Sharkey (2, CMNC), JHL_AREV_3000, JHL_AREV_3001; **Ishikawa Prefecture**: • Yamanaka, Kaga, 20.VII.1910, T. Otsuka (1, HUM), JHL_AREV_463; **Iwate Prefecture**: • Shizukuishi, 7.VI.2010, Aoki Junichi (1, KUM), JHL_ACITAI_072; **Kanagawa Prefecture**: • Kuno, Odawara, 8.VI.2014, K. Watanabe (3, OIST), JHL_AREV_687 – JHL_AREV_689; • Mt. Ono, Yamakita, 10.V.1988, N.Niwa, (1, KUM), JHL_AREV_771; • **Kochi Prefecture**: • Engyoji, 22.VII.1952, Y. Wada (1, KUM), JHL_ACITAI_066; • **Kumamoto Prefecture**: • Mt. Hakucho, 14.V.1983, M. Chujo (1, KUM), JHL_ACITAI_063; • **Kyoto Prefecture**: • Miyama, Kyoto University Research Forest, 31.V.2010, Junichi Aoki (1, KUM), JHL_ACITAI_069 (GenBank accession number: PV255643); **Nagano Prefecture**: • Kaida Highlands, Kiso, 5.IX.2009, J. Aoki (1, KUM), JHL_AREV_696; • “Noziri Hotel”, 29.V.1941, T. Nakane (1, HUM), JHL_AREV_471; **Nara Prefecture**: • Mt. Omine, 23.V.1974, T. Hattori (1, KUM), JHL_AREV_467; **Niigata Prefecture**: • Mt. Yakemine, 3.VII.1949, H. Koike (1, KUM), JHL_ACITAI_061; • Kurokawa, 28.V.1955, K. Baba (1, ZMH), ZMH 837202; **Okayama Prefecture**: • Tsuki, Soja, 18.IV.1976, T. Aono (1, HUM), JHL_AREV_469; **Osaka Prefecture**: • Minoh, 30.V.1940, K. Taniguchi (1, HUM), JHL_AREV_470; **Shizuoka Prefecture**: • Kakegawa, Ijiri, 3.V.2010, J. Aoki (1, KUM), JHL_ACITAI_073; • Kawanehon-machi, 28.VIII.2009, J. Aoki (1, KUM), JHL_AREV_697; **Tokushima Prefecture**: • Mt. Tsurugi, 27.VII.1961, M.T. Chujo (1, KUM), JHL_ACITAI_067; **Tochigi Prefecture**: • Nikko-shi, Chugushi, Ryuzuno-taki Fall, 19.VI.2014, S. Yamamoto (1, KUM), JHL_ACITAI_076 (GenBank accession number: PV255644); • Ashio, Funeishi-toge, 2.VII.2006, K. Takahashi (1, KUM), JHL_AREV_701; • **Tokyo Metropolis**: Niijima Island, Mt. Mukoyama, 15.V.1979, H. Fujita (1, KUM), JHL_ACITAI_071; • Kogezawa, “1954.5.10” (1 dissected male, KUM), JHL_ACITAI_009; • Tokura, 6.VIII.1950, S. Ueno (2, HUM), JHL_AREV_468; • Mt. Takao, 14.IV.1985, A. Tanaka (1 dissected male, HUM), JHL_AREV_106; **Wakayama Prefecture**: • Mt. Kohya, VII.1949, M. Hayashi (1, HUM), JHL_AREV_473; **Yamagata Prefecture**: • Yonezawa, 22.VI.1948, Y. Kurosawa (1, HUM), JHL_AREV_464; • Naderayama, 23.V.1943, Y. Kurosawa (1, NMNS), NSMT-I-C 07303; • Seki, near Yonezawa, 2.VII.1944, Y. Kurosawa (1, NMNS), NSMT-I-C 07304; **Yamanashi Prefecture**: • Masutomi, 13–14.VI.1970, H. Takizawa (1, HUM), JHL_AREV_466; • Masutomi, “10.VI.2603” (1, NMNS), NSMT-I-C 07305; • Akeno, 6.VI.2010, T. Shinbori (2, KPMNH), JHL_AREV_685, JHL_AREV_686; • Kami-yoshida, Fuji-yoshida city, 15.VII.2024, J.H. Lewis (1, OIST), JHL_AREV_986.

**Figure 30.**
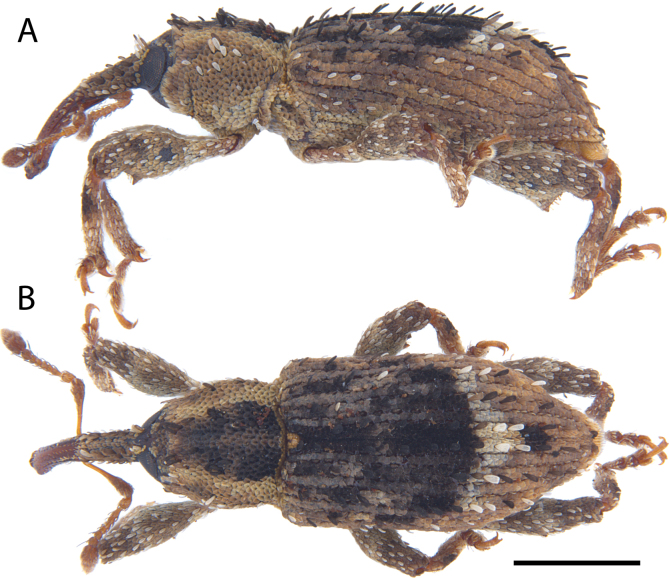
*Acicnemis squamata* Lewis & Kojima, sp. nov. (JHL_AREV_771). A. Lateral; B. Dorsal. Scale bar: 1 mm.

##### Diagnosis.

Body length 3.0–4.4 mm. Covered in pale yellow, white, gray, and black scales. Pronotum with central longitudinal band of dark gray to black scales, bordered laterally by pale brown scales. Elytra with first intervals alternating black (at base of scutellum) and yellow; second to sixth intervals gray in posterior 1/2 of elytra, humeri with yellow to pale scales; apical 1/2 of elytra with transverse white V-shaped band extending from first to fifth intervals; apex of elytra largely with pale to yellow scales. Pronotum lacking impunctate longitudinal midline (present in *A. koguma*). Scutellum covered in bright yellow to dull gray scales. Only odd elytral intervals with erect scales. Sclerolepidia numerous, dense, and nub-like, protruding laterally outward. Third tarsomeres emarginate. Apex of pedon with distinct sinuation and pointed (Fig. 6S1–2). Internal sac of aedeagus distinctly scaly in appearance, protruding, and not extending to midpoint of temo; anterior apex of internal sac ending in two scaly feather-like tips (Fig. 6S1–2).

##### Distribution.

This species is only known from mainland Japan, from Hokkaido south to Kyushu (Kumamoto Prefecture).

##### Etymology.

The specific name *squamata* is a reference to the diagnostic internal sac of the aedeagus which bears scaly patterning similar to snakeskin.

##### Remarks.

See *A. palliata* Remarks section for taxonomic history. *Acicnemis squamata* differs from *A. palliata* (= *A. dorsonigrita*) in size (*A. squamata* is 3.0–4.4 mm in length whereas *A. palliata* is 6.1–7.0 mm in size), genitalia (*A. squamata* with internal sac of aedeagus scaly in appearance, protruding, and ending in two scaly featherlike tips; *A. palliata* without scaly internal sac, and with two protruding hook-like structures curved inwards at 90°) and scutellum scaling (*A. squamata* with scutellum covered in scales; *A. palliata* with scutellum prominently bare along the dorsal surface). See also the dichotomous key below.

#### ﻿ Acicnemis suturalis

Taxon classificationAnimaliaColeopteraCurculionidae

Roelofs, 1875

CDF7E382-0987-565C-A937-4527DCFB4285

Figs 6T1–6, 2, 31

##### Type material examined

**(Japan: 24). *Holotype*. Japan: Hyogo Prefecture**: • Kobe, “1910–320”, G. Lewis, NHMUK015026634, bears a circular, white and red label reading “Type H.T.”.

##### Non-type material.

**Japan: Aomori Prefecture**: • Nei, Misawa-shi, 8.VI.2010, K. Morimoto (1, KUM), JHL_AREV_507; **Chiba Prefecture**: • Nagara, Chosei-gun, 12.VI.1988, M. Satoshi (2, CMIC), CBM ZI 159815, 159816; **Gunma Prefecture**: • Minakami, 6–7.VI.2008, K. Morimoto (6, KUM), JHL_AREV_504, JHL_AREV_505; **Ehime Prefecture**: • Mt. Takanawa, Matsuyama-shi, 11–12.VIII.2015, K. Kuroda (1, KUM), JHL_AREV_506; **Fukuoka Prefecture**: • Mt. Wakasugi (Chikuzen), 23.V.1954, K. Morimoto, JHL_AREV_422, JHL_AREV_423, JHL_ACITAI_092 (dissected male) (3, KUM); • Mt. Hiko, 15.VII.1955, H. Kamiya, JHL_AREV_424; **Hyogo Prefecture**: • Kobe, Mt. Maya, 3.IX.1963, K. Morimoto, JHL_AREV_421 (1, KUM); **Kanagawa Prefecture**: • Mimasutoge, 6.V.1990, H. Takizawa (1, HUM), JHL_AREV_632; • Hatano, 7.VI.1983, H. Takizawa (1, HUM), JHL_AREV_633; **Kyoto Prefecture**: • Katsura-gawa, 12.VI.1918, M. Suzuki, JHL_AREV_425 (1, KUM); • Kyoto University Ashu Forest, Miyama-cho, Tanann City, 23.V.2009, K. Morimoto (1, KUM), JHL_AREV_503; **Tochigi Prefecture**: • Nanma, Kanuma, 4.V.1993, H. Takizawa (1, HUM), JHL_AREV_631; **Niigata Prefecture**: • Kamikawa-cho, 3.VI.2000, K. Takahashi (2, KUM), JHL_AREV_508.

**Figure 31.**
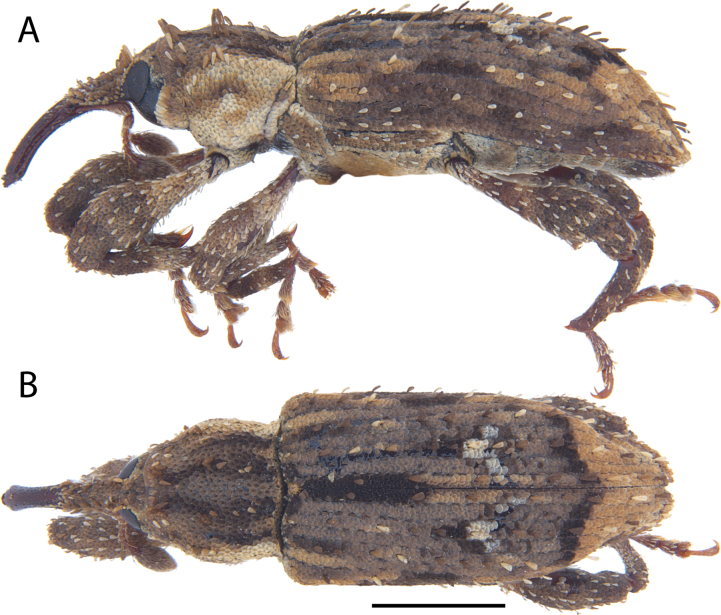
*Acicnemis suturalis* Roelofs, 1875 (JHL_AREV_421). A. Lateral; B. Dorsal. Scale bar: 1 mm.

**Figure 32.**
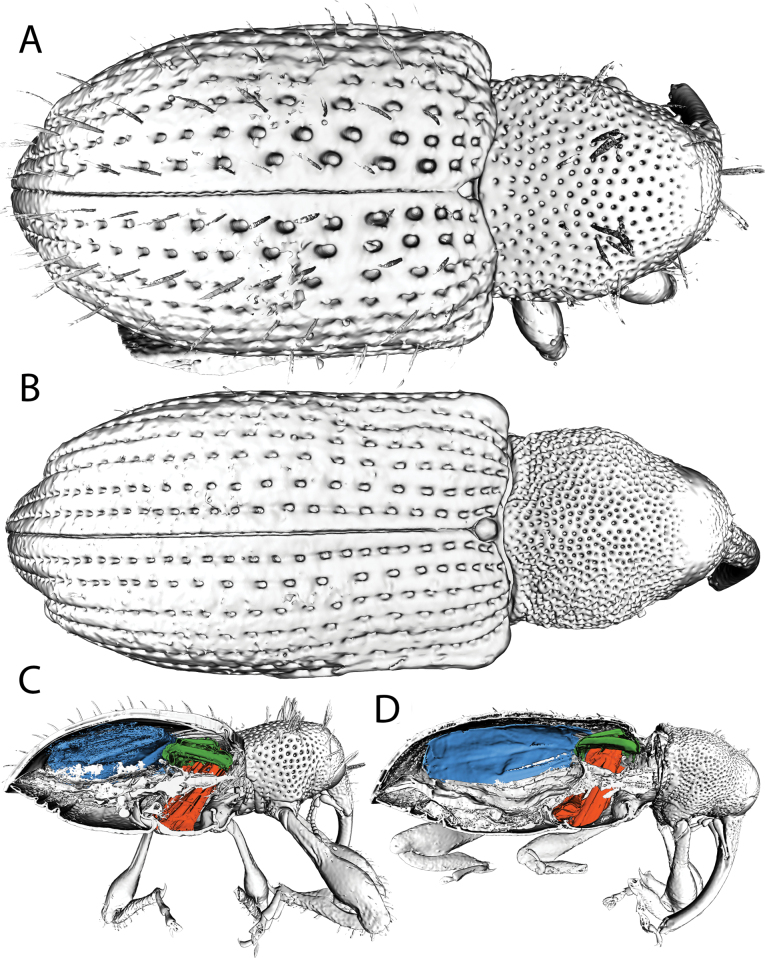
X-ray μCT generated 3D models of two *Acicnemis* species. The colored parts in the segmented models below represent hindwing (in blue), metanotum + dorso-longitudinal flight muscles (in green), and dorso-ventral flight muscles (in orange). A. *A. ryukyuana* Lewis, 2023 (OKENT0053498) dorsal view with scales removed, showing a relatively smooth pronotum and distantly spaced punctures; B. *A. albofasciata* (Ter-Minasian, 1953) (JHL_AREV_782) dorsal view with scales removed, showing a moderately rugose to roughened pronotum with interspaces between punctures much smaller; C. *A. ryukyuana* segmented model with the right elytron removed, showing a comparively larger metanotum and dorso-longitudinal flight muscles relative to the elytra (~ 30%); D. *A. albofasciata* showing a comparatively smaller metanotum and dorso-longitudinal flight muscles relative to the elytra (~ 23%).

##### Diagnosis.

Body length 4.0–4.7 mm. Covered in pale brown, dark and pale gray, pale yellow, and black scales, with two small white spots on the elytra (one on each side) in the apical 1/2 of the elytra. Pronotum lacking impunctate longitudinal carina (present in *A. koguma*). Scutellum covered in pale scales. Elytra with erect scales only on odd intervals. Sclerolepidia distinct and protruding. Third tarsomeres distinctly emarginate. Pedon with lateral edges converging in dorsal view, sinuating into an elongate, tapered tip. Internal sac protruding from aedeagus, and with two cylinder-shaped sclerotized structures anteriorly at the apex (Fig. 6T1–2).

##### Distribution.

This distinctive species occurs in Japan (mainland), Taiwan, and has also been reported from South Korea and the Kuril Islands (Russia) (Park et al. 2009; Alonso-Zarazaga et al. 2023).

### ﻿Key to the *Acicnemis* of East Asia

**Table:** 

1	Third tarsomeres truncate or weakly emarginate (Fig. 2E, F)	**2**
–	Third tarsomeres strongly emarginate (Fig. 2G)	**5**
2	Second and fourth elytral intervals with erect scales	***A. luteomaculata* Morimoto & Miyakawa, 1995**
–	Second and fourth elytral intervals without erect scales (may have small erect scales at elytral apex)	**3**
3	Erect scales on odd elytral intervals elongate, hair-like (distinctly longer than antennal club)	***A. ryukyuana* Lewis, 2023**
–	Erect scales on odd elytral intervals stubby, round (shorter than antennal club)	**4**
4	Sclerolepidia inconspicuous, low and rounded; third tarsomeres truncate; scales covering body predominantly brown	***A. koguma* Lewis & Kojima, sp. nov.**
–	Sclerolepidia distinct and protruding; third tarsomeres weakly emarginate; scales covering body predominantly gray	***A. biarcuata* Hubenthal, 1919**
5	Second elytral interval with erect scales	**6**
–	Second elytral interval lacking erect scales (may have small erect scales at elytral apex)	**8**
6	Scutellum distinctly large, wide, and lacking scales (Fig. 3B)	***A* . *kiotoensis* Nakane, 1963**
–	Scutellum slender and largely covered in scales dorsally (e.g., Fig. 3D)	**7**
7	Internal sac of aedeagus protruding but with roughened musculature proximal to pedon (Fig. 6I1–2); pedon stout (pedon length / total aedeagus length including temo = 0.2); fourth elytral interval usually with 2 or more erect scales around elytral center	***A. maculaalba* Roelofs, 1875 / *A. cordata* Hubenthal, 1919**
–	Internal sac of aedeagus protruding but with roughened musculature located at distance from pedon (Fig. 6O1–2); pedon elongate (pedon length / total aedeagus length including temo = 0.26); fourth elytral interval usually only with 1 erect scales around elytral center	***A. sauteri* Hubenthal, 1919**
8	Even-numbered elytral intervals costate	***A. costulifera* Hubenthal, 1919**
–	Elytral intervals not costate	**9**
9	Posterior edge of prosternum with prominent projections contiguous with the fore-coxae (Fig. 2B)	***A. laeta* Hubenthal, 1919**
–	Posterior edge of prosternum lacking prominent projections contiguous with the fore-coxae (e.g., Fig. 2A)	**10**
10	Cuticle distinctly naked (lacking scales) across much of body; pronotum roughly sculptured and carinate	***A. bickhardti* Hubenthal, 1919**
–	Cuticle largely covered in scales (sometimes specimens are denuded); pronotum punctate but not roughly sculptured or carinate (except at base of pronotum in *A. azumai*)	**11**
11	Pronotum with two exposed, bare, red tubercles medially at the base (Fig. 3A)	***A. azumai* Morimoto & Miyakawa, 1995**
–	Pronotum lacking tubercles at base (Fig. 3B–F)	**12**
12	Scutellum distinctly shiny and bare dorsally (Fig. 3E); sclerolepidia protruding, elongate, and numerous (Fig. 4B) ; dorsum largely covered in dark gray scales, with pale brown V-shaped, transverse band across elytra ; internal sac of aedeagus with 2 hook-like structures anteriorly at the apex, curved 90° inward (as in Fig. 6L1–2); larger species (6–7 mm in length)	***A. palliata* Pascoe, 1872**
–	Not with the above combination of characters	**13**
13	Pedon distinctly tapered apically into a fine point (Fig. 6T1–2); small (4.0–4.7 mm) gray-scaled species; first elytral interval covered in black scales in anterior 1/2; 2 white-scaled spots on each side of elytra medially; elytral apex with V-shaped black band followed by yellow scaled region	***A. suturalis* Roelofs, 1875**
–	Not with the above combination of characters	**14**
14	Small species (3.0–3.8 mm) with brown scaling and distinct white scaled V-shaped band in posterior 1/2 of elytra; pedon tube-shaped and internal sac with a small, sclerotized structure basally, and 2 prong-like structures anteriorly at its apex (Fig. 6P1–2)	***A. shibatai* Voss, 1971**
–	Size and color variable ; aedeagus not as above	**15**
15	Small species (3.0–4.4 mm) with pale and dark brown scales, and shallow, white-scaled, V-shaped band in posterior 1/2 of elytra; internal sac of aedeagus distinctly scaly in appearance and protruding; apex of internal sac ending in two scaly feather-like tips; apex of pedon with distinct sinuation and pointed (Fig. 6S1–2)	***A. squamata* Lewis & Kojima, sp. nov.**
–	Size and color variable; aedeagus not as above	**16**
16	Large species (5.1–7.0 mm) with pale brown, dark gray, and black scales, and a tan-colored, V-shaped, transverse band across middle of elytra; scutellum covered in erect scales (Fig. 3F) (unlike the somewhat similar *A. palliata*); pedon with lateral edges approx. parallel in dorsal view, but weakly sinuating into a blunt, subtriangular point ; internal sac not visible inside aedeagus, but with protruding basal structure extending to the end of the temo and with tuberculate (roughened) musculature at the apex (Fig. 6Q1–2) ; apparently endemic to Okinawa Prefecture (Ishigaki and Iriomote Islands)	***A. shigematsui* Morimoto & Miyakawa, 1995**
–	Size and color variable; aedeagus not as above	**17**
17	Exceptionally small species (2.1–3.0 mm), covered in pale brown, white, and gray scales, with 2 white-scaled spots in the apical 1/2 of the elytra; pedon with lateral edges approx. parallel in dorsal view, but converging evenly into a blunt, rounded point; internal sac without tuberculate or roughened musculature inside the aedeagus, but with a non-hooked protruding structure (unmodified in posterior 2/3, sclerotized in anterior 1/3) that reaches the tip of the temo (Fig. 6E1–2)	***A. exilis* Morimoto & Miyakawa, 1995**
–	Larger species (3.2–7.0 mm), variable in color; aedeagus not as above	**18**
18	Elytra with distinct white-scaled, X-shaped band across elytra (extremely bright in some specimens); pedon with lateral edges approx. parallel in dorsal view, but sinuating in distal 1/2 into a distinct point; internal sac protruding and clear across basal 2/3; with two bean-shaped and U-shaped sclerotized structure anteriorly at its apex (Fig. 6R1–2)	***Acicnemis* sp. 1**
–	Elytra with white-scaled V-shaped or transverse band, but never an X-shaped band; aedeagus not as above	**19**
19	Brightly colored species, with dull orange, white, and brown scales; pedon with lateral edges approx. parallel in dorsal view, but converging evenly into a blunt, rounded point; internal sac not visible inside aedeagus, but protruding basally and with 2 sclerotized cylinder-shaped structures posteriorly at the base (Fig. 6M1–2)	***A. postica* Hubenthal, 1919**
–	Dark scaled species, with black and gray scales covering most of body; aedeagus not as above	**20**
20	Internal sac with tuberculate (roughened) musculature, visible inside of aedeagus; internal sac protruding from aedeagus and weakly hooked anteriorly (Fig. 6A1–2); large (5.0–6.8 mm), predominantly black species; northern mainland Japan, Hokkaido, Russia (Far East)	***A. albofasciata* (Ter-Minasian, 1953)**
–	internal sac not with tuberculate musculature visible inside of aedeagus (only in protruding portion of sac in some species); body size variable	**21**
21	internal sac with hook-shaped protruding structure and roughened musculature at the base (Fig. 6C1–2); pronotum with impunctate, longitudinal midline in many specimens; large species (4.9–7.0 mm), with pale to dark gray scales covering most of body ; mainland Japan………	***A. cryptica* Lewis & Kojima, sp. nov.**
–	Internal sac with modified protruding structure but not with roughened musculature and weakly hooked shape; pronotum never with impunctate longitudinal midline; size variable	**22**
22	Internal sac without club-shaped structure anteriorly at its apex (Fig. 6D1–2); pronotum with white, brown, yellow, and dark scales (with strong lateral bands in most specimens)	***A. dividicincta* Morimoto & Miyakawa, 1995**
–	Internal sac with club-shaped structure anteriorly at its apex; pronotum nearly uniform in color, with black and dark gray scales (no prominent lateral bands)	**23**
23	Club of internal sac surrounded closely by roughened musculature (Fig. 6J1–2); apex of pedon distinctly sinuate; black scales surrounding scutellum prominent on first and second elytral intervals	***A. nobilis* Hubenthal, 1919**
–	Club of internal sac not surrounded closely by roughened musculature (Fig. 6K1–2); apex of pedon barely sinuate; black scales surrounding scutellum prominent on first elytral interval, but only weakly extending onto the second elytral interval	***A. nohirai* Morimoto & Miyakawa, 1995**

## ﻿Discussion

The island country of Japan has experienced multiple, intermittent periods of land bridge connection with mainland Asia since the Miocene (25-15 MYA) (Tojo et al. 2017; Matsuzaki et al. 2022), and this complex geologic history is partially what accounts for the diverse faunal composition of the region. Another major factor accounting for the faunal diversity of Japan is the latitudinal breadth of the country, which extends northward from Hokkaido (45.52°N) south to the Ryukyu Islands (Hateruma-jima Island, 24.04°N) (Tojo et al. 2017). Although most of Japan falls within the Palearctic Region, the southern Ryukyu Islands lie on the Palearctic-Oriental Regions border (Komaki 2021), and Japan has seen influxes and mixing of fauna from both regions (Morimoto et al. 2006). The faunal composition of weevils (and presumably other phytophagous beetles) in Japan is particularly complicated by the recent discovery that weevil eggs can survive consumption by birds, pass through the gut, be deposited on neighboring islands, and develop without complication into mature adults (Lin et al. 2021). Furthermore, inter-island dispersal of some species has likely been facilitated by transport in fruits and seeds that are blown to sea (Yeh et al. 2018).

Herein, we reviewed the East Asian *Acicnemis* and uncovered three new species in mainland Japan. Although *Acicnemis* is most thoroughly studied in Japan, our study revealed that overlooked diversity stills exists even in this region. Two of our newly described species, *A. cryptica* (mainland Japan, excluding Hokkaido) and *A. koguma* (central mainland Japan), are cryptic and sister to more widespread, previously described species (*A. albofasciata*: Russia and mainland Japan (including Hokkaido); and *A. kiotoensis*: mainland Japan, Ryukyu Islands, Taiwan). The existence of overlooked *Acicnemis* diversity in a relatively well-studied region highlights the importance of extensive genital dissection across the entire geographic range of what otherwise may be assumed to be single species. In addition to dissection, we used DNA barcoding and X-ray μCT to successfully delineate species pairs. As dissection is not always an option when working with rare, fragile, or type material, these alternative non-destructive techniques can both potentially be incorporated in the delineation process as a complement to traditional morphological methods. Although DNA barcoding successfully delineated all species pairs treated in this work, X-ray μCT for sub-scale cuticular comparison was only informative when working with sufficiently distantly related species (e.g., *A. albofasciata* and *A. ryukyuana*). Although X-ray μCT has been successfully used to delineate weevils within the same genus by pronotal and elytral punctation (see Lewis et al. 2024), clearly the utility of X-ray μCT in species delineation and cryptic morphology discovery is highly dependent on the target group, a factor that should be considered when evaluating the suitability of the method in future works.

## Supplementary Material

XML Treatment for Acicnemis

XML Treatment for Acicnemis albofasciata

XML Treatment for Acicnemis azumai

XML Treatment for Acicnemis biarcuata

XML Treatment for Acicnemis bickhardti

XML Treatment for Acicnemis cordata

XML Treatment for Acicnemis costulifera

XML Treatment for Acicnemis cryptica

XML Treatment for Acicnemis dividicincta dividicincta

XML Treatment for Acicnemis dividicincta okinawana

XML Treatment for Acicnemis exilis

XML Treatment for Acicnemis kiotoensis

XML Treatment for Acicnemis koguma

XML Treatment for Acicnemis laeta

XML Treatment for Acicnemis luteomaculata

XML Treatment for Acicnemis maculaalba

XML Treatment for Acicnemis nobilis

XML Treatment for Acicnemis nohirai

XML Treatment for Acicnemis palliata

XML Treatment for Acicnemis postica

XML Treatment for Acicnemis ryukyuana

XML Treatment for Acicnemis sauteri

XML Treatment for Acicnemis shibatai

XML Treatment for Acicnemis shigematsui

XML Treatment for Acicnemis

XML Treatment for Acicnemis squamata

XML Treatment for Acicnemis suturalis

## ﻿Additional information

### Use of AI

No use of AI was reported.

### Author ORCIDs

Jake H. Lewis https://orcid.org/0000-0001-5147-7237

Erin O. Campbell https://orcid.org/0000-0001-8546-0636

### Data availability

All of the data that support the findings of this study are available in the main text or Supplementary Information.

## Supplementary materials

Supplementary material 1 K2P distances (CO1) for selected species covered in the manuscript Data type xls https://binary.pensoft.net/file/1461858This dataset is made available under the Open Database License (http://opendatacommons.org/licenses/odbl/1.0/). The Open Database License (ODbL) is a license agreement intended to allow users to freely share, modify, and use this Dataset while maintaining this same freedom for others, provided that the original source and author(s) are credited.Jake H. Lewis, Hiroaki Kojima, Erin O. Campbell
